# Supplementary material for: Topological heavy fermions in magnetic field
Source: Nat Commun. 2024 Jun 19;15:5257. doi: 10.1038/s41467-024-49531-3 (PMC11187166; doi:10.1038/s41467-024-49531-3)
Supplement: Supplementary file 1 — Supplementary Information [file 41467_2024_49531_MOESM1_ESM.pdf]

Supplementary Information: Topological heavy fermions in magnetic field

Keshav Singh[1,2] , Aaron Chew[3] , Jonah Herzog-Arbeitman[3] , B. Andrei Bernevig[3,4,5] and Oskar Vafek[1,2]
[1] *National High Magnetic Field Laboratory, Tallahassee, Florida, 32310*
[2] *Department of Physics, Florida State University, Tallahassee, Florida, 32306*
[3] *Department of Physics, Princeton University, Princeton, NJ 08544*
[4] *Donostia International Physics Center, P. Manuel de Lardizabal 4, 20018 Donostia-San Sebastian, Spain*
[5] *IKERBASQUE, Basque Foundation for Science, Bilbao, Spain*

CONTENTS

Supplementary note 1. Bistritzer-MacDonald model 2

Supplementary note 2. Magnetic Translation Operators 2
A. Landau gauge magnetic translation group identities 3

Supplementary note 3. Finite **B** basis for the c and f fermions 3

Supplementary note 4. Computation of Matrix elements 8
A. c-c Coupling 8
B. c-f Coupling 10
1. Non-derivative or the zeroth-order coupling γ 14
2. k_x coupling 14
3. k_y coupling 15
4. Minimal coupling 15
5. Closed form expression for c-f coupling at finite field 16
C. Low field Analysis of c-f coupling 17

Supplementary note 5. Singular Values for I^0 19

Supplementary note 6. Discussion For Valley **K** 23
A. CNP 23
B. $\nu = \pm 1$, Spin ↑ 25
C. $\nu = \pm 1$, Spin ↓ 26
D. $\nu = \pm 2$, Spin ↑↓ 28

Supplementary note 7. Discussion For Valley **K**′ 29
A. CNP 30
B. $\nu = \pm 1$ Spin ↑↓ 32
C. $\nu = \pm 2$ Spin ↑↓ 34

Supplementary note 8. Naive Minimal Substitution 36

Supplementary note 9. Parent Kramers inter-valley coherent state 37
A. CNP 37
B. $\nu = -2$ 38
1. Spin ↑ 38

Supplementary note 10. Effective Hamiltonians for Landau Quantization of light modes 39
A. CNP 39
1. Parent Valley Polarized State 40
2. Parent Kramers Intervalley Coherent State 43
B. $\nu = -1$ 45
1. Parent Valley Polarized State 45
C. $\nu = -2$ 50
1. Parent Valley Polarized State 50

2. Parent Kramers Intervalley Coherent State 52

Supplementary note 11. Review of Magnetic Bloch's Theorem at 2π Flux 54

Supplementary note 12. Rational Flux 55
A. Groundstate Charge Density 58
B. Many-body Charge Excitations 61

Supplementary note 13. Notational Changes 62

References 63

Supplementary note 1. BISTRITZER-MACDONALD MODEL

The Bistritzer-Macdonald (BM) model of magic-angle twisted bilayer graphene[1] in the valley $\mathbf{K}(\tau=+1)$ is

$$H^{\mathbf{K}}_{BM}(\mathbf{p}) = \begin{pmatrix} v_F\sigma\cdot\mathbf{p} & T(\mathbf{r})e^{i\mathbf{q}_1\cdot\mathbf{r}} \\ e^{-i\mathbf{q}_1\cdot\mathbf{r}}T^\dagger(\mathbf{r}) & v_F\sigma\cdot(\mathbf{p}+\hbar\mathbf{q}_1) \end{pmatrix}, \tag{1}$$

where σ acts in sublattice space and $\mathbf{p}=0$ denotes the moiré Dirac point K_M. The interlayer hopping functions are

$$T(\mathbf{r}) = \sum_{j=1}^{3} T_j e^{-i\mathbf{q}_1\cdot\mathbf{r}}, \tag{2}$$

where $\mathbf{q}_1 = k_\theta(0,-1)$, $\mathbf{q}_{2,3} = k_\theta(\pm\frac{\sqrt{3}}{2},\frac{1}{2})$, $k_\theta = \frac{8\pi}{3a_0}\sin\frac{\theta}{2} = \frac{4\pi}{3L_m}$, $a_0 \approx 0.246$nm, $L_m = \frac{a_0}{2\sin\theta/2}$ is moiré period. $\theta = 1.05°$ in this work.

$$T_{j+1} = w_0 I_2 + w_1\left(\cos\left(\frac{2\pi}{3}j\right)\sigma_x + \sin\left(\frac{2\pi}{3}j\right)\sigma_y\right) \tag{3}$$

where I_n is the $n\times n$ unit matrix. We will present results for the case $w_0/w_1 = 0.7$. The BM model is invariant under translation by integer multiples of moiré lattice vectors $\mathbf{L}_1 = L_m(\frac{\sqrt{3}}{2},\frac{1}{2})$ and $\mathbf{L}_2 = L_m(0,1)$. Thus if $f(\mathbf{r})$ is an eigenstate, then so is

$$\hat{T}_{\mathbf{L}_{1,2}} f(\mathbf{r}) = f(\mathbf{r}-\mathbf{L}_{1,2}) \tag{4}$$

where $\hat{T}_{\mathbf{L}_{1,2}}$ are the usual discrete translation operators.

Supplementary note 2. MAGNETIC TRANSLATION OPERATORS

At $\mathbf{B}\neq 0$ in the Landau gauge $\mathbf{A}=(0,Bx)$, the BM model in flux is found via minimally substitution: $H^{\mathbf{K}}_{BM}\left(\mathbf{p}-\frac{e}{c}\mathbf{A}\right) = H^{\mathbf{K}}_{BM}\left(p_x, p_y - \frac{eB}{c}x\right)$. The finite $\mathbf{B}$ BM model at $\mathbf{K}'(\tau=-1)$ can be obtained similarly by applying time reversal to Eq.(1) followed by the minimal substitution. However at $\mathbf{B}\neq 0$, H^{τ}_{BM} is still invariant under the translation by $\mathbf{L}_2$, but a translation by $\mathbf{L}_1$ needs to be accompanied by a gauge transformation as

$$e^{i\frac{eB}{\hbar c}L_{1x}y} H^{\tau}_{BM}\left(p_x, p_y - \frac{eB}{c}x + \frac{eB}{c}L_{1x}\right) e^{-i\frac{eB}{\hbar c}L_{1x}y} = H^{\tau}_{BM}\left(p_x, p_y - \frac{eB}{c}x\right). \tag{5}$$

In other words if $\tilde{f}(\mathbf{r})$ is an eigenstate of $H^{\tau}_{BM}\left(p_x, p_y - \frac{eB}{c}x\right)$, then so is

$$\tilde{f}(\mathbf{r}) \to \hat{t}_{\mathbf{L}_1}\tilde{f}(\mathbf{r}) = e^{i\frac{eB}{\hbar c}L_{1x}y}\tilde{f}(\mathbf{r}-\mathbf{L}_1) \tag{6}$$

Thus $\hat{t}_{\mathbf{L}_1} = e^{i\frac{eB}{\hbar c}L_{1x}y}\hat{T}_{\mathbf{L}_1}$ is the generator of magnetic translation by $\mathbf{L}_1$. Note that $\hat{t}_{L_1}$ can alternatively be presented as

$$\hat{t}_{\mathbf{L}_1} = e^{i\mathbf{q}_\phi\cdot\mathbf{r}}\hat{T}_{\mathbf{L}_1} \tag{7}$$

where the magnetic translation wavevector $\mathbf{q}_\phi$ is defined as

$$\mathbf{q}_\phi = \frac{\phi}{\phi_0}\left(\frac{1}{2}\mathbf{g}_1 + \mathbf{g}_2\right) \tag{8}$$

where $\mathbf{g}_{1,2}$ are corresponding reciprocal lattice vectors to $\mathbf{L}_{1,2}$ and are given as: $\mathbf{g}_1 = \frac{4\pi}{\sqrt{3}L_m}(1,0)$ and $\mathbf{g}_2 = \frac{4\pi}{\sqrt{3}L_m}(-\frac{1}{2},\frac{\sqrt{3}}{2})$. Hence

$$\hat{t}_{\mathbf{L}_1} = e^{2\pi i \frac{\phi}{\phi_0}\frac{y}{L_2}}\hat{T}_{\mathbf{L}_1}, \tag{9}$$

where $L_2 = \mid \mathbf{L}_2 \mid = L_m$. Magnetic translation by $\mathbf{L}_2$ are generated by $\hat{t}_{\mathbf{L}_2}\tilde{f}(\mathbf{r}) = \tilde{f}(\mathbf{r}-\mathbf{L}_2)$. We thus have

$$\hat{t}_{\mathbf{L}_2}\hat{t}_{\mathbf{L}_1}\tilde{f}(\mathbf{r}) = e^{i\frac{eB}{\hbar c}L_{1x}(y-L_2)}\tilde{f}(\mathbf{r}-\mathbf{L}_1-\mathbf{L}_2) \tag{10}$$

$$\hat{t}_{\mathbf{L}_1}\hat{t}_{\mathbf{L}_2}\tilde{f}(\mathbf{r}) = e^{i\frac{eB}{\hbar c}L_{1x}y}\tilde{f}(\mathbf{r}-\mathbf{L}_1-\mathbf{L}_2) \tag{11}$$

$$\Rightarrow \hat{t}_{\mathbf{L}_2}\hat{t}_{\mathbf{L}_1} = e^{-2\pi i\frac{\phi}{\phi_0}}\hat{t}_{\mathbf{L}_1}\hat{t}_{\mathbf{L}_2} \tag{12}$$

where $\phi_0 = \frac{hc}{e}$ and the flux through the unit cell is $\phi = BL_{1x}L_2$. If $\phi/\phi_0 = p/q$, with p and q relatively prime integers,

$$\left[\hat{t}^q_{\mathbf{L}_2}, \hat{t}_{\mathbf{L}_1}\right] = 0 \tag{13}$$

and

$$\left[\hat{t}_{\mathbf{L}_{1,2}}, H^\tau_{BM}\left(p_x, p_y - \frac{eB}{c}x\right)\right] = 0. \tag{14}$$

The simultaneous eigenstates of the magnetic translation operators $\hat{t}_{\mathbf{L}_1}$ and $\hat{t}^q_{\mathbf{L}_2}$ can thus be used to produce a complete and orthonormal set of basis states for solving the $\mathbf{B} \neq 0$ BM model. As we will see below, depending on whether they are conduction or heavy fermions there will be p states or q states per momentum point. It will be helpful to derive an identity for $\hat{t}^s_{\mathbf{L}_1}$ which relates it to $\hat{T}^s_{\mathbf{L}_1}$.

A. Landau gauge magnetic translation group identities

Using Eq.(9), we have

$$\hat{t}^s_{\mathbf{L}_1} = \left(e^{2\pi i\frac{\phi}{\phi_0}\frac{y}{L_2}}\hat{T}_{\mathbf{L}_1}\right)^s = \left(e^{2\pi i\frac{\phi}{\phi_0}\frac{y}{L_2}}\hat{T}_{\mathbf{L}_1}\right)\ldots\left(e^{2\pi i\frac{\phi}{\phi_0}\frac{y}{L_2}}\hat{T}_{\mathbf{L}_1}\right)\left(e^{2\pi i\frac{\phi}{\phi_0}\frac{y}{L_2}}\hat{T}_{\mathbf{L}_1}\right)\left(e^{2\pi i\frac{\phi}{\phi_0}\frac{y}{L_2}}\hat{T}_{\mathbf{L}_1}\right)\left(e^{2\pi i\frac{\phi}{\phi_0}\frac{y}{L_2}}\hat{T}_{\mathbf{L}_1}\right) \tag{15}$$

$$= \left(e^{2\pi i\frac{\phi}{\phi_0}\frac{y}{L_2}}\hat{T}_{\mathbf{L}_1}\right)\ldots\left(e^{2\pi i\frac{\phi}{\phi_0}\frac{y}{L_2}}\hat{T}_{\mathbf{L}_1}\right)\left(e^{2\pi i\frac{\phi}{\phi_0}\frac{y}{L_2}}\hat{T}_{\mathbf{L}_1}\right)\left(e^{2\pi i\frac{\phi}{\phi_0}\frac{2y-L_{1y}}{L_2}}\hat{T}^2_{\mathbf{L}_1}\right) \tag{16}$$

$$= \left(e^{2\pi i\frac{\phi}{\phi_0}\frac{y}{L_2}}\hat{T}_{\mathbf{L}_1}\right)\ldots\left(e^{2\pi i\frac{\phi}{\phi_0}\frac{y}{L_2}}\hat{T}_{\mathbf{L}_1}\right)\left(e^{2\pi i\frac{\phi}{\phi_0}\frac{3y-2L_{1y}-L_{1y}}{L_2}}\hat{T}^3_{\mathbf{L}_1}\right) \tag{17}$$

$$= \left(e^{2\pi i\frac{\phi}{\phi_0}\frac{y}{L_2}}\hat{T}_{\mathbf{L}_1}\right)\ldots\left(e^{2\pi i\frac{\phi}{\phi_0}\frac{y}{L_2}}\hat{T}_{\mathbf{L}_1}\right)\left(e^{2\pi i\frac{\phi}{\phi_0}\frac{4y-3L_{1y}-2L_{1y}-L_{1y}}{L_2}}\hat{T}^4_{\mathbf{L}_1}\right). \tag{18}$$

Now, $1+2+3+\ldots+(s-1) = \frac{1}{2}s(s-1)$. Therefore,

$$\hat{t}^s_{\mathbf{L}_1} = e^{-\pi i s(s-1)\frac{p}{q}\frac{L_{1y}}{L_2}}e^{2\pi i s\frac{p}{q}\frac{y}{L_2}}\hat{T}^s_{\mathbf{L}_1} \tag{19}$$

where $\phi/\phi_0 = p/q$. Although the above formula was derived for positive s, it also holds for negative s. This can be seen by noting that

$$\left(e^{-\pi i s(s-1)\frac{\phi}{\phi_0}\frac{L_{1y}}{L_2}}e^{i\frac{eB}{\hbar c}sL_{1x}y}\hat{T}^s_{\mathbf{L}_1}\right)\left(e^{\pi i s(-s-1)\frac{\phi}{\phi_0}\frac{L_{1y}}{L_2}}e^{-i\frac{eB}{\hbar c}sL_{1x}y}\hat{T}^{-s}_{\mathbf{L}_1}\right) = 1. \tag{20}$$

So, $\hat{t}^{-1}_{\mathbf{L}_1}$ denotes the inverse of $\hat{t}_{\mathbf{L}_1}$, and $\hat{t}^{-s}_{\mathbf{L}_1} = \left(\hat{t}^{-1}_{\mathbf{L}_1}\right)^s$.

Supplementary note 3. FINITE B BASIS FOR THE c AND f FERMIONS

The $\mathbf{B} = 0$ basis for f fermions is constituted by two AA-stacking localised Wannier states per valley per spin [2]. We denote them as $W_{\mathbf{0},b\tau}(\mathbf{r})$, for $b \in \{1,2\}$ and $\tau = \pm 1$. Using the recently introduced method [3], we can construct

the finite $\mathbf{B}$ basis by first building hybrid Wannier states out of the zero field Wannier states followed by projection onto a representation of magnetic translation group(MTG). For given spin, the hybrid Wannier states read

$$w_{b\tau}(\mathbf{r},k_2\mathbf{g}_2)=\sum_{n\in\mathbb{Z}}e^{ik_2\mathbf{g}_2\cdot(n\mathbf{L}_2)}\hat{t}^n_{\mathbf{L}_2}W_{\mathbf{0},b\tau}(\mathbf{r})=\sum_{n\in\mathbb{Z}}e^{2\pi ik_2n}W_{0,b\tau}(\mathbf{r}-n\mathbf{L}_2). \tag{21}$$

where $k_2\in[0,1)$. These hybrid Wannier states although localized along the x direction are Bloch-like extended in the y direction and thus eigenstates of $\hat{t}_{\mathbf{L}_2}$

$$\hat{t}_{\mathbf{L}_2}w_{b\tau}(\mathbf{r},k_2\mathbf{g}_2)=w_{b\tau}(\mathbf{r}-\mathbf{L}_2,k_2\mathbf{g}_2)=e^{-2\pi ik_2}w_{b\tau}(\mathbf{r},k_2\mathbf{g}_2). \tag{22}$$

We next construct normalized eigenstates of MTG out of the hybrid Wannier states as

$$\eta_{b\tau k_1k_2}(\mathbf{r})=\frac{1}{\sqrt{\mathcal{N}}}\sum_{s\in\mathbb{Z}}e^{2\pi isk_1}\left(\hat{t}^s_{\mathbf{L}_1}w_{b\tau}(\mathbf{r},k_2\mathbf{g}_2)\right), \tag{23}$$

where $k_1\in[0,1)$ and normalization $\mathcal{N}=n_{tot}s_{tot}$. Here s_{tot} and n_{tot} denote the total count of s and n. Note that although these summations should be unbounded, we require these cutoffs for defining the normalization of our states and intermediate steps. They eventually cancel in the final formulas are are not physical. The normalisation is justified in Eqs.(27)-(29). The domains of $k_{1,2}$ can be understood via noting that $\eta_{b\tau k_1k_2}$ is periodic under $k_{1,2}\to k_{1,2}+1$. Under magnetic translations $\hat{t}_{\mathbf{L}_1}$ and $\hat{t}^q_{\mathbf{L}_2}$, we then have

$$\hat{t}_{\mathbf{L}_1}\eta_{b\tau k_1k_2}=e^{-2\pi ik_1}\eta_{b\tau k_1k_2} \tag{24}$$
$$\hat{t}^q_{\mathbf{L}_2}\eta_{b\tau k_1k_2}=e^{-2\pi iqk_2}\eta_{b\tau k_1k_2}. \tag{25}$$

Thus states with k_2 that differ by $1/q$ have the same eigenvalue under $\hat{t}^q_{\mathbf{L}_2}$. This is because the qL_2 translations break up the k_2 domains into units of width $1/q$. To make it apparent from the quantum numbers, we relabel the states as

$$\eta_{b\tau kr'}(\mathbf{r})=\eta_{b\tau k_1k_2+\frac{r'}{q}}(\mathbf{r})\ \ ,\ \ k=(k_1,k_2)\in[0,1)\otimes[0,1/q)\ \ ,\ \ r'=0,1\ldots q-1. \tag{26}$$

Thus $\eta_{b\tau kr'}(\mathbf{r})$ with different k quantum numbers are guaranteed to be orthogonal. Moreover we have q states for given k, corresponding to $r'\in\{0\ldots q-1\}$ for each $b\in\{1,2\}$, i.e. a total of $2q$ heavy fermion states for given k. For same k and different r' the overlaps for given valley are

$$\begin{aligned}&\int d^2\mathbf{r}\eta^*_{b\tau kr'_1}(\mathbf{r})\eta_{b'\tau kr'_2}(\mathbf{r})=\\&\frac{1}{\mathcal{N}}\sum_{s_1s_2\in\mathbb{Z}}\sum_{n_1n_2\in\mathbb{Z}}e^{2\pi i(s_2-s_1)k_1}e^{-2\pi i(k_2+\frac{r'_1}{q})n_1}e^{2\pi i(k_2+\frac{r'_2}{q})n_2}e^{\pi is_1(s_1-1)\frac{p}{q}\frac{L_{1y}}{L_2}}e^{-\pi is_2(s_2-1)\frac{p}{q}\frac{L_{1y}}{L_2}}\\&\times\int d^2\mathbf{r}e^{2\pi i(s_2-s_1)\frac{p}{q}\frac{y}{L_2}}W^*_{\mathbf{0}b\tau}(\mathbf{r}-s_1\mathbf{L}_1-n_1\mathbf{L}_2)W_{\mathbf{0}b'\tau}(\mathbf{r}-s_2\mathbf{L}_1-n_2\mathbf{L}_2).\end{aligned} \tag{27}$$

Now since the $\mathbf{B}=0$ Wannier states are well localised and orthonormal, we have

$$\int d^2\mathbf{r}e^{2\pi i(s_2-s_1)\frac{p}{q}\frac{y}{L_2}}W^*_{\mathbf{0}b\tau}(\mathbf{r}-s_1\mathbf{L}_1-n_1\mathbf{L}_2)W_{\mathbf{0}b'\tau}(\mathbf{r}-s_2\mathbf{L}_1-n_2\mathbf{L}_2)\approx\delta_{bb'}\delta_{s_1s_2}\delta_{n_1n_2} \tag{28}$$

and thus,

$$\int d^2\mathbf{r}\eta^*_{b\tau kr'_1}(\mathbf{r})\eta_{b'\tau kr'_2}(\mathbf{r})\approx\delta_{bb'}\frac{1}{\mathcal{N}}\sum_{s_1\in\mathbb{Z}}\sum_{n_1\in\mathbb{Z}}e^{2\pi i\frac{r'_2-r'_1}{q}n_1}=\delta_{r'_1r'_2}\delta_{bb'}\frac{s_{tot}n_{tot}}{\mathcal{N}}=\delta_{r'_1r'_2}\delta_{bb'}. \tag{29}$$

Henceforth we have a complete orthonormal basis for the heavy fermions in finite $\mathbf{B}$, constituted by $2q$ states for given k, i.e. 2 states per moiré unit cell per valley per spin, which clearly implies that these are total Chern 0 states.

Now let us discuss the basis for c fermions. Remember that at $\mathbf{B}=0$, the basis for c fermions is constituted by four $\mathbf{k}\cdot\mathbf{p}$ Bloch states at the Γ point in the moiré Brillouin Zone(mBZ), per valley per spin. We denote them by $\tilde{\Psi}_{\Gamma a\tau}$ for $a\in\{1\ldots4\}$ and $\tau=\pm1$.

We promote the $\mathbf{B}=0$ c fermion basis to finite $\mathbf{B}$ using a result obtained when the $\mathbf{k}\cdot\mathbf{p}$ method is extended to finite $\mathbf{B}$[4], which prescribes the ansatz for finite field $\mathbf{k}\cdot\mathbf{p}$ states to be Landau level(LL) coefficients on top of the zero field $\mathbf{k}\cdot\mathbf{p}$ states. Henceforth, we define the finite $\mathbf{B}$ basis for $\mathbf{k}\cdot\mathbf{p}$ Bloch states as an expansion over product of

LLs and the $\mathbf{B}=0$ $\mathbf{k}\cdot\mathbf{p}$ Bloch states at the Γ point in mBZ. However in order to use the same quantum numbers label as finite $\mathbf{B}$ f fermion basis we first project the Landau gauge LL onto the representation of MTG as

$$\chi_{k_1k_2m}(\mathbf{r}) = \frac{1}{\sqrt{\ell L_2}}\frac{1}{\sqrt{\mathcal{N}}}\sum_{s\in\mathbb{Z}} e^{2\pi i s k_1}\left(\hat{t}^s_{\mathbf{L}_1} e^{2\pi i k_2 \frac{y}{L_2}}\varphi_m\left(x - k_2\frac{2\pi\ell^2}{L_2}\right)\right) \tag{30}$$

where $k_1 \in [0,1)$ and unlike before, $k_2 \in [0, p/q)$ as justified in Eqs.(33)-(36). The functions φ_m are mth 1D harmonic oscillator states that determine the Landau gauge Landau levels

$$\varphi_m(x) = \frac{1}{\pi^{\frac{1}{4}}}\frac{1}{\sqrt{2^m m!}} e^{-x^2/2\ell^2} H_m(x/\ell) \tag{31}$$

where H_m is the Hermite polynomial and $\ell^2 = \hbar c/(eB)$. The normalization of the LL MTG states is explained in Eqs.(43)-(48). The domains for the quantum numbers $k_{1,2}$ can be understood by noting that $\chi_{k_1k_2m}$ is periodic under $k_1 \to k_1+1$ and up to a phase under $k_2 \to k_2 + p/q$ as shown below

$$\chi_{k_1k_2+\frac{p}{q}m} = \frac{1}{\sqrt{\ell L_2}}\frac{1}{\sqrt{\mathcal{N}}}\sum_{s\in\mathbb{Z}} e^{2\pi i s k_1}\left(\hat{t}^s_{\mathbf{L}_1} e^{2\pi i (k_2+\frac{p}{q})\frac{y}{L_2}}\varphi_m\left(x - (k_2+\frac{p}{q})\frac{2\pi\ell^2}{L_2}\right)\right), \tag{32}$$

now using the fact, $\frac{2\pi\ell^2}{L_2}\frac{p}{q} = L_{1x}$ we have

$$\chi_{k_1k_2+\frac{p}{q}m} = \frac{1}{\sqrt{\ell L_2}}\frac{1}{\sqrt{\mathcal{N}}}\sum_{s\in\mathbb{Z}} e^{2\pi i s k_1}\left(\hat{t}^s_{\mathbf{L}_1} e^{2\pi i (k_2+\frac{p}{q})\frac{y}{L_2}}\varphi_m\left(x - L_{1x} - k_2\frac{2\pi\ell^2}{L_2}\right)\right) \tag{33}$$

$$= e^{2\pi i k_2 \frac{L_{1y}}{L_2}}\frac{1}{\sqrt{\ell L_2}}\frac{1}{\sqrt{\mathcal{N}}}\sum_{s\in\mathbb{Z}} e^{2\pi i s k_1}\left(\hat{t}^s_{\mathbf{L}_1} e^{2\pi i \frac{p}{q}\frac{y}{L_2}}\hat{T}_{\mathbf{L}_1}\left(e^{2\pi i k_2\frac{y}{L_2}}\varphi_m\left(x - k_2\frac{2\pi\ell^2}{L_2}\right)\right)\right) \tag{34}$$

$$= e^{2\pi i k_2 \frac{L_{1y}}{L_2}}\frac{1}{\sqrt{\ell L_2}}\frac{1}{\sqrt{\mathcal{N}}}\sum_{s\in\mathbb{Z}} e^{2\pi i s k_1}\left(\hat{t}^{s+1}_{\mathbf{L}_1} e^{2\pi i k_2\frac{y}{L_2}}\varphi_m\left(x - k_2\frac{2\pi\ell^2}{L_2}\right)\right) \tag{35}$$

$$= e^{-2\pi i\left(k_1 - k_2\frac{L_{1y}}{L_2}\right)}\chi_{k_1k_2m} \tag{36}$$

The domain for k_2 can also be understood in the context of the fact that $\chi_{k_1k_2m}$ are essentially LLs and thus behave as Chern number $+1$ states in $\mathbf{B}\neq 0$. In order to illustrate it, let us discuss the total number of available states for $\chi_{k_1k_2m}$. We start by noting that under magnetic translations $\hat{t}_{\mathbf{L}_1}$ and $\hat{t}^q_{\mathbf{L}_2}$

$$\hat{t}_{\mathbf{L}_1}\chi_{k_1k_2m} = e^{-2\pi i k_1}\chi_{k_1k_2m}, \tag{37}$$

$$\hat{t}^q_{\mathbf{L}_2}\chi_{k_1k_2m} = e^{-2\pi i q k_2}\chi_{k_1k_2m}. \tag{38}$$

If we have a system size $N_1\mathbf{L}_1$ and $qN_2\mathbf{L}_2$, then

$$\hat{t}^{N_1}_{\mathbf{L}_1}\chi_{k_1k_2m} = \chi_{k_1k_2m} \tag{39}$$

$$\Rightarrow e^{-2\pi i k_1 N_1} = 1;\ \Rightarrow\quad k_1 = 0, \frac{1}{N_1}, \frac{2}{N_1}, \ldots, 1 - \frac{1}{N_1} \tag{40}$$

Similarly,

$$\hat{t}^{qN_2}_{\mathbf{L}_2}\chi_{k_1k_2m} = \chi_{k_1k_2m} \tag{41}$$

$$\Rightarrow e^{-2\pi i k_2 q N_2} = 1;\ \Rightarrow\quad k_2 = 0, \frac{1}{qN_2}, \frac{2}{qN_2}, \ldots, \frac{p}{q} - \frac{1}{qN_2} \tag{42}$$

So, the total number of states is $N_1\frac{p}{q}qN_2 = \frac{\phi}{\phi_0}N_1qN_2 = \frac{\phi}{\phi_0}N_1qN_2\frac{L_{1x}L_2}{A_{uc}} = \frac{\phi}{\phi_0}\frac{A_{tot}}{A_{uc}} = \frac{B}{\phi_0}A_{tot} = \frac{\phi_{tot}}{\phi_0}$. Here $A_{uc} = \hat{z}\cdot(\mathbf{L}_1\times\mathbf{L}_2)$ denotes the area of moiré unit cell. Thus qA_{uc} is the area of magnetic unit cell and total area $A_{tot} = N_1N_2qA_{uc}$. This is a well known result for the degeneracy of the Landau level. Let us now discuss the

orthonormalization for the LL MTG eigenstates. The overlaps amidst the MTG LL are given as:

$$\int d^2\mathbf{r}\chi^*_{k_1k_2m}(\mathbf{r})\chi_{k'_1k'_2m'}(\mathbf{r}) = \tag{43}$$

$$\frac{1}{\ell L_2\mathcal{N}}\sum_{s\in\mathbb{Z}}\sum_{s'\in\mathbb{Z}} e^{-2\pi is\left(k_1-\left(k_2-\frac{p}{q}\right)\frac{L_{1y}}{L_2}\right)} e^{i\pi s(s+1)\frac{p}{q}\frac{L_{1y}}{L_2}} e^{2\pi is'\left(k'_1-\left(k'_2-\frac{p}{q}\right)\frac{L_{1y}}{L_2}\right)} e^{-i\pi s'(s'+1)\frac{p}{q}\frac{L_{1y}}{L_2}}$$

$$\int dye^{-iy\left(s\frac{p}{q}+k_2\right)\frac{2\pi}{L_2}} e^{iy\left(s'\frac{p}{q}+k'_2\right)\frac{2\pi}{L_2}} \int dx\varphi_m\left(x-\left(s\frac{p}{q}+k_2\right)\frac{2\pi}{L_2}\ell^2\right)\varphi_{m'}\left(x-\left(s'\frac{p}{q}+k'_2\right)\frac{2\pi}{L_2}\ell^2\right) \tag{44}$$

$$= \frac{1}{\ell L_2\mathcal{N}}\sum_{s\in\mathbb{Z}}\sum_{s'\in\mathbb{Z}} e^{-2\pi is\left(k_1-\left(k_2-\frac{p}{q}\right)\frac{L_{1y}}{L_2}\right)} e^{i\pi s(s+1)\frac{p}{q}\frac{L_{1y}}{L_2}} e^{2\pi is'\left(k'_1-\left(k'_2-\frac{p}{q}\right)\frac{L_{1y}}{L_2}\right)} e^{-i\pi s'(s'+1)\frac{p}{q}\frac{L_{1y}}{L_2}}$$

$$n_{tot}L_2\delta_{s,s'}\delta_{k_2,k'_2}\ell\delta_{m,m'} \tag{45}$$

$$= \frac{1}{L_2\mathcal{N}}\sum_{s\in\mathbb{Z}} e^{2\pi is(k_1-k'_1)} n_{tot}L_2\delta_{k_2,k'_2}\delta_{m,m'} \tag{46}$$

$$= \frac{1}{L_2\mathcal{N}} s_{tot}\delta_{k_1,k'_1} n_{tot}L_2\delta_{k_2,k'_2}\delta_{m,m'} \tag{47}$$

$$= \delta_{k_1,k'_1}\delta_{k_2,k'_2}\delta_{m,m'}. \tag{48}$$

where in Eq.(44)-(45), we have used the fact that y-integral implies $k_2-k'_2=(s'-s)\frac{p}{q}$. Now since $k_2,k'_2\in[0,\frac{p}{q})$, the integral evaluates to $n_{tot}L_2\delta_{k_2,k'_2}\delta_{s,s'}$. Moreover each harmonic oscillator function in the x-integral can be shifted by the same amount since the arguments coincide. Therefore the x-integral evaluates to $\ell\delta_{mm'}$. Eventually in Eq.(46)-(47), the s summation implies $k_1-k'_1=$ integer. But since $k_1,k'_1\in[0,1)$, we must have $k_1=k'_1$ and the summation evaluates to $s_{tot}\delta_{k_1,k'_1}$. Therefore χ's are orthogonal and normalized. To have the same quantum number label as that of $\mathbf{B}\neq 0$ f fermion states, we relabel the MTG LLs as

$$\chi_{krm}(\mathbf{r}) = \chi_{k_1k_2+\frac{r}{q}m}(\mathbf{r}) \;\;,\;\; k=(k_1,k_2)\in[0,1)\otimes[0,1/q) \;\;,\;\; r=0,1\ldots p-1. \tag{49}$$

Thus for given k, we have p states, corresponding to $r\in\{0\ldots p-1\}$, i.e. $\frac{p}{q}$ states per moiré unit cell per valley per spin as expected. Finally we have the finite $\mathbf{B}$ c fermion basis states as $\Psi_{a\tau}\chi_{krm}$, where

$$\Psi_{a\tau} = \sqrt{NA_{uc}}\,\tilde{\Psi}_{\Gamma a\tau} \tag{50}$$

with N being the total number of moiré unit cells. The $\sqrt{NA_{uc}}$ factor is required due to normalization of χ. Note that since $\Psi_{a\tau}$ is a Bloch state at Γ,

$$\hat{T}_{\mathbf{L}_i}\,\Psi_{a\tau}(\mathbf{r}) = \Psi_{a\tau}(\mathbf{r}) \tag{51}$$

$$\implies \hat{t}_{\mathbf{L}_i}\left(\Psi_{a\tau}(\mathbf{r})\chi_{krm}\right)(\mathbf{r}) = \Psi_{a\tau}\hat{t}_{\mathbf{L}_i}\chi_{krm}(\mathbf{r}), \tag{52}$$

where $i\in\{1,2\}$. Thus $\Psi_{a\tau}\chi_{krm}$ are MTG eigenstates with same eigenvalue as that of χ_{krm}. Now let us discuss the orthonormalization for these states. Since in this work we focus on $\frac{1}{q}$ sequence, for the following discussion on orthonormality we set index r in χ_{krm} to zero and work with χ_{k0m}. The Bloch periodicity for $\tilde{\Psi}_{\Gamma a\tau}(\mathbf{r})$ allows us to expand it as

$$\tilde{\Psi}_{\Gamma a\tau}(\mathbf{r}) = \frac{1}{\sqrt{NA_{uc}}}\sum_{\mathbf{G}} e^{i\mathbf{G}\cdot\mathbf{r}}C_{a\tau\mathbf{G}}, \tag{53}$$

where $\mathbf{G}=n_1\mathbf{g}_1+n_2\mathbf{g}_2$, $n_{1,2}\in\mathbb{Z}$, denote moiré reciprocal lattice vectors and

$$C_{a\tau\mathbf{G}} = \frac{1}{\sqrt{NA_{uc}}}\int d^2\mathbf{r}e^{-i\mathbf{G}\cdot\mathbf{r}}\,\tilde{\Psi}_{\Gamma a\tau}(\mathbf{r}) \tag{54}$$

can be found in [5]. Now using Eq.(50), the overlap matrix can be given as

$$O^{(q,m_{max})}_{[m',a'][m,a]} = \int d^2\mathbf{r}\left(\Psi_{a'\tau}\chi_{k'0m'}\right)^*\Psi_{a\tau}\chi_{k0m} = \sum_{\mathbf{G}_1,\mathbf{G}_2} C^*_{a'\tau\mathbf{G}_1}C_{a\tau\mathbf{G}_2}\int d^2\mathbf{r}e^{i(\mathbf{G}_2-\mathbf{G}_1)\cdot\mathbf{r}}\chi^*_{k'0m'}\chi_{k0m}, \tag{55}$$

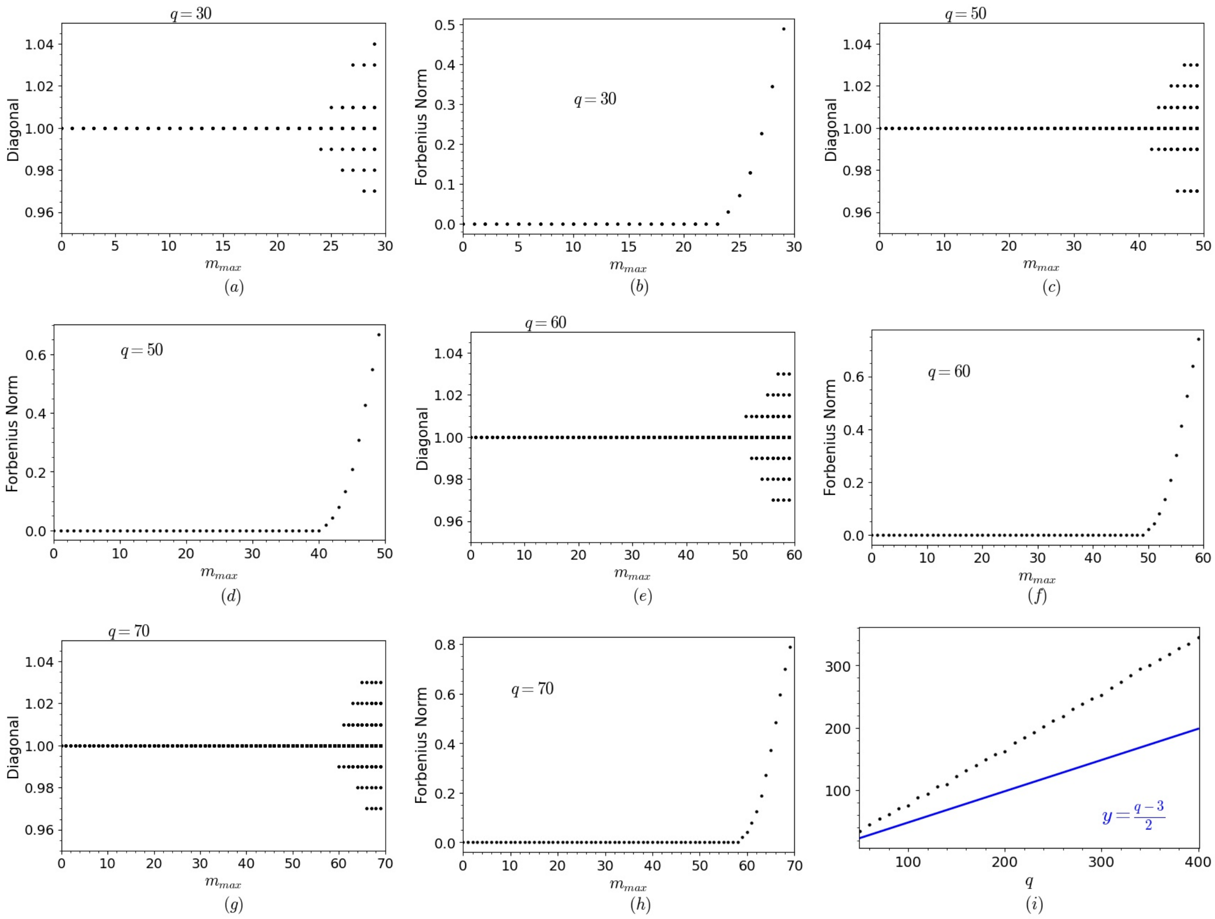

Supplementary Figure 1. Figures showing the diagonal values of Overlap matrix $O^{(q,m_{max})}_{[m',a'][m,a]}$ and frobenius norm of $O^{(q,m_{max})} - \mathbb{I}_{m_{max}}$ $\forall$ $m_{max} = 0 \dots q-1$ at $w_0/w_1 = 0.8$ for (a,b) $q = 30$, (c,d) $q = 50$, (e,f) $q = 60$ and (g,h) $q = 70$ respectively. For higher values of q, the value of m_{max} for which diagonal values start deviating from 1 have been (i) plotted using $\bullet$.

where $1/q$ is the flux per unit cell per flux quantum ϕ_0 and m_{max} denotes the upper cutoff on the indices m and m'. The integral can be computed as:

$$\begin{aligned}
&\int d^2\mathbf{r} e^{i\mathbf{g}\cdot\mathbf{r}} \chi^*_{k'0m'}(\mathbf{r})\chi_{k0m}(\mathbf{r}) \qquad (56)\\
&= \frac{1}{\ell L_2 \mathcal{N}} \sum_{s,s'\in\mathbb{Z}} e^{2\pi i(k_1 s - k'_1 s')} \int d^2\mathbf{r} (e^{2\pi i k'_2 \frac{y}{L_2}} \varphi_{m'}(x - k'_2 \frac{2\pi\ell^2}{L_2}))^* t^{-s'}_{L_1} e^{i\mathbf{g}\cdot\mathbf{r}} t^{s}_{L_1} (e^{2\pi i k_2 \frac{y}{L_2}} \varphi_m(x - k_2 \frac{2\pi\ell^2}{L_2}))\\
&= \delta_{k_1,k'_1} \frac{s_{tot}}{\ell L_2 \mathcal{N}} \sum_{s\in\mathbb{Z}} e^{2\pi i s k_1} \int d^2\mathbf{r} (e^{2\pi i k'_2 \frac{y}{L_2}} \varphi_{m'}(x - k'_2 \frac{2\pi\ell^2}{L_2}))^* e^{i\mathbf{g}\cdot\mathbf{r}} t^{s}_{L_1} (e^{2\pi i k_2 \frac{y}{L_2}} \varphi_m(x - k_2 \frac{2\pi\ell^2}{L_2}))\\
&= \delta_{k_1,k'_1} \sum_{s\in\mathbb{Z}} \delta_{k_2+\frac{s}{q}, k'_2 - \frac{L_2 g_y}{2\pi}} e^{2\pi i s(k_1 - \frac{k_2}{2})} e^{-i\pi s(s-1)\frac{1}{2q}} \frac{1}{\ell} \int dx \varphi_{m'}(x - k'_2 \frac{2\pi\ell^2}{L_2}) e^{i g_x x} \varphi_m(x - (k_2 + \frac{s}{q})\frac{2\pi\ell^2}{L_2})
\end{aligned}$$

The delta function constraint implies

$$\delta_{k_2+\frac{s}{q}, k'_2 - \frac{L_2 g_y}{2\pi}} = \delta_{k_2,k'_2} \delta_{s,-q g_2}, \qquad (57)$$

where $g_2 = g_y \frac{L_2}{2\pi} \in \mathbb{Z}$. Note that getting $\delta_{k_{1(2)},k_{1'(2')}}$ from the overlap is a direct consequence of Eqs.(37)-(38) for $\frac{1}{q}$ sequence. The above integral can be evaluated as

$$
\begin{aligned}
&= \frac{1}{\ell}\int dx\varphi_{m'}(x-k_2\frac{2\pi\ell^2}{L_2})e^{ig_x x}\varphi_m(x-(k_2-g_2)\frac{2\pi\ell^2}{L_2}) \\
&= \frac{1}{\ell}e^{ig_xk_2\frac{2\pi\ell^2}{L_2}}\int dx\varphi_{m'}(x)e^{ig_x x}\varphi_m(x+g_2\frac{2\pi\ell^2}{L_2}) \\
&= \frac{1}{\ell}e^{ig_xk_2\frac{2\pi\ell^2}{L_2}}\int dx\varphi_{m'}(x)e^{ig_x x}e^{ig_y\ell^2 p_x}\varphi_m(x) \\
&= \frac{1}{\ell}e^{ig_xk_2\frac{2\pi\ell^2}{L_2}}e^{-\frac{i}{2}g_xg_y\ell^2}\int dx\varphi_{m'}(x)e^{c_+a^\dagger+c_-a}\varphi_m(x) \\
&= e^{ig_xk_2\frac{2\pi\ell^2}{L_2}}e^{-\frac{i}{2}g_xg_y\ell^2}\langle m'|\, e^{c_+a^\dagger+c_-a}\,|m\rangle\,,
\end{aligned} \tag{58}
$$

where p_x is momentum operator along x direction later changed to harmonic oscillator(h.o.) basis: $x=\frac{\ell}{\sqrt{2}}(a+a^\dagger)$, and $p_x=\frac{i}{\sqrt{2}\ell}(a^\dagger-a)$. The h.o. kets are defined as $\langle m\mid x\rangle=\frac{1}{\sqrt{\ell}}\varphi_m(x)$, such that $\langle m\mid n\rangle=\delta_{m,n}$. Eventually we use the BCH formula, $e^Xe^Y=e^{X+Y+\frac{1}{2}[X,Y]+\dots}$, and

$$
c_\pm=\frac{i\ell}{\sqrt{2}}(g_x\pm ig_y). \tag{59}
$$

We also know the identity

$$
\langle m'|\, e^{c_+a^\dagger+c_-a}\,|m\rangle=\begin{cases} e^{\frac{1}{2}c_+c_-}\sqrt{\frac{m!}{n!}}(c_+)^{n-m}L_m^{n-m}(-c_+c_-) & \text{for}\quad n\ge m,\\ e^{\frac{1}{2}c_+c_-}\sqrt{\frac{n!}{m!}}(c_-)^{m-n}L_n^{m-n}(-c_+c_-) & \text{for}\quad n<m,\end{cases} \tag{60}
$$

where $L_n^{m-n}(x)$ is the associated Laguerre polynomial,

$$
L_N^m(x)=\sum_{k=0}^{N}\frac{(N+m)!}{(N-k)!(m+k)!k!}(-x)^k. \tag{61}
$$

As shown in Eq.(60), the overlaps are exponentially small in (c_+c_-), i.e. $(g\ell)^2$, and thus negligible. However, beyond an upper-bound on m, the orthonormality begins to fail. Supplementary Fig.(1-a)-(1-h) show that the diagonal values of overlap matrix in Eq.(55) start deviating from 1 beyond some upper-bound on m exactly when the Frobenius norm of $O-\mathbb{I}$, with $\mathbb{I}$ being identity matrix of dimension same as that of O, starts deviating from 0. Thus the deviation of diagonal values from 1 is a good indicative of the breaking up of orthonormality. In this work we choose $m_{max}=\lceil\frac{q-3}{2}\rceil$, which is well below the upper-bound as shown in Supplementary Fig.(1-i).

Supplementary note 4. COMPUTATION OF MATRIX ELEMENTS

A. c-c Coupling

The $c-c$ coupling is given as

$$
H_{cc}^\tau=\sum_{k\in[0,1)\otimes[0,\frac{1}{q})}\sum_{a,a'=1}^{4}\sum_{m=0}^{m_{a,\tau}}\sum_{m'=0}^{m_{a',\tau}}\sum_{r,\tilde r=0}^{p-1}\tilde h^\tau_{[amr],[a'm'\tilde r]}(k)c^\dagger_{a\tau krm}c_{a'\tau k\tilde r m'}, \tag{62}
$$

where

$$
\tilde h^\tau_{[amr],[a'm'r']}(k_1,k_2)=\int d^2\mathbf{r}\,\Psi^*_{a\tau}(\mathbf{r})\chi^*_{krm}(\mathbf{r})H^\tau_{BM}\left(p_x,p_y-\frac{eB}{c}x\right)\Psi_{a'\tau}(\mathbf{r})\chi_{kr'm'}(\mathbf{r}). \tag{63}
$$

Since the BM Hamiltonian is linear in $\mathbf{p}-\frac{e}{c}\mathbf{A}$, we can write it as

$$
H^\tau_{BM}\left(\mathbf{p}-\frac{e}{c}\mathbf{A}\right)=\mathcal{M}^\tau_\mu\left(p_\mu-\frac{e}{c}A_\mu(\mathbf{r})\right)+\mathcal{T}^\tau(\mathbf{r}) \tag{64}
$$

where $\mathcal{M}^{+1}_\mu=v_F\mathbb{I}_2\otimes\sigma$, $\mathcal{M}^{-1}_\mu=-v_F\mathbb{I}_2\otimes\bar\sigma$ with $\sigma=(\sigma_x,\sigma_y)$ and $\bar\sigma=(\sigma_x,-\sigma_y)$. $\mathcal{T}^\tau$ denotes the remaining factors in BM Hamiltonian. So,

$$
H^\tau_{BM}\left(\mathbf{p}-\frac{e}{c}\mathbf{A}\right)\Psi_{a'\tau}(\mathbf{r})\chi_{kr'm'}(\mathbf{r})=\mathcal{M}^\tau_\mu\left(p_\mu-\frac{e}{c}A_\mu(\mathbf{r})\right)\Psi_{a'}(\mathbf{r})\chi_{k_1,k_2+\frac{r'}{q},m'}(\mathbf{r})+\mathcal{T}^\tau(\mathbf{r})\Psi_{a'\tau}(\mathbf{r})\chi_{kr'm'}(\mathbf{r}) \tag{65}
$$

$$
=\left(\mathcal{M}^\tau_\mu p_\mu\Psi_{a'\tau}(\mathbf{r})+\mathcal{T}^\tau(\mathbf{r})\Psi_{a'\tau}(\mathbf{r})\right)\chi_{kr'm'}(\mathbf{r})+\mathcal{M}^\tau_\mu\Psi_{a'\tau}(\mathbf{r})\left(\left(p_\mu-\frac{e}{c}A_\mu(\mathbf{r})\right)\chi_{kr'm'}(\mathbf{r})\right) \tag{66}
$$

$$
=\varepsilon^\tau_{a''a'}\Psi_{a''\tau}(\mathbf{r})\chi_{kr'm'}(\mathbf{r})+\mathcal{M}^\tau_\mu\Psi_{a'\tau}(\mathbf{r})\left(p_\mu-\frac{e}{c}A_\mu(\mathbf{r})\right)\chi_{krm'}(\mathbf{r}), \tag{67}
$$

where the matrix ε^τ is given in Eq.(76) is $\mathbf{k}$ independent because $\mathit{\Psi}_{a\tau}$ is defined at Γ. So,

$$\tilde{h}^\tau_{[amr],[a'm'r']}(k_1,k_2) = \int d^2\mathbf{r}\,\mathit{\Psi}^*_{a\tau}(\mathbf{r})\varepsilon^\tau_{a''a'}\,\mathit{\Psi}_{a''\tau}(\mathbf{r})\chi^*_{krm}(\mathbf{r})\chi_{kr'm'}(\mathbf{r}) \tag{68}$$

$$+\int d^2\mathbf{r}\,\mathit{\Psi}^*_{a\tau}(\mathbf{r})\mathcal{M}^\tau_\mu\,\mathit{\Psi}_{a'\tau}(\mathbf{r})\chi^*_{krm}(\mathbf{r})\left(p_\mu - \frac{e}{c}A_\mu(\mathbf{r})\right)\chi_{kr'm'}(\mathbf{r}) \tag{69}$$

Since the factors $\varepsilon^\tau_{a''a'}\,\mathit{\Psi}^*_{a\tau}(\mathbf{r})\,\mathit{\Psi}_{a''\tau}(\mathbf{r})$ and $\mathit{\Psi}^*_{a\tau}(\mathbf{r})\mathcal{M}^\tau_\mu\,\mathit{\Psi}_{a'\tau}(\mathbf{r})$ are periodic with respect to primitive moiré lattice vectors $\mathbf{L}_1$ and $\mathbf{L}_2$, we can perform a Fourier expansion as

$$\varepsilon^\tau_{a''a'}\,\mathit{\Psi}^*_{a\tau}(\mathbf{r})\,\mathit{\Psi}_{a''\tau}(\mathbf{r}) = \varepsilon^\tau_{a''a'}\sum_{\mathbf{g}} e^{i\mathbf{g}\cdot\mathbf{r}}\left(\frac{1}{A_{tot}}\int d^2\mathbf{r}' e^{-i\mathbf{g}\cdot\mathbf{r}'}\,\mathit{\Psi}^*_{a\tau}(\mathbf{r}')\,\mathit{\Psi}_{a''\tau}(\mathbf{r}')\right) \tag{70}$$

$$\mathit{\Psi}^*_{a\tau}(\mathbf{r})\mathcal{M}^\tau_\mu\,\mathit{\Psi}_{a'\tau}(\mathbf{r}) = \sum_{\mathbf{g}} e^{i\mathbf{g}\cdot\mathbf{r}}\left(\frac{1}{A_{tot}}\int d^2\mathbf{r}' e^{-i\mathbf{g}\cdot\mathbf{r}'}\,\mathit{\Psi}^*_{a\tau}(\mathbf{r}')\mathcal{M}^\tau_\mu\,\mathit{\Psi}_{a'\tau}(\mathbf{r}')\right) \tag{71}$$

As shown in previous section, at small B the overlaps of the MTG LL with $e^{i\mathbf{g}\cdot\mathbf{r}}$ are suppressed by a factor of $\exp\left(-\mathbf{g}^2\ell^2/4\right)$. So at small enough B (large enough ℓ) the overlaps with non-zero $\mathbf{g}$ components are negligible. Keeping only the $\mathbf{g}=0$ Fourier component then gives

$$\begin{aligned}\tilde{h^\tau}_{[amr],[a'm'r']}(k_1,k_2) &\approx \left(\frac{1}{A_{tot}}\int d^2\mathbf{r}\,\mathit{\Psi}^*_{a\tau}(\mathbf{r})\varepsilon^\tau_{a''a'}\,\mathit{\Psi}_{a''\tau}(\mathbf{r})\right)\left(\int d^2\mathbf{r}\chi^*_{km}(\mathbf{r})\chi_{kr'm'}(\mathbf{r})\right) + \\ &\left(\frac{1}{A_{tot}}\int d^2\mathbf{r}\,\mathit{\Psi}^*_{a\tau}(\mathbf{r})\mathcal{M}^\tau{}_\mu\,\mathit{\Psi}_{a'\tau}(\mathbf{r})\right)\left(\int d^2\mathbf{r}\chi^*_{krm}(\mathbf{r})\left(p_\mu - \frac{e}{c}A_\mu(\mathbf{r})\right)\chi_{kr'm'}(\mathbf{r})\right) + \mathcal{O}\left(\exp\left(-\mathbf{g}^2\ell^2/4\right)\right) \quad (72)\\ &= \varepsilon^\tau_{aa'}\delta_{rr'}\delta_{mm'} + \mathcal{M}^{\tau,\mu}_{aa'}\left(\int d^2\mathbf{r}\chi^*_{krm}(\mathbf{r})\left(p_\mu - \frac{e}{c}A_\mu(\mathbf{r})\right)\chi_{kr'm'}(\mathbf{r})\right) + \mathcal{O}\left(\exp\left(-\mathbf{g}^2\ell^2/4\right)\right), \quad (73)\end{aligned}$$

where

$$\mathcal{M}^{\tau,\mu}_{aa'} = \frac{1}{A_{tot}}\int d^2\mathbf{r}\,\mathit{\Psi}^*_{a\tau}(\mathbf{r})\mathcal{M}^\tau_\mu\,\mathit{\Psi}_{a'\tau}(\mathbf{r}) \tag{74}$$

The matrices $\epsilon^\tau_{a,a'}$ and $\mathcal{M}^{\tau,\mu}_{aa'}$ can be obtained from the zero field cc coupling matrix

$$H^{c,\tau}_{aa'}(\mathbf{k}) = \begin{pmatrix} 0_{2\times 2} & v_*(\tau k_x\sigma_0 + ik_y\sigma_z) \\ v_*(\tau k_x\sigma_0 - ik_y\sigma_z) & M\sigma_x\end{pmatrix} \tag{75}$$

as

$$M^{\tau,x}_{aa'} = \frac{\tau v_*}{\hbar}\begin{pmatrix} 0 & \sigma_0 \\ \sigma_0 & 0\end{pmatrix}_{aa'},\quad M^{\tau,y}_{aa'} = \frac{iv_*}{\hbar}\begin{pmatrix} 0 & \sigma_z \\ -\sigma_z & 0\end{pmatrix}_{aa'},\quad \varepsilon^\tau_{aa'} = M\begin{pmatrix} 0 & 0 \\ 0 & \sigma_x\end{pmatrix}_{aa'}. \tag{76}$$

where the Pauli matrices σ act in the orbital space of c fermions. Since $\left[\hat{t}_{\mathbf{L}_{1,2}}, p_\mu - \frac{e}{c}A_\mu(\mathbf{r})\right] = 0$, we have

$$\left(p_\mu - \frac{e}{c}A_\mu(\mathbf{r})\right)\chi_{kr'm'}(\mathbf{r}) = \frac{1}{\sqrt{\ell L_2 \mathcal{N}}}\sum_{s\in\mathbb{Z}} e^{2\pi i s k_1}\left(\hat{t}^s_{\mathbf{L}_1}\left(p_\mu - \frac{e}{c}A_\mu(\mathbf{r})\right)e^{2\pi i\left(k_2+\frac{r'}{q}\right)\frac{y}{L_2}}\varphi_{m'}\left(x - \left(k_2 + \frac{r'}{q}\right)\frac{2\pi\ell^2}{L_2}\right)\right). \tag{77}$$

The action of the two components of $p_\mu - \frac{e}{c}A_\mu(\mathbf{r})$ in Landau gauge can be calculated as

$$\begin{aligned}&\frac{\hbar}{i}\frac{\partial}{\partial x}e^{2\pi i\left(k_2+\frac{r'}{q}\right)\frac{y}{L_2}}\varphi_{m'}\left(x - \left(k_2+\frac{r'}{q}\right)\frac{2\pi\ell^2}{L_2}\right) = \frac{\hbar}{i}\frac{1}{\ell}e^{2\pi i\left(k_2+\frac{r'}{q}\right)\frac{y}{L_2}}\ell\frac{\partial}{\partial x}\varphi_{m'}\left(x - \left(k_2+\frac{r'}{q}\right)\frac{2\pi\ell^2}{L_2}\right)\\ &= \frac{\hbar}{i}\frac{1}{\ell}e^{2\pi i\left(k_2+\frac{r'}{q}\right)\frac{y}{L_2}}\frac{\hat{a}-\hat{a}^\dagger}{\sqrt{2}}\varphi_{m'}\left(x - \left(k_2+\frac{r'}{q}\right)\frac{2\pi\ell^2}{L_2}\right)\\ &= \frac{\hbar}{i}\frac{1}{\sqrt{2}\ell}e^{2\pi i\left(k_2+\frac{r'}{q}\right)\frac{y}{L_2}}\left(\sqrt{m'}\varphi_{m'-1}\left(x - \left(k_2+\frac{r'}{q}\right)\frac{2\pi\ell^2}{L_2}\right) - \sqrt{m'+1}\varphi_{m'+1}\left(x - \left(k_2+\frac{r'}{q}\right)\frac{2\pi\ell^2}{L_2}\right)\right)\end{aligned} \tag{78}$$

and

$$\left(\frac{\hbar}{i}\frac{\partial}{\partial y}-\frac{eB}{c}x\right)e^{2\pi i\left(k_2+\frac{r'}{q}\right)\frac{y}{L_2}}\varphi_{m'}\left(x-\left(k_2+\frac{r'}{q}\right)\frac{2\pi\ell^2}{L_2}\right)=$$

$$\frac{\hbar}{\ell}\left(\left(k_2+\frac{r'}{q}\right)\frac{2\pi\ell}{L_2}-\frac{x}{\ell}\right)e^{2\pi i\left(k_2+\frac{r'}{q}\right)\frac{y}{L_2}}\varphi_{m'}\left(x-\left(k_2+\frac{r'}{q}\right)\frac{2\pi\ell^2}{L_2}\right)= \tag{79}$$

$$-\frac{\hbar}{\ell}e^{2\pi i\left(k_2+\frac{r'}{q}\right)\frac{y}{L_2}}\frac{a+a^\dagger}{\sqrt{2}}\varphi_{m'}\left(x-\left(k_2+\frac{r'}{q}\right)\frac{2\pi\ell^2}{L_2}\right)= \tag{80}$$

$$-\frac{\hbar}{\sqrt{2}\ell}e^{2\pi i\left(k_2+\frac{r'}{q}\right)\frac{y}{L_2}}\left(\sqrt{m'}\varphi_{m'-1}\left(x-\left(k_2+\frac{r'}{q}\right)\frac{2\pi\ell^2}{L_2}\right)+\sqrt{m'+1}\varphi_{m'+1}\left(x-\left(k_2+\frac{r'}{q}\right)\frac{2\pi\ell^2}{L_2}\right)\right). \tag{81}$$

Substituting back to χ, and using the orthogonality of χ's and Eq.(76), we finally have,

$$\tilde{h}^{+1}_{[amr],[a'm'\bar{r}]}(k_1,k_2)\approx\delta_{r\bar{r}}\begin{pmatrix}0 & 0 & -i\frac{\sqrt{2}v_*}{\ell}\sqrt{m'}\delta_{m+1,m'} & 0\\ 0 & 0 & 0 & i\frac{\sqrt{2}v_*}{\ell}\sqrt{m}\delta_{m,m'+1}\\ i\frac{\sqrt{2}v_*}{\ell}\sqrt{m}\delta_{m,m'+1} & 0 & 0 & M\delta_{mm'}\\ 0 & -i\frac{\sqrt{2}v_*}{\ell}\sqrt{m'}\delta_{m+1,m'} & M\delta_{mm'} & 0\end{pmatrix}_{aa'} \tag{82}$$

$$\tilde{h}^{-1}_{[amr],[a'm'\bar{r}]}(k_1,k_2)\approx\delta_{r\bar{r}}\begin{pmatrix}0 & 0 & -i\frac{\sqrt{2}v_*}{\ell}\sqrt{m}\delta_{m,m'+1} & 0\\ 0 & 0 & 0 & i\frac{\sqrt{2}v_*}{\ell}\sqrt{m'}\delta_{m+1,m'}\\ i\frac{\sqrt{2}v_*}{\ell}\sqrt{m'}\delta_{m+1,m'} & 0 & 0 & M\delta_{mm'}\\ 0 & -i\frac{\sqrt{2}v_*}{\ell}\sqrt{m}\delta_{m,m'+1} & M\delta_{mm'} & 0\end{pmatrix}_{aa'}. \tag{83}$$

Note that a straightforward canonical substitution in Eq.(75), $k_x+ik_y\to\sqrt{2B}\hat{a}$, would yield us the same finite field $c-c$ coupling, where $\hat{a}$ denotes the LL lowering operator for the corresponding c fermion LLs.

B. c-f Coupling

The $c-f$ coupling at finite $\mathbf{B}$ is given as

$$H^\tau_{cf}=\sum_{k\in[0,1)\otimes[0,\frac{1}{q})}\sum_{a=1}^{4}\sum_{b=1}^{2}\sum_{m=0}^{m_{a,\tau}}\sum_{r=0}^{p-1}\sum_{r'=0}^{q-1}h^\tau_{[amr],[br']}(k)c^\dagger_{a\tau krm}f_{b\tau kr'}, \tag{84}$$

where

$$h^\tau_{[amr],[br']}(k_1,k_2)=\int d^2\mathbf{r}\,\Psi^*_{a\tau}(\mathbf{r})\chi^*_{krm}(\mathbf{r})H^\tau_{BM}\left(p_x,p_y-\frac{eB}{c}x\right)\eta_{b\tau kr'}(\mathbf{r}) \tag{85}$$

$$=\frac{1}{\sqrt{\mathcal{N}}}\sum_{s\in\mathbb{Z}}e^{2\pi isk_1}\int d^2\mathbf{r}\,\Psi^*_{a\tau}(\mathbf{r})\chi^*_{k_1,k_2+\frac{r}{q},m}(\mathbf{r})H^\tau_{BM}\left(p_x,p_y-\frac{eB}{c}x\right)\hat{t}^s_{\mathbf{L}_1}w_{b\tau}(\mathbf{r},\left(k_2+\frac{r'}{q}\right)\mathbf{g}_2) \tag{86}$$

$$=\frac{1}{\sqrt{\mathcal{N}}}\sum_{s\in\mathbb{Z}}e^{2\pi isk_1}\int d^2\mathbf{r}\,\Psi^*_{a\tau}(\mathbf{r})\chi^*_{krm}(\mathbf{r})\hat{t}^s_{\mathbf{L}_1}H^\tau_{BM}\left(p_x,p_y-\frac{eB}{c}x\right)w_{b\tau}(\mathbf{r},\left(k_2+\frac{r'}{q}\right)\mathbf{g}_2) \tag{87}$$

$$=\frac{s_{tot}}{\sqrt{\mathcal{N}}}\int d^2\mathbf{r}\,\Psi^*_{a\tau}(\mathbf{r})\chi^*_{krm}(\mathbf{r})H^\tau_{BM}\left(p_x,p_y-\frac{eB}{c}x\right)w_{b\tau}\left(\mathbf{r},\left(k_2+\frac{r'}{q}\right)\mathbf{g}_2\right) \tag{88}$$

$$=\frac{s_{tot}}{\sqrt{\mathcal{N}}}\sum_{n\in\mathbb{Z}}e^{2\pi i\left(k_2+\frac{r'}{q}\right)n}\int d^2\mathbf{r}\,\Psi^*_{a\tau}(\mathbf{r})\chi^*_{krm}(\mathbf{r})H^\tau_{BM}\left(p_x,p_y-\frac{eB}{c}x\right)\hat{t}^n_{\mathbf{L}_2}W_{\mathbf{0},b\tau}(\mathbf{r}) \tag{89}$$

$$=\frac{s_{tot}}{\sqrt{\mathcal{N}}}\sum_{n\in\mathbb{Z}}e^{2\pi i\left(k_2+\frac{r'}{q}\right)n}\int d^2\mathbf{r}\,\Psi^*_{a\tau}(\mathbf{r})\left(\hat{t}^{-n}_{\mathbf{L}_2}\chi_{krm}(\mathbf{r})\right)^*H^\tau_{BM}\left(p_x,p_y-\frac{eB}{c}x\right)W_{\mathbf{0},b\tau}(\mathbf{r}) \tag{90}$$

$$=\frac{s_{tot}}{\sqrt{\mathcal{N}}}\sum_{n\in\mathbb{Z}}e^{2\pi i\left(k_2+\frac{r'}{q}\right)n}\int d^2\mathbf{r}\,\Psi^*_{a\tau}(\mathbf{r})\left(\hat{t}^{-n}_{\mathbf{L}_2}\chi_{krm}(\mathbf{r})\right)^*H^\tau_{BM}\left(p_x,p_y\right)W_{\mathbf{0},b\tau}(\mathbf{r})$$

$$-\frac{s_{tot}}{\sqrt{\mathcal{N}}}\sum_{n\in\mathbb{Z}}e^{2\pi i\left(k_2+\frac{r'}{q}\right)n}\int d^2\mathbf{r}\,\Psi^*_{a\tau}(\mathbf{r})\left(\hat{t}^{-n}_{\mathbf{L}_2}\chi_{krm}(\mathbf{r})\right)^*\left(\frac{eBx}{c}\mathcal{M}^\tau_y\right)W_{\mathbf{0},b\tau}(\mathbf{r}) \tag{91}$$

Note that the B-field term eventually turns out negligible because it acts on a well localized function and so at small B it is exponentially suppressed in the region where the vector potential is appreciable.

Using the fact $\hat{t}^{-1}_{\mathbf{L}_2}\hat{t}_{\mathbf{L}_1} = e^{2\pi i\frac{p}{q}}\hat{t}_{\mathbf{L}_1}\hat{t}^{-1}_{\mathbf{L}_2}$, we have

$$\hat{t}^{-n}_{\mathbf{L}_2}\chi_{krm}(\mathbf{r}) = \frac{1}{\sqrt{\ell L_2 \mathcal{N}}}\sum_{s\in\mathbb{Z}} e^{2\pi i s k_1}\hat{t}^{-n}_{\mathbf{L}_2}\hat{t}^{s}_{\mathbf{L}_1} e^{2\pi i\left(k_2+\frac{r}{q}\right)\frac{y}{L_2}}\varphi_m\left(x-\left(k_2+\frac{r}{q}\right)\frac{2\pi\ell^2}{L_2}\right) \tag{92}$$

$$= e^{2\pi i\left(k_2+\frac{r}{q}\right)n}\frac{1}{\sqrt{\ell L_2\mathcal{N}}}\sum_{s\in\mathbb{Z}} e^{2\pi i s k_1}e^{2\pi i s n\frac{p}{q}}\hat{t}^{s}_{\mathbf{L}_1}e^{2\pi i\left(k_2+\frac{r}{q}\right)\frac{y}{L_2}}\varphi_m\left(x-\left(k_2+\frac{r}{q}\right)\frac{2\pi\ell^2}{L_2}\right) \tag{93}$$

$$= e^{2\pi i\left(k_2+\frac{r}{q}\right)n}\chi_{\mathrm{mod}\left(k_1+n\frac{p}{q},1\right)k_2 r m}(\mathbf{r}) \tag{94}$$

Therefore,

$$h^{\tau}_{[amr],[br']}(k_1,k_2) = \frac{s_{tot}}{\sqrt{\mathcal{N}}}\sum_{n\in\mathbb{Z}} e^{2\pi i n\frac{r'-r}{q}}\int d^2\mathbf{r}\,\chi^*_{\mathrm{mod}\left(k_1+n\frac{p}{q},1\right),k_2 r m}(\mathbf{r})\,\Psi^*_{a\tau}(\mathbf{r})H\left(p_x,p_y\right)W_{\mathbf{0},b\tau}(\mathbf{r}) \tag{95}$$

$$-\left(\frac{eB}{c}\right)\frac{s_{tot}}{\sqrt{\mathcal{N}}}\sum_{n\in\mathbb{Z}} e^{2\pi i n\frac{r'-r}{q}}\int d^2\mathbf{r}\left(x\chi^*_{\mathrm{mod}\left(k_1+n\frac{p}{q},1\right),k_2 r m}(\mathbf{r})\right)\Psi^*_{a\tau}(\mathbf{r})\mathcal{M}^{\tau}_{y}W_{\mathbf{0},b\tau}(\mathbf{r}) \tag{96}$$

In order to proceed we use the fact that [2]

$$\int d^2\mathbf{r}e^{-i\mathbf{k}'\cdot\mathbf{r}}\,\Psi^*_{a\tau}(\mathbf{r})H^{\tau}_{BM}\left(p_x,p_y\right)W_{\mathbf{0}b\tau}(\mathbf{r}) \approx \sqrt{A_{uc}}e^{-\mathbf{k}'^2\lambda^2/2}H^{cf,\tau}_{ab}(\mathbf{k}'), \tag{97}$$

which is $\sqrt{NA_{uc}}$ bigger than in [2] because of choice of normalisation of χ discussed in previous section. Using

$$H^{cf,\tau}_{ab}(\mathbf{k}') = \begin{pmatrix}\gamma\sigma_0 + v'_*(\eta_v k_x\sigma_x + k_y\sigma_y)\\ 0_{2\times 2}\end{pmatrix}_{ab}, \tag{98}$$

we have

$$\Psi^*_{a\tau}(\mathbf{r})H^{\tau}_{BM}\left(p_x,p_y\right)W_{\mathbf{0},b\tau}(\mathbf{r}) = \int\frac{d^2\mathbf{k}'}{(2\pi)^2}e^{i\mathbf{k}'\cdot\mathbf{r}}\int d^2\mathbf{r}'e^{-i\mathbf{k}'\cdot\mathbf{r}'}\,\Psi^*_{a\tau}(\mathbf{r}')H^{\tau}_{BM}\left(p'_x,p'_y\right)W_{\mathbf{0},b\tau}(\mathbf{r}') \tag{99}$$

$$\approx \sqrt{A_{uc}}\int\frac{d^2\mathbf{k}}{(2\pi)^2}e^{i\mathbf{k}\cdot\mathbf{r}}e^{-\mathbf{k}^2\lambda^2/2}H^{cf,\tau}_{ab}(\mathbf{k}) \tag{100}$$

$$= \sqrt{A_{uc}}H^{cf,\tau}_{ab}\left(\frac{1}{i}\frac{\partial}{\partial\mathbf{r}}\right)\int\frac{d^2\mathbf{k}}{(2\pi)^2}e^{i\mathbf{k}\cdot\mathbf{r}}e^{-\mathbf{k}^2\lambda^2/2} \tag{101}$$

$$= \sqrt{A_{uc}}H^{cf,\tau}_{ab}\left(\frac{1}{i}\frac{\partial}{\partial\mathbf{r}}\right)\frac{1}{2\pi\lambda^2}e^{-\mathbf{r}^2/(2\lambda^2)}. \tag{102}$$

Moreover since $H^{\tau}_{BM}(\mathbf{p})$ is linear in $\mathbf{p}$, we can approximate

$$\int d^2\mathbf{r}e^{-i\mathbf{k}'\cdot\mathbf{r}}\,\Psi^*_{a\tau}(\mathbf{r})\mathcal{M}^{\tau}_{y}W_{\mathbf{0},b\tau}(\mathbf{r}) \approx \sqrt{A_{uc}}e^{-\mathbf{k}'^2\lambda^2/2}M^{\tau}_{ab}, \tag{103}$$

where M^{τ}_{ab} can be read of from Eq.(98) to be

$$M^{\tau}_{ab} = -\frac{iv'_*}{\hbar}\begin{pmatrix}0 & 1\\ -1 & 0\\ 0 & 0\\ 0 & 0\end{pmatrix}_{(ab)} = M_{ab}. \tag{104}$$

Thus we have

$$\Psi^*_{a\tau}(\mathbf{r})\mathcal{M}^{\tau}_{y}W_{\mathbf{0},b\tau}(\mathbf{r}) = \int\frac{d^2\mathbf{k}'}{(2\pi)^2}e^{-i\mathbf{k}'\cdot\mathbf{r}}\int d^2\mathbf{r}'e^{-i\mathbf{k}'\cdot\mathbf{r}'}\,\Psi^*_{a\tau}(\mathbf{r}')\mathcal{M}^{\tau}_{y}W_{\mathbf{0},b\tau}(\mathbf{r}') \tag{105}$$

$$\approx \sqrt{A_{uc}}\int\frac{d^2\mathbf{k}'}{(2\pi)^2}e^{-i\mathbf{k}'\cdot\mathbf{r}}e^{-\frac{\mathbf{k}'^2\lambda^2}{2}}M_{ab} \tag{106}$$

$$= \sqrt{A_{uc}}e^{-\mathbf{r}^2/(2\lambda^2)}\frac{1}{2\pi\lambda^2}M_{ab}. \tag{107}$$

Therefore,

$$h^{\tau}_{[amr],[br']}(k_1,k_2) \approx \frac{s_{tot}\sqrt{A_{uc}}}{\sqrt{\mathcal{N}}}\sum_{n\in\mathbb{Z}} e^{2\pi i n\frac{r'-r}{q}} \int d^2\mathbf{r}\chi^*_{\text{mod}\left(k_1+n\frac{p}{q},1\right)k_2rm}(\mathbf{r}) H^{cf,\tau}_{ab}\left(\frac{1}{i}\frac{\partial}{\partial\mathbf{r}}\right)\frac{1}{2\pi\lambda^2}e^{-\mathbf{r}^2/(2\lambda^2)}$$
$$-\frac{eB}{c}\frac{s_{tot}\sqrt{A_{uc}}}{\sqrt{\mathcal{N}}}\sum_{n\in\mathbb{Z}} e^{2\pi i n\frac{r'-r}{q}} \int d^2\mathbf{r}x\chi^*_{\text{mod}\left(k_1+n\frac{p}{q},1\right)k_2rm}(\mathbf{r}) M_{ab}\frac{1}{2\pi\lambda^2}e^{-\mathbf{r}^2/(2\lambda^2)} \tag{108}$$
$$= \frac{s_{tot}\sqrt{A_{uc}}}{\sqrt{\mathcal{N}}}\sum_{n\in\mathbb{Z}} e^{2\pi i n\frac{r'-r}{q}} \int d^2\mathbf{r}\left(H^{cf,\tau}_{ab}\left(\frac{1}{i}\frac{\partial}{\partial\mathbf{r}}\right)\chi_{\text{mod}\left(k_1+n\frac{p}{q},1\right)k_2rm}(\mathbf{r})\right)^*\frac{1}{2\pi\lambda^2}e^{-\mathbf{r}^2/(2\lambda^2)}$$
$$-\frac{eB}{c}\frac{s_{tot}\sqrt{A_{uc}}}{\sqrt{\mathcal{N}}}\sum_{n\in\mathbb{Z}} e^{2\pi i n\frac{r'-r}{q}} \int d^2\mathbf{r}\left(x\chi_{\text{mod}\left(k_1+n\frac{p}{q},1\right)k_2rm}(\mathbf{r})\right)^*\frac{1}{2\pi\lambda^2}e^{-\mathbf{r}^2/(2\lambda^2)}M_{ab}. \tag{109}$$

Using $\hat{t}^s_{\mathbf{L}_1} = e^{-\pi i s(s-1)\frac{p}{q}\frac{L_{1y}}{L_2}}e^{2\pi i s\frac{p}{q}\frac{y}{L_2}}\hat{T}^s_{\mathbf{L}_1}$, derived in the Supplementary note 2 A, we have

$$\chi_{\text{mod}\left(k_1+n\frac{p}{q},1\right),k_2rm}(\mathbf{r}) = \frac{1}{\sqrt{\ell L_2\mathcal{N}}}\sum_{s\in\mathbb{Z}} e^{2\pi i s n\frac{p}{q}}e^{2\pi i s k_1}\hat{t}^s_{\mathbf{L}_1}e^{2\pi i(k_2+\frac{r}{q})\frac{y}{L_2}}\varphi_m\left(x-(k_2+\frac{r}{q})\frac{2\pi\ell^2}{L_2}\right) \tag{110}$$
$$= \frac{1}{\sqrt{\ell L_2\mathcal{N}}}\sum_{s\in\mathbb{Z}} e^{2\pi i s n\frac{p}{q}}e^{2\pi i s k_1}e^{-\pi i s(s-1)\frac{p}{q}\frac{L_{1y}}{L_2}}e^{2\pi i s\frac{p}{q}\frac{y}{L_2}}\hat{T}^s_{\mathbf{L}_1}e^{2\pi i(k_2+\frac{r}{q})\frac{y}{L_2}}\varphi_m\left(x-(k_2+\frac{r}{q})\frac{2\pi\ell^2}{L_2}\right) \tag{111}$$
$$= \frac{1}{\sqrt{\ell L_2\mathcal{N}}}\sum_{s\in\mathbb{Z}} e^{2\pi i s n\frac{p}{q}}e^{2\pi i s k_1}e^{-\pi i s(s-1)\frac{p}{q}\frac{L_{1y}}{L_2}}e^{2\pi i s\frac{p}{q}\frac{y}{L_2}}e^{2\pi i(k_2+\frac{r}{q})\frac{y-sL_{1y}}{L_2}}\varphi_m\left(x-sL_{1x}-(k_2+\frac{r}{q})\frac{2\pi\ell^2}{L_2}\right) \tag{112}$$
$$= \frac{1}{\sqrt{\ell L_2\mathcal{N}}}\sum_{s\in\mathbb{Z}} e^{2\pi i s n\frac{p}{q}}e^{2\pi i s k_1}e^{-2\pi i s(k_2+\frac{r}{q})\frac{L_{1y}}{L_2}}e^{-\pi i s(s-1)\frac{p}{q}\frac{L_{1y}}{L_2}}e^{2\pi i\left(k_2+\frac{r}{q}+s\frac{p}{q}\right)\frac{y}{L_2}}\varphi_m\left(x-sL_{1x}-(k_2+\frac{r}{q})\frac{2\pi\ell^2}{L_2}\right) \tag{113}$$

Substituting Eq.(113) back in Eq.(109), we have

$$h^{\tau}_{[amr],[br']}(k_1,k_2) \approx \frac{s_{tot}\sqrt{A_{uc}}}{\mathcal{N}\sqrt{\ell L_2}}\sum_{n\in\mathbb{Z}}\sum_{s\in\mathbb{Z}} e^{2\pi i n\frac{r'-r}{q}}e^{-2\pi i s n\frac{p}{q}}e^{-2\pi i s k_1}e^{2\pi i s\left(k_2+\frac{r}{q}\right)\frac{L_{1y}}{L_2}}e^{\pi i s(s-1)\frac{p}{q}\frac{L_{1y}}{L_2}}$$
$$\left[\int d^2\mathbf{r}e^{-2\pi i\left(k_2+\frac{r}{q}+s\frac{p}{q}\right)\frac{y}{L_2}}\varphi_m\left(x-sL_{1x}-\left(k_2+\frac{r}{q}\right)\frac{2\pi\ell^2}{L_2}\right)H^{cf,\tau}_{ab}\left(\frac{1}{i}\frac{\partial}{\partial\mathbf{r}}\right)\frac{1}{2\pi\lambda^2}e^{-\mathbf{r}^2/(2\lambda^2)}\right.$$
$$\left.-\frac{eB}{c}\int d^2\mathbf{r}e^{-2\pi i\left(k_2+\frac{r}{q}+s\frac{p}{q}\right)\frac{y}{L_2}}x\varphi_m\left(x-sL_{1x}-\left(k_2+\frac{r}{q}\right)\frac{2\pi\ell^2}{L_2}\right)M_{ab}\frac{1}{2\pi\lambda^2}e^{-\mathbf{r}^2/(2\lambda^2)}\right] \tag{114}$$
$$\approx \frac{s_{tot}n_{tot}\sqrt{A_{uc}}}{\mathcal{N}\sqrt{\ell L_2}}\sum_{j\in\mathbb{Z}}\sum_{s\in\mathbb{Z}}\delta_{r'-r,jq+sp}e^{-2\pi i s k_1}e^{2\pi i s\left(k_2+\frac{r}{q}\right)\frac{L_{1y}}{L_2}}e^{\pi i s(s-1)\frac{p}{q}\frac{L_{1y}}{L_2}}$$
$$\left[\int d^2\mathbf{r}e^{-2\pi i\left(k_2+\frac{r}{q}+s\frac{p}{q}\right)\frac{y}{L_2}}\varphi_m\left(x-sL_{1x}-\left(k_2+\frac{r}{q}\right)\frac{2\pi\ell^2}{L_2}\right)H^{cf,\tau}_{ab}\left(\frac{1}{i}\frac{\partial}{\partial\mathbf{r}}\right)\frac{1}{2\pi\lambda^2}e^{-\mathbf{r}^2/(2\lambda^2)}\right.$$
$$\left.-\frac{eB}{c}\int d^2\mathbf{r}e^{-2\pi i\left(k_2+\frac{r}{q}+s\frac{p}{q}\right)\frac{y}{L_2}}x\varphi_m\left(x-sL_{1x}-\left(k_2+\frac{r}{q}\right)\frac{2\pi\ell^2}{L_2}\right)M_{ab}\frac{1}{2\pi\lambda^2}e^{-\mathbf{r}^2/(2\lambda^2)}\right] \tag{115}$$

where the sum over n above led to the Diophantine equation

$$\frac{r'-r-sp}{q} = \text{integer} \equiv j. \tag{116}$$

Plugging in the normalization factors results in

$$h^{\tau}_{[amr],[br']}(k_1,k_2) \approx \frac{\sqrt{L_{1x}}}{\sqrt{\ell}}\sum_{j\in\mathbb{Z}}\sum_{s\in\mathbb{Z}}\delta_{r'-r,jq+sp}e^{-2\pi i s k_1}e^{2\pi i s\left(k_2+\frac{r}{q}\right)\frac{L_{1y}}{L_2}}e^{\pi i s(s-1)\frac{p}{q}\frac{L_{1y}}{L_2}}$$
$$\frac{1}{2\pi\lambda^2}\left[\int d^2\mathbf{r}e^{-2\pi i\left(k_2+\frac{r}{q}+s\frac{p}{q}\right)\frac{y}{L_2}}\varphi_m\left(x-sL_{1x}-\left(k_2+\frac{r}{q}\right)\frac{2\pi\ell^2}{L_2}\right)H^{cf,\tau}_{ab}\left(\frac{1}{i}\frac{\partial}{\partial\mathbf{r}}\right)e^{-\mathbf{r}^2/(2\lambda^2)}\right.$$
$$\left.-\frac{\hbar}{\ell^2}\int d^2\mathbf{r}e^{-2\pi i\left(k_2+\frac{r}{q}+s\frac{p}{q}\right)\frac{y}{L_2}}x\varphi_m\left(x-sL_{1x}-\left(k_2+\frac{r}{q}\right)\frac{2\pi\ell^2}{L_2}\right)M_{ab}e^{-\mathbf{r}^2/(2\lambda^2)}\right]. \tag{117}$$

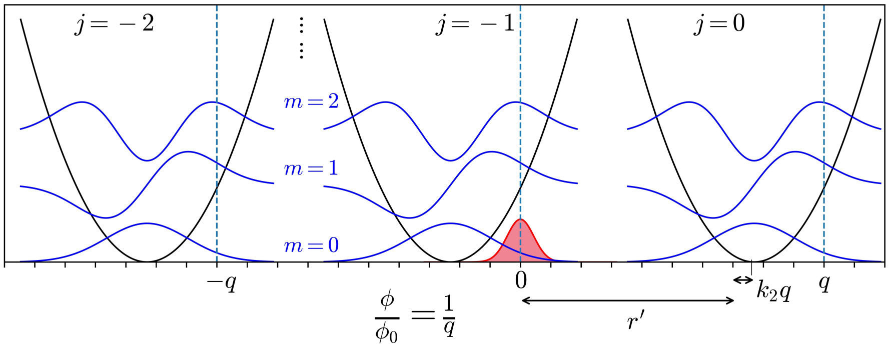

Supplementary Figure 2. Schematic representation of the cf coupling for $\phi/\phi_0 = 1/q$ discussed in the methods section A of main text. Each tick represents (the 1D projection of) a moire unit cell, illustrating the overlap between a 2D localized heavy state with size λ siting at the origin (red) and a Landau level (LL) i.e. a 1D harmonic oscillator (h.o.) shifted in the x-direction, its wavefunctions sketched by blue, with a plane wave phase variation in the y-direction (not shown) that depends on the shift. The r' determines the momentum absorbed by the LL as well as the unit cell to which the h.o. is shifted and k_2q fine tunes the shift within the unit cell. The index j then determines q-unit-cell periodic revival of the h.o. The black parabolas mimic a quadratic potential to accompany the h.o. wavefunctions.

Note that the y integrals are elementary. At small $\mathbf{B}$ and not too large m, the x integrals we be performed by taking the advantage of the mismatch in length scales i.e. $\ell \gg \lambda$ and Taylor expand the harmonic oscillator functions about the origin. However at larger $\mathbf{B}$ we may need to obtain the integrals with higher accuracy. To this end we will find it useful to employ two-variable Hermite polynomial $\mathcal{H}_n(x, y)$ result from [6]. It will be useful to first discuss the integrals required for casting Eq.(117) into a closed form.

We start by defining the two-variable Hermite polynomial as

$$\mathcal{H}_n(x, y) = n! \sum_{k=0}^{[\frac{n}{2}]} \frac{x^{n-2k} y^k}{(n-2k)!k!}, \tag{118}$$

where $[n]$ denotes the floor function at n. The two-variable Hermite polynomials are related to the Hermite polynomial in harmonic oscillator wavefunction as $H_m(x) = \mathcal{H}_m(2x, -1)$. The required x integral in Eq.(117) then reads

$$A_m(\lambda, x_0) = \int_{-\infty}^{\infty} dx e^{-x^2/(2\lambda^2)} \varphi_m(x - x_0) = \frac{1}{\pi^{\frac{1}{4}}} \frac{1}{\sqrt{2^m m!}} \int_{-\infty}^{\infty} dx e^{-x^2/(2\lambda^2)} e^{-(x-x_0)^2/2\ell^2} H_m((x - x_0)/\ell) \tag{119}$$

Let $y = x/\ell$ and $y_0 = x_0/\ell$. Then,

$$A_m(\lambda, \ell y_0) = \frac{\ell}{\pi^{\frac{1}{4}}} \frac{1}{\sqrt{2^m m!}} \int_{-\infty}^{\infty} dy e^{-\frac{\ell^2}{2\lambda^2} y^2} e^{-\frac{1}{2}(y-y_0)^2} H_m(y - y_0) \tag{120}$$

$$= \frac{\ell}{\pi^{\frac{1}{4}}} \frac{e^{-\frac{1}{2}y_0^2}}{\sqrt{2^m m!}} \int_{-\infty}^{\infty} dy e^{-\frac{1}{2}\left(\frac{\ell^2}{\lambda^2}+1\right)y^2} e^{y_0 y} H_m(y - y_0) \tag{121}$$

$$= \frac{\ell}{\pi^{\frac{1}{4}}} \frac{e^{-\frac{1}{2}y_0^2}}{\sqrt{2^m m!}} \int_{-\infty}^{\infty} dy e^{-\frac{1}{2}\left(\frac{\ell^2}{\lambda^2}+1\right)y^2} e^{y_0 y} \mathcal{H}_m(2y - 2y_0, -1). \tag{122}$$

Now, using the formula in [6]

$$\int_{-\infty}^{\infty} dx \mathcal{H}_n(ax + b, y) e^{-cx^2 + \alpha x} = \sqrt{\frac{\pi}{c}} e^{\frac{\alpha^2}{4c}} \mathcal{H}_n\left(b + \frac{\alpha}{2}\frac{a}{c}, y + \frac{a^2}{4c}\right) \tag{123}$$

we finally have

$$A_m(\lambda, x_0) = \frac{\pi^{\frac{1}{4}}}{\sqrt{2^{m-1} m!}} \sqrt{\frac{\lambda^2 \ell^2}{\ell^2 + \lambda^2}} e^{-\frac{1}{2}x_0^2/(\ell^2+\lambda^2)} \mathcal{H}_m\left(-2x_0 \frac{\ell}{\ell^2 + \lambda^2}, \frac{2\lambda^2}{\ell^2 + \lambda^2} - 1\right). \tag{124}$$

Note that

$$A_m(\lambda \to 0, x_0) = \sqrt{2\pi}\lambda \frac{\pi^{-\frac{1}{4}}}{\sqrt{2^m m!}} e^{-\frac{1}{2}x_0^2/\ell^2} \mathcal{H}_m\left(-2\frac{x_0}{\ell}, -1\right) = \sqrt{2\pi}\lambda \varphi_m(-x_0) \tag{125}$$

as expected because in the limit $\lambda \to 0$, the harmonic oscillator wavefunction does not vary significantly within the spatial extent of the heavy fermion gaussian, which is set by λ.

Now, let

$$\mathcal{F}_m(\lambda, x_0) = \frac{1}{\sqrt{2\pi}\lambda} A_m(\lambda, x_0) \tag{126}$$

$$= \frac{1}{\pi^{\frac{1}{4}}\sqrt{2^m m!}} \sqrt{\frac{\ell^2}{\ell^2+\lambda^2}} e^{-\frac{1}{2}x_0^2/(\ell^2+\lambda^2)} \mathcal{H}_m\left(-2x_0\frac{\ell}{\ell^2+\lambda^2}, \frac{2\lambda^2}{\ell^2+\lambda^2} - 1\right) \tag{127}$$

$$\mathcal{F}_m(\lambda \to 0, x_0) = \phi_m(-x_0). \tag{128}$$

The y integrals in Eq.(117) can be performed using

$$\int_{-\infty}^{\infty} dy e^{-2\pi i k \frac{y}{L_2}} e^{-y^2/2\lambda^2} = \sqrt{2\pi}\lambda e^{-2\pi^2 k^2 \lambda^2/L_2^2}. \tag{129}$$

Using the integrals provided in Eq.(127) and Eq.(129), we now proceed to evaluate Eq.(117).

1. *Non-derivative or the zeroth-order coupling* γ

$$I^0_{[mr],[r']}(k_1,k_2) = \frac{\sqrt{L_{1x}}}{\sqrt{\ell}} \sum_{j\in\mathbb{Z}}\sum_{s\in\mathbb{Z}} \delta_{r'-r,jq+sp} e^{-2\pi i s k_1} e^{2\pi i s\left(k_2+\frac{r}{q}\right)\frac{L_{1y}}{L_2}} e^{\pi i s(s-1)\frac{p}{q}\frac{L_{1y}}{L_2}}$$
$$\frac{1}{2\pi\lambda^2}\int d^2\mathbf{r} e^{-2\pi i\left(k_2+\frac{r}{q}+s\frac{p}{q}\right)\frac{y}{L_2}} \varphi_m\left(x - sL_{1x} - \left(k_2+\frac{r}{q}\right)\frac{2\pi\ell^2}{L_2}\right) e^{-\mathbf{r}^2/(2\lambda^2)} = \tag{130}$$
$$\frac{\sqrt{L_{1x}}}{\sqrt{\ell}} \sum_{j\in\mathbb{Z}}\sum_{s\in\mathbb{Z}} \delta_{r'-r,jq+sp} e^{-2\pi i s k_1} e^{2\pi i s\left(k_2+\frac{r}{q}\right)\frac{L_{1y}}{L_2}} e^{\pi i s(s-1)\frac{p}{q}\frac{L_{1y}}{L_2}} e^{-2\pi^2\left(k_2+\frac{r}{q}+s\frac{p}{q}\right)^2\frac{\lambda^2}{L_2^2}} \mathcal{F}_m\left(\lambda, \left(s+\frac{r}{p}+k_2\frac{q}{p}\right)L_{1x}\right) \tag{131}$$

2. k_x *coupling*

$$I^x_{[mr],[r']}(k_1,k_2) = \frac{\sqrt{L_{1x}}}{\sqrt{\ell}} \sum_{j\in\mathbb{Z}}\sum_{s\in\mathbb{Z}} \delta_{r'-r,jq+sp} e^{-2\pi i s k_1} e^{2\pi i s\left(k_2+\frac{r}{q}\right)\frac{L_{1y}}{L_2}} e^{\pi i s(s-1)\frac{p}{q}\frac{L_{1y}}{L_2}}$$
$$\frac{1}{2\pi\lambda^2}\int d^2\mathbf{r} e^{-2\pi i\left(k_2+\frac{r}{q}+s\frac{p}{q}\right)\frac{y}{L_2}} \varphi_m\left(x - sL_{1x} - \left(k_2+\frac{r}{q}\right)\frac{2\pi\ell^2}{L_2}\right)\left(\frac{1}{i}\frac{\partial}{\partial x}\right) e^{-\mathbf{r}^2/(2\lambda^2)} \tag{132}$$
$$= \frac{\sqrt{L_{1x}}}{\sqrt{\ell}} \sum_{j\in\mathbb{Z}}\sum_{s\in\mathbb{Z}} \delta_{r'-r,jq+sp} e^{-2\pi i s k_1} e^{2\pi i s\left(k_2+\frac{r}{q}\right)\frac{L_{1y}}{L_2}} e^{\pi i s(s-1)\frac{p}{q}\frac{L_{1y}}{L_2}}$$
$$\frac{1}{2\pi\lambda^2}\frac{i}{\ell}\int d^2\mathbf{r} e^{-2\pi i\left(k_2+\frac{r}{q}+s\frac{p}{q}\right)\frac{y}{L_2}} \left(\ell\frac{\partial}{\partial x}\varphi_m\left(x - sL_{1x} - \left(k_2+\frac{r}{q}\right)\frac{2\pi\ell^2}{L_2}\right)\right) e^{-\mathbf{r}^2/(2\lambda^2)} \tag{133}$$

$$= \frac{\sqrt{L_{1x}}}{\sqrt{\ell}} \sum_{j\in\mathbb{Z}} \sum_{s\in\mathbb{Z}} \delta_{r'-r,jq+sp} e^{-2\pi i s k_1} e^{2\pi i s\left(k_2+\frac{r}{q}\right)\frac{L_{1y}}{L_2}} e^{\pi i s(s-1)\frac{p}{q}\frac{L_{1y}}{L_2}}$$
$$\frac{1}{2\pi\lambda^2}\frac{i\sqrt{m}}{\sqrt{2}\ell}\int d^2\mathbf{r} e^{-2\pi i\left(k_2+\frac{r}{q}+s\frac{p}{q}\right)\frac{y}{L_2}} \varphi_{m-1}\left(x - sL_{1x} - \left(k_2+\frac{r}{q}\right)\frac{2\pi\ell^2}{L_2}\right) e^{-\mathbf{r}^2/(2\lambda^2)}$$
$$- \frac{\sqrt{L_{1x}}}{\sqrt{\ell}} \sum_{j\in\mathbb{Z}} \sum_{s\in\mathbb{Z}} \delta_{r'-r,jq+sp} e^{-2\pi i s k_1} e^{2\pi i s\left(k_2+\frac{r}{q}\right)\frac{L_{1y}}{L_2}} e^{\pi i s(s-1)\frac{p}{q}\frac{L_{1y}}{L_2}}$$
$$\frac{1}{2\pi\lambda^2}\frac{i\sqrt{m+1}}{\sqrt{2}\ell}\int d^2\mathbf{r} e^{-2\pi i\left(k_2+\frac{r}{q}+s\frac{p}{q}\right)\frac{y}{L_2}} \varphi_{m+1}\left(x - sL_{1x} - \left(k_2+\frac{r}{q}\right)\frac{2\pi\ell^2}{L_2}\right) e^{-\mathbf{r}^2/(2\lambda^2)} \tag{134}$$
$$= \frac{\sqrt{L_{1x}}}{\sqrt{\ell}} \sum_{j\in\mathbb{Z}} \sum_{s\in\mathbb{Z}} \delta_{r'-r,jq+sp} e^{-2\pi i s k_1} e^{2\pi i s\left(k_2+\frac{r}{q}\right)\frac{L_{1y}}{L_2}} e^{\pi i s(s-1)\frac{p}{q}\frac{L_{1y}}{L_2}}$$
$$\frac{i\sqrt{m}}{\sqrt{2}\ell} e^{-2\pi^2\left(k_2+\frac{r}{q}+s\frac{p}{q}\right)^2\frac{\lambda^2}{L_2^2}} \mathcal{F}_{m-1}\left(\lambda, \left(s+\frac{r}{p}+k_2\frac{q}{p}\right)L_{1x}\right)$$
$$- \frac{\sqrt{L_{1x}}}{\sqrt{\ell}} \sum_{j\in\mathbb{Z}} \sum_{s\in\mathbb{Z}} \delta_{r'-r,jq+sp} e^{-2\pi i s k_1} e^{2\pi i s\left(k_2+\frac{r}{q}\right)\frac{L_{1y}}{L_2}} e^{\pi i s(s-1)\frac{p}{q}\frac{L_{1y}}{L_2}}$$
$$\frac{i\sqrt{m+1}}{\sqrt{2}\ell} e^{-2\pi^2\left(k_2+\frac{r}{q}+s\frac{p}{q}\right)^2\frac{\lambda^2}{L_2^2}} \mathcal{F}_{m+1}\left(\lambda, \left(s+\frac{r}{p}+k_2\frac{q}{p}\right)L_{1x}\right) \tag{135}$$

So, finally

$$I^x_{[mr],[r']}(k_1,k_2) = \frac{i}{\sqrt{2}\ell}\frac{\sqrt{L_{1x}}}{\sqrt{\ell}} \sum_{j\in\mathbb{Z}} \sum_{s\in\mathbb{Z}} \delta_{r'-r,jq+sp} e^{-2\pi i s k_1} e^{2\pi i s\left(k_2+\frac{r}{q}\right)\frac{L_{1y}}{L_2}} e^{\pi i s(s-1)\frac{p}{q}\frac{L_{1y}}{L_2}} e^{-2\pi^2\left(k_2+\frac{r}{q}+s\frac{p}{q}\right)^2\frac{\lambda^2}{L_2^2}}$$
$$\times\left(\sqrt{m}\mathcal{F}_{m-1}\left(\lambda, \left(s+\frac{r}{p}+k_2\frac{q}{p}\right)L_{1x}\right) - \sqrt{m+1}\mathcal{F}_{m+1}\left(\lambda, \left(s+\frac{r}{p}+k_2\frac{q}{p}\right)L_{1x}\right)\right) \tag{136}$$

3. k_y coupling

$$I^y_{[mr],[r']}(k_1,k_2) = \frac{\sqrt{L_{1x}}}{\sqrt{\ell}} \sum_{j\in\mathbb{Z}} \sum_{s\in\mathbb{Z}} \delta_{r'-r,jq+sp} e^{-2\pi i s k_1} e^{2\pi i s\left(k_2+\frac{r}{q}\right)\frac{L_{1y}}{L_2}} e^{\pi i s(s-1)\frac{p}{q}\frac{L_{1y}}{L_2}}$$
$$\frac{1}{2\pi\lambda^2}\int d^2\mathbf{r} e^{-2\pi i\left(k_2+\frac{r}{q}+s\frac{p}{q}\right)\frac{y}{L_2}} \varphi_{m}\left(x - sL_{1x} - \left(k_2+\frac{r}{q}\right)\frac{2\pi\ell^2}{L_2}\right)\left(\frac{1}{i}\frac{\partial}{\partial y}\right) e^{-\mathbf{r}^2/(2\lambda^2)} \tag{137}$$
$$= \frac{\sqrt{L_{1x}}}{\sqrt{\ell}} \sum_{j\in\mathbb{Z}} \sum_{s\in\mathbb{Z}} \delta_{r'-r,jq+sp} e^{-2\pi i s k_1} e^{2\pi i s\left(k_2+\frac{r}{q}\right)\frac{L_{1y}}{L_2}} e^{\pi i s(s-1)\frac{p}{q}\frac{L_{1y}}{L_2}}\left(k_2+\frac{r}{q}+s\frac{p}{q}\right)\frac{2\pi}{L_2}$$
$$\times e^{-2\pi^2\left(k_2+\frac{r}{q}+s\frac{p}{q}\right)^2\frac{\lambda^2}{L_2^2}} \mathcal{F}_{m}\left(\lambda, \left(s+\frac{r}{p}+k_2\frac{q}{p}\right)L_{1x}\right) \tag{138}$$

4. Minimal coupling

The minimal coupling term, $e\mathcal{M}_u A_\mu/c$, can be expressed in terms of the non-derivative coupling $I^0_{[mr],[r']}(k_1,k_2)$ and k_y coupling $I^y_{[mr],[r']}(k_1,k_2)$ as shown in this section. Let us call this term I_A, given as

$$I_A = -\frac{\sqrt{L_{1x}}}{\sqrt{\ell}} \sum_{j\in\mathbb{Z}} \sum_{s\in\mathbb{Z}} \delta_{r'-r,jq+sp} e^{-2\pi i s k_1} e^{2\pi i s\left(k_2+\frac{r}{q}\right)\frac{L_{1y}}{L_2}} e^{\pi i s(s-1)\frac{p}{q}\frac{L_{1y}}{L_2}}$$
$$\times\frac{1}{2\pi\lambda^2}\frac{\hbar}{\ell^2}\int d^2\mathbf{r} e^{-2\pi i\left(k_2+\frac{r}{q}+s\frac{p}{q}\right)\frac{y}{L_2}} x\varphi_{m}\left(x - sL_{1x} - \left(k_2+\frac{r}{q}\right)\frac{2\pi\ell^2}{L_2}\right) e^{-\mathbf{r}^2/(2\lambda^2)} \tag{139}$$

We can re-express the factor $x\varphi(x-x_0)$ as

$$x\varphi_m(x-x_0)= \frac{1}{\pi^{\frac{1}{4}}}\frac{1}{\sqrt{2^m m!}}e^{-(x-x_0)^2/2\ell^2}xH_m((x-x_0)/\ell)$$
$$= \ell\frac{1}{\pi^{\frac{1}{4}}}\frac{1}{\sqrt{2^m m!}}e^{-(x-x_0)^2/2\ell^2}(\frac{x-x_0}{\ell})H_m((x-x_0)/\ell)+x_0\phi_m(x-x_0). \tag{140}$$

Moreover, using recursion relation for Hermite polynomials

$$xH_m(x)=\frac{1}{2}H_{m+1}(x)+mH_{m-1}(x) \tag{141}$$

we have

$$x\varphi_m(x-x_0)= \ell\frac{1}{\pi^{\frac{1}{4}}}\frac{1}{\sqrt{2^m m!}}e^{-(x-x_0)^2/2\ell^2}\left(\frac{1}{2}H_{m+1}((x-x_0)/\ell)+mH_{m-1}((x-x_0)/\ell)\right)+x_0\varphi_m(x-x_0)$$
$$=\ell\sqrt{\frac{m+1}{2}}\varphi_{m+1}(x-x_0)+\ell\sqrt{\frac{m}{2}}\phi_{m-1}(x-x_0)+x_0\varphi_m(x-x_0) \tag{142}$$

Substituting Eq.(142) into Eq.(139), we have:

$$I_A=-\frac{\sqrt{L_{1x}}}{\sqrt{\ell}}\sum_{j\in\mathbb{Z}}\sum_{s\in\mathbb{Z}}\delta_{r'-r,jq+sp}e^{-2\pi isk_1}e^{2\pi is\left(k_2+\frac{r}{q}\right)\frac{L_{1y}}{L_2}}e^{\pi is(s-1)\frac{p}{q}\frac{L_{1y}}{L_2}}$$
$$\times\frac{1}{2\pi\lambda^2}\frac{\hbar}{\ell}\int d^2\mathbf{r}e^{-2\pi i\left(k_2+\frac{r}{q}+s\frac{p}{q}\right)\frac{y}{L_2}}\Bigg[\sqrt{\frac{m+1}{2}}\varphi_{m+1}(x-sL_{1x}-\left(k_2+\frac{r}{q}\right)\frac{2\pi\ell^2}{L_2})$$
$$+\sqrt{\frac{m}{2}}\varphi_{m-1}(x-sL_{1x}-\left(k_2+\frac{r}{q}\right)\frac{2\pi\ell^2}{L_2})$$
$$+\frac{1}{\ell}(sL_{1x}+\left(k_2+\frac{r}{q}\right)\frac{2\pi\ell^2}{L_2})\varphi_m(x-sL_{1x}-\left(k_2+\frac{r}{q}\right)\frac{2\pi\ell^2}{L_2})\Bigg]e^{-\mathbf{r}^2/(2\lambda^2)}. \tag{143}$$

Using the fact $\ell^2=\frac{qL_{1x}L_2}{2\pi p}$, note that

$$\frac{1}{\ell^2}(sL_{1x}+\left(k_2+\frac{r}{q}\right)\frac{2\pi\ell^2}{L_2})=\frac{2\pi}{L_2}(k_2+\frac{r}{p}+s\frac{p}{q}). \tag{144}$$

Thus we have

$$I_A=-\hbar\Bigg[\frac{1}{\sqrt{2}\ell}\left(\sqrt{m}I^0_{[m-1r],[r']}(k_1,k_2)+\sqrt{m+1}I^0_{[m+1r],[r']}(k_1,k_2)\right)+I^y_{[mr],[r']}(k_1,k_2)\Bigg] \tag{145}$$

5. *Closed form expression for c-f coupling at finite field*

The closed form expression for the c-f coupling at finite $\mathbf{B}$ then reads

$$H^{cf,\tau}\approx\sum_{k\in[0,1)\otimes[0,1/q)]}\sum_{a=1}^{4}\sum_{b=1}^{2}\sum_{m=0}^{m_{a,\tau}}\sum_{r=0}^{p-1}\sum_{r'=0}^{q-1}\Bigg[\begin{pmatrix}\gamma I^0_{[mr],[r']}(k)\sigma_0+v'_*(\tau I^x_{[mr],[r']}(k)\sigma_x+I^y_{[mr],[r']}(k)\sigma_y)\\ 0_{2\times2}\end{pmatrix}_{ab}+ \tag{146}$$

$$\begin{pmatrix}-v'_*\frac{1}{\sqrt{2}\ell}\left(\sqrt{m}I^0_{[m-1r],[r']}(k)+\sqrt{m+1}I^0_{[m+1r],[r']}(k)\right)\sigma_y-v'_*I^y_{[mr],[r']}(k)\sigma_y\\ 0_{2\times2}\end{pmatrix}_{ab}\Bigg]c^\dagger_{a\tau krm}f_{b\tau kr}. \tag{147}$$

C. Low field Analysis of c-f coupling

For analyzing the low **B** structure for c-f hybridization matrix, given in Eq.(147), we will find it useful to first discuss recursion relations for $\mathcal{F}_m(\lambda, x_0)$ defined in Eq.(127). We start by considering the generating function for $\mathcal{H}_m(x, y)$ (defined in Eq.(118))

$$e^{xt+yt^2} = \sum_{n=0}^{\infty} \frac{t^n}{n!} \mathcal{H}_n(x, y) . \tag{148}$$

Using the generating function, we can derive the following recursion relations

$$\frac{\partial}{\partial t} e^{xt+yt^2} = (x + 2yt) e^{xt+yt^2} \Rightarrow \sum_{n=1}^{\infty} \frac{t^{n-1}}{(n-1)!} \mathcal{H}_n(x, y) = \sum_{n=0}^{\infty} \frac{t^n}{n!} x \mathcal{H}_n(x, y) + \sum_{n=0}^{\infty} \frac{t^{n+1}}{n!} 2y \mathcal{H}_n(x, y) \tag{149}$$

$$\sum_{n=0}^{\infty} \frac{t^n}{n!} \mathcal{H}_{n+1}(x, y) - \sum_{n=1}^{\infty} \frac{t^n}{(n-1)!} 2y \mathcal{H}_{n-1}(x, y) = \sum_{n=0}^{\infty} \frac{t^n}{n!} x \mathcal{H}_n(x, y) \tag{150}$$

$$n = 0 : \ \mathcal{H}_1(x, y) = x \mathcal{H}_0(x, y) \tag{151}$$

$$n > 0 : \ \mathcal{H}_{n+1}(x, y) - 2yn \mathcal{H}_{n-1}(x, y) = x \mathcal{H}_n(x, y) \tag{152}$$

Therefore

$$n = 0 : \ \sqrt{2} \mathcal{F}_1(\lambda, x_0) = -2x_0 \frac{\ell}{\ell^2 + \lambda^2} \mathcal{F}_0(\lambda, x_0) \tag{153}$$

$$n > 0 : \ \sqrt{2^{n+1}(n+1)!} \mathcal{F}_{n+1}(\lambda, x_0) + 2\left(1 - \frac{2\lambda^2}{\ell^2 + \lambda^2}\right) n \sqrt{2^{n-1}(n-1)!} \mathcal{F}_{n-1}(\lambda, x_0) = -2x_0 \frac{\ell}{\ell^2 + \lambda^2} \sqrt{2^n n!} \mathcal{F}_n(\lambda, x_0)$$

$$\Longrightarrow \left(1 + \frac{\lambda^2}{\ell^2}\right) \frac{1}{\sqrt{2}} \left(\sqrt{n+1} \mathcal{F}_{n+1}(\lambda, x_0) + \left(1 - \frac{2\lambda^2}{\ell^2 + \lambda^2}\right) \sqrt{n} \mathcal{F}_{n-1}(\lambda, x_0)\right) = -\frac{x_0}{\ell} \mathcal{F}_n(\lambda, x_0)$$

$$\Longrightarrow \left(1 + \frac{\lambda^2}{\ell^2}\right) \sqrt{\frac{n+1}{2}} \mathcal{F}_{n+1}(\lambda, x_0) + \left(1 - \frac{\lambda^2}{\ell^2}\right) \sqrt{\frac{n}{2}} \mathcal{F}_{n-1}(\lambda, x_0) = -\frac{x_0}{\ell} \mathcal{F}_n(\lambda, x_0) . \tag{154}$$

From Eq.(138), we have

$$I^y_{[mr],[r']} = \frac{\sqrt{L_{1x}}}{\sqrt{\ell}} \sum_{j\in\mathbb{Z}} \sum_{s\in\mathbb{Z}} \delta_{r'-r, jq+sp} e^{-2\pi i s k_1} e^{2\pi i s\left(k_2 + \frac{r}{q}\right)\frac{L_{1y}}{L_2}} e^{\pi i s(s-1)\frac{p}{q}\frac{L_{1y}}{L_2}} \left(k_2 + \frac{r}{q} + s\frac{p}{q}\right) \frac{2\pi}{L_2}$$

$$\times e^{-2\pi^2\left(k_2 + \frac{r}{q} + s\frac{p}{q}\right)^2 \frac{\lambda^2}{L_2^2}} \mathcal{F}_m\left(\lambda, \left(s + \frac{r}{p} + k_2 \frac{q}{p}\right) L_{1x}\right) \tag{155}$$

$$= \frac{p}{q} \frac{2\pi\ell^2}{\ell L_{1x} L_2} \frac{\sqrt{L_{1x}}}{\sqrt{\ell}} \sum_{j\in\mathbb{Z}} \sum_{s\in\mathbb{Z}} \delta_{r'-r, jq+sp} e^{-2\pi i s k_1} e^{2\pi i s\left(k_2 + \frac{r}{q}\right)\frac{L_{1y}}{L_2}} e^{\pi i s(s-1)\frac{p}{q}\frac{L_{1y}}{L_2}} (qk_2 + r + sp) \frac{L_{1x}}{p} \frac{1}{\ell}$$

$$\times e^{-2\pi^2\left(k_2 + \frac{r}{q} + s\frac{p}{q}\right)^2 \frac{\lambda^2}{L_2^2}} \mathcal{F}_m\left(\lambda, (sp + r + qk_2) \frac{L_{1x}}{p}\right) . \tag{156}$$

. Now using the fact $\frac{2\pi p \ell^2}{q L_{1x} L_2} = 1$ and the recursion relation given in Eq.(154), we have

$$I^y_{[mr],[r']}(k) = \frac{-1}{\ell} \frac{\sqrt{L_{1x}}}{\sqrt{\ell}} \sum_{j\in\mathbb{Z}} \sum_{s\in\mathbb{Z}} \delta_{r'-r, jq+sp} e^{-2\pi i s k_1} e^{2\pi i s\left(k_2 + \frac{r}{q}\right)\frac{L_{1y}}{L_2}} e^{\pi i s(s-1)\frac{p}{q}\frac{L_{1y}}{L_2}}$$

$$\times e^{-2\pi^2\left(k_2 + \frac{r}{q} + s\frac{p}{q}\right)^2 \frac{\lambda^2}{L_2^2}} \left(\left(1 + \frac{\lambda^2}{\ell^2}\right) \sqrt{\frac{m+1}{2}} \mathcal{F}_{m+1}\left(\lambda, (sp + r + qk_2) \frac{L_{1x}}{p}\right)\right.$$

$$\left. + \left(1 - \frac{\lambda^2}{\ell^2}\right) \sqrt{\frac{m}{2}} \mathcal{F}_{m-1}\left(\lambda, (sp + r + qk_2) \frac{L_{1x}}{p}\right)\right) \tag{157}$$

$$= -\frac{1}{\sqrt{2}\ell} \left(\left(1 + \frac{\lambda^2}{\ell^2}\right) \sqrt{m+1} I^0_{[m+1r],[r']}(k) + \left(1 - \frac{\lambda^2}{\ell^2}\right) \sqrt{m} I^0_{[m-1r],[r']}(k)\right) . \tag{158}$$

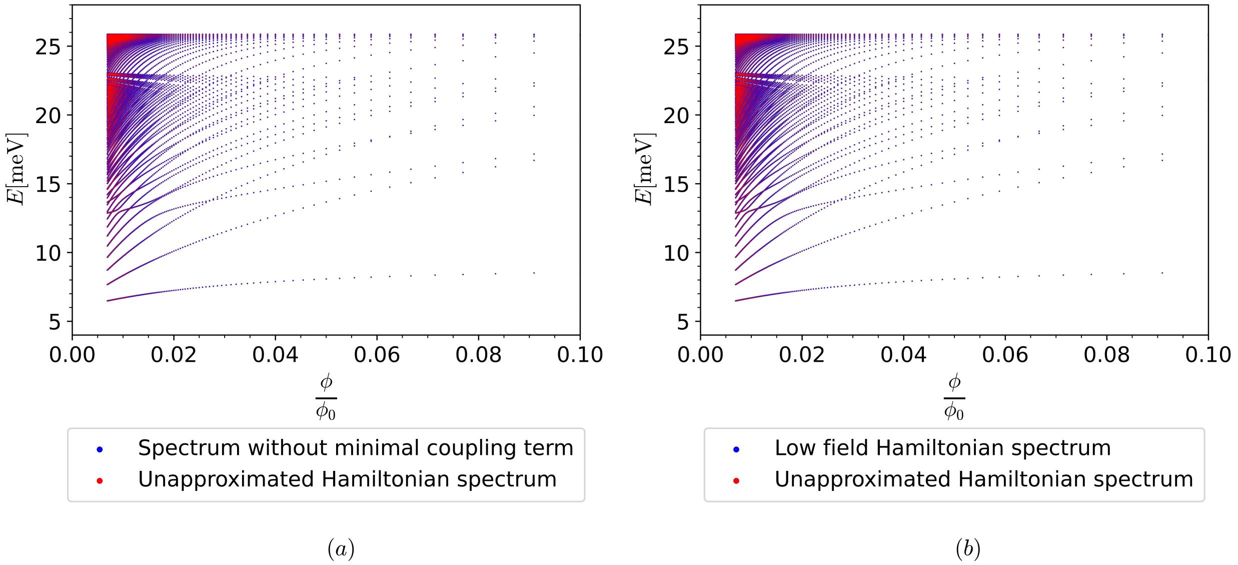

Supplementary Figure 3. These figures justify dropping $\mathcal{O}(\frac{\lambda^2}{\ell^2})$ in the prefactor of $I^{0,x,y}$ in the c-f hybridization matrix.(a)Comparison of the unapproximated Hamiltonian spectrum which includes the minimal coupling to the one without minimal coupling at valley $\mathbf{K}'$ at CNP.(b)Comparison of the unapproximated Hamiltonian spectrum to the one obtained using low field c-f coupling at valley $\mathbf{K}'$ at CNP. These figures correspond to parameters $w_0/w_1 = 0.7$,$J = 18.27$meV, $U_1 = 51.72$meV, $\gamma = -39.11$meV, $v_*' = 1.624$eV.Å, $v_* = -4.483$eV.Å, $M = 3.248$meV and $\lambda = 0.3792L_m$. We set $k_{1,2} = 0$ and $m_{max} = \lceil\frac{q-3}{2}\rceil$.

where $I^0_{[mr],[r']}$ is defined in Eq.(131). Using the above derived form for $I^y_{[mr],[r']}$ in Eq(158), the minimal coupling term, given in Eq.(145), can be rewritten as

$$-\frac{I_A}{\hbar} = \frac{\lambda^2}{\ell^2}\frac{1}{\sqrt{2}\ell}\left(\sqrt{m}I^0_{[m-1r],[r']}(k) - \sqrt{m+1}I^0_{[m+1r],[r']}(k)\right) \tag{159}$$

Note that this is an $\mathcal{O}(\frac{\lambda^2}{\ell^2})$ term. At low $\mathbf{B}$, i.e $\lambda^2 \ll \ell^2$, this term is negligible as anticipated. This is confirmed numerically as shown in Supplementary Fig.(3a). Note that we however still need to keep the full $\frac{\lambda^2}{\ell^2}$ dependence within $\mathcal{F}_m$ for accuracy in low $\mathbf{B}$ range as shown discussed in Supplementary Fig.(4). Keeping this in mind, let us now dwell into a low $\mathbf{B}$ limit wherein we can drop the $\mathcal{O}(\frac{\lambda^2}{\ell^2})$ term in the recursion relation Eq.(154), i.e.

$$\sqrt{\frac{n+1}{2}}\mathcal{F}_{n+1}\left(\lambda, x_0\right) + \sqrt{\frac{n}{2}}\mathcal{F}_{n-1}\left(\lambda, x_0\right) = -\frac{x_0}{\ell}\mathcal{F}_n\left(\lambda, x_0\right). \tag{160}$$

Moreover using Eq.(158), $I^y_{[mr],[r']}$ in this limit reads

$$I^y_{[mr],[r']}(k) = -\frac{1}{\sqrt{2}\ell}\left(\sqrt{m+1}I^0_{[m+1r],[r']}(k) + \sqrt{m}I^0_{[m-1r],[r']}(k)\right). \tag{161}$$

Now using the above and Eq.(136), we have

$$I^x_{[mr],[r']}(k) + iI^y_{[mr],[r']}(k) \approx -i\frac{\sqrt{2}}{\ell}\sqrt{m+1}I^0_{[m+1r],[r']}(k) \tag{162}$$

$$I^x_{[mr],[r']}(k) - iI^y_{[mr],[r']}(k) \approx i\frac{\sqrt{2}}{\ell}\sqrt{m}I^0_{[m-1r],[r']}(k). \tag{163}$$

Then at low field we can re-express the c-f coupling as

$$h^{+1}_{[amr][br']}(k) = \begin{pmatrix} \gamma I^0_{[mr],[r']}(k) & i\frac{\sqrt{2}v_*'}{\ell}\sqrt{m}I^0_{[m-1r],[r']}(k) \\ -i\frac{\sqrt{2}v_*'}{\ell}\sqrt{m+1}I^0_{[m+1r],[r']}(k) & \gamma I^0_{[mr],[r']}(k) \\ 0 & 0 \\ 0 & 0 \end{pmatrix}, \tag{164}$$

$$h^{-1}_{[amr][br']}(k) = \begin{pmatrix} \gamma I^0_{[mr],[r']}(k) & i\frac{\sqrt{2}v'_*}{\ell}\sqrt{m+1}I^0_{[m+1r],[r']}(k) \\ -i\frac{\sqrt{2}v'_*}{\ell}\sqrt{m}I^0_{[m-1r],[r']}(k) & \gamma I^0_{[mr],[r']}(k) \\ 0 & 0 \\ 0 & 0 \end{pmatrix}. \tag{165}$$

Supplementary Fig.(3b) shows that dropping $\mathcal{O}(\frac{\lambda^2}{\ell^2})$ in Eq.(154) is indeed an excellent approximation. It is thus justified to study the low field c-f hybridization matrix, given in Eq.(164) and Eq.(165), in pursuit of obtaining an analytical solution.

Supplementary note 5. SINGULAR VALUES FOR I^0

In this section, we study the singular values of $I^0_{m,r'}(k) = I^0_{[m0],[r']}(k)$, where $I^0_{[m0],[r']}(k)$ is defined in Eq.(131). As discussed in the main text, these singular values controls the hybridization strength between the heavy and conduction fermions in finite $\mathbf{B}$. Let us start by considering the singular value decomposition of $I^0_{m,r'}(k)$:

$$I^0_{m,r'}(k) = U_{mm'}\Sigma_{m'\tilde{r}}V_{\tilde{r}r'}, \tag{166}$$

where U, V are are $(m_{a,\tau}+1)\times(m_{a,\tau}+1)$ and $q\times q$ unitary matrices respectively, and the $(m_{a,\tau}+1)\times q$ rectangular matrix Σ contains the singular values along the main diagonal and zeros elsewhere, where summation convention on repeated indices is implied. The columns of matrix U are the eigenvectors of the following matrix

$$\Lambda^0_{mm'}(k) = \sum_{r'=0}^{q-1} I^0_{mr'}(k) I^{0^*}_{m'r'}(k). \tag{167}$$

Since r' is being summed over, we are allowed to shift its range to $-\lfloor\frac{q}{2}\rfloor, -\lfloor\frac{q}{2}\rfloor+1,\ldots,q-1-\lfloor\frac{q}{2}\rfloor$ and thus set $j=0$ in Eq.(131) based on the discussion in main text. Since $\mathcal{F}_m$ in Eq.(131) decays exponentially at $\pm\lfloor\frac{q}{2}\rfloor$, in the low field limit, we can replace the bounds of this sum by $\pm\infty$ and have

$$\Lambda^0_{mm'}(k) \approx \frac{L_{1x}}{\ell}\sum_{r'=-\infty}^{\infty} e^{-4\pi^2\frac{\lambda^2}{L_2^2}\left(k_2+\frac{r'}{q}\right)^2}\mathcal{F}_m\left(\lambda,(r'+k_2q)L_{1x}\right)\mathcal{F}_{m'}\left(\lambda,(r'+k_2q)L_{1x}\right). \tag{168}$$

Using the Dirac comb identity, $\sum_{r'\in\mathbb{Z}}\delta(\rho-r') = \sum_{t\in\mathbb{Z}} e^{2\pi i t\rho}$, we can convert this summation to an integral as

$$\begin{aligned}\Lambda^0_{mm'}(k) &\approx \frac{L_{1x}}{\ell}\sum_{t\in\mathbb{Z}}\int_{-\infty}^{\infty} d\rho e^{i2\pi t\rho} e^{-4\pi^2\frac{\lambda^2}{L_2^2}\left(k_2+\frac{\rho}{q}\right)^2}\mathcal{F}_m\left(\lambda,(\rho+k_2q)L_{1x}\right)\mathcal{F}_{m'}\left(\lambda,(\rho+k_2q)L_{1x}\right) \\ &= \frac{1}{\ell}\sum_{t\in\mathbb{Z}} e^{-i2\pi t k_2 q}\int_{-\infty}^{\infty} d\rho e^{i2\pi t\rho/L_{1x}} e^{-\frac{\lambda^2}{\ell^2}\frac{\rho^2}{\ell^2}}\mathcal{F}_m(\lambda,\rho)\mathcal{F}_{m'}(\lambda,\rho).\end{aligned} \tag{169}$$

Because $2\pi t/L_{1x}$ is a large wavevector compared to the length scale at which the h.o. wavefunctions vary, $\sim\ell$, i.e. $\frac{L_{1x}}{2\pi t}\ll\ell$ at low field, the overlaps are exponentially suppressed in $(\frac{2\pi t\ell}{L_{1x}})^2$. We can thus set $t=0$ in the above sum. Moreover since the exponential factor contains $\frac{\lambda^2}{\ell^2}$, the off-diagonal terms can be neglected and thus $\Lambda^0_{mm'}$ is diagonal up to a good approximation. This implies that the matrix U is close to identity and the low $\mathbf{B}$ singular values can be approximated as $\Sigma_m = \sqrt{\Lambda^0_{mm}}$. Note that the information of magnetic quantum number k gets dissolved automatically due to the low field limit where the magnetic sub-bands are not k dispersive. We thus have

$$\Sigma^2_m = \frac{1}{\ell}\int_{-\infty}^{\infty} d\rho e^{-\frac{\lambda^2}{\ell^2}\frac{\rho^2}{\ell^2}}\mathcal{F}_m(\lambda,\rho)\mathcal{F}_m(\lambda,\rho) \tag{170}$$

$\mathcal{F}_m(\lambda,\rho)$ defined in Eq.(127) can re-expressed as:

$$\mathcal{F}_m(\lambda,\rho) = \frac{1}{\pi^{\frac{1}{4}}\sqrt{2^m m!}}\sqrt{\frac{1}{1+(\frac{\lambda}{\ell})^2}}e^{-\frac{1}{2}(\frac{\rho}{\ell})^2/(1+(\frac{\lambda}{\ell})^2)}\mathcal{H}_m\left(\frac{-2(\frac{\rho}{\ell})}{1+(\frac{\lambda}{\ell})^2},\frac{2(\frac{\lambda}{\ell})^2}{1+(\frac{\lambda}{\ell})^2}-1\right) \tag{171}$$

Naively one would expect that dropping $\mathcal{O}(\frac{\lambda^2}{\ell^2})$ in $\mathcal{F}_m(\lambda,x_0)$, i.e. replacing $\mathcal{F}_m(\lambda,x_0)$ by $\varphi(-x_0)$, to be good enough approximation at low field. However as discussed in main text, the fairly strong m dependence of singular values gets compromised under such an approximation, as shown in Supplementary Fig.(4a).

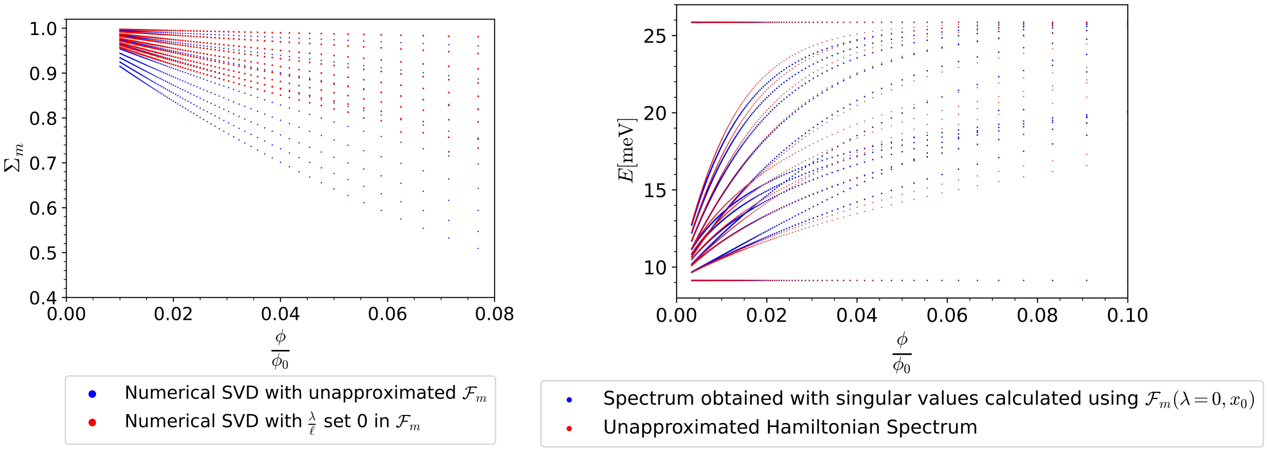

(a) (b)

Supplementary Figure 4. These figures illustrate the importance of including the $\mathcal{O}(\frac{\lambda^2}{\ell^2})$ in $\mathcal{F}_m(\lambda, x_0)$ for obtaining the correct interacting heavy fermion Hofstadter spectrum. (a) The comparison of numerically obtained singular values for I^0 with the ones obtained numerically post setting $\frac{\lambda}{\ell} = 0$ in $\mathcal{F}_m(\lambda, x_0)$. (b) The figure comparing unapproximated Hamiltonian spectrum with the one calculated using singular values marked in red from the left panel at $w_0/w_1 = 0.7$ in flat band limit at valley $\mathbf{K}'$ at CNP. m_{max} is set to 5 for illustration.

Let $\rho/\ell = x$ and $\kappa = \lambda/\ell$. Then

$$\Sigma_m^2 = \frac{1}{\sqrt{\pi}} \frac{1}{2^m m!} \frac{1}{1+\kappa^2} \int_{-\infty}^{\infty} dx e^{-\left(\kappa^2 + \frac{1}{1+\kappa^2}\right)x^2} \mathcal{H}_m^2\left(\frac{-2}{1+\kappa^2}x, \frac{\kappa^2-1}{\kappa^2+1}\right) \tag{172}$$

Now, using the result from [6], we have

$$\int_{-\infty}^{\infty} dx \mathcal{H}_m(ax+b, y)\mathcal{H}_n(cx+d, y) e^{-fx^2+\alpha x} = \sqrt{\frac{\pi}{f}} e^{\frac{\alpha^2}{4f}} \mathcal{H}_{m,n}\left(b + \frac{a\alpha}{2f}, y + \frac{a^2}{2f}; d + \frac{c\alpha}{2f}, z + \frac{c^2}{2f} \mid \frac{ac}{2f}\right) \tag{173}$$

where

$$\mathcal{H}_{m,n}\left(x, y; w, z|\beta\right) = \sum_{k=0}^{\min(m,n)} \frac{m!n!}{(n-k)!(m-k)!k!} \beta^k \mathcal{H}_{m-k}(x,y)\mathcal{H}_{n-k}(w,z), \tag{174}$$

with min representing minimum and the two variable Hermite polynomial $\mathcal{H}_n(x, y)$ is defined in Eq.(118), we have

$$\Sigma_m^2 = \frac{1}{\sqrt{\xi(\kappa)}} \frac{1}{2^m m!} \mathcal{H}_{mm}(0, \frac{\kappa^6}{\xi(\kappa)}; 0, \frac{\kappa^6}{\xi(\kappa)} \mid \frac{2}{\xi(\kappa)}), \tag{175}$$

where $\xi(\kappa)$ is given as

$$\xi(\kappa) = (1+\kappa^2+\kappa^4)(1+\kappa^2). \tag{176}$$

Thus

$$\Sigma_m = \left(\frac{1}{\sqrt{\xi(\kappa)}} \frac{1}{2^m m!} \mathcal{H}_{mm}(0, \frac{\kappa^6}{\xi(\kappa)}; 0, \frac{\kappa^6}{\xi(\kappa)} \mid \frac{2}{\xi(\kappa)})\right)^{\frac{1}{2}}. \tag{177}$$

Note that

$$\kappa^2 = \frac{\lambda^2}{\ell^2} = \frac{2\pi\lambda^2}{A_{uc}} \frac{p}{q} = \frac{2\pi\lambda^2}{A_{uc}} \frac{\phi}{\phi_0} \tag{178}$$

where A_{uc} is moire unit cell area. Since Σ_m^2 is an analytic function in κ^2, we have an expression for the singular values continuously down to zero flux. Also note that as $\frac{\phi}{\phi_0} \to 0 \implies \kappa^2 \to 0 \implies \xi(\kappa) \to 1 \implies \mathcal{H}_{mm}(0, \frac{\kappa^6}{\xi(\kappa)}; 0, \frac{\kappa^6}{\xi(\kappa)} \mid$

$\frac{2}{\xi(\kappa)}) \to 2^m m! \implies \mathit{\Sigma}_m \to 1$, consistent with numerical results. At extremely low flux, we can approximate the singular values as

$$\mathrm{lt}_{\kappa\to 0}\mathit{\Sigma}_m(\kappa) = 1 - \left(m + \frac{1}{2}\right)\kappa^2, \tag{179}$$

where lt represents limit. We thus see that the singular values are approaching 1 at zero flux with a negative slope of $\left(m+\frac{1}{2}\right)\frac{2\pi\lambda^2}{A_{uc}}$ as shown in Supplementary Fig.(5c). Note that even at extremely low flux, the m dependence of singular values grows linearly.

It would prove feasible to discuss the asymptote of $\mathit{\Sigma}_m^2$ for large values of m. Let us start expanding the four variable Hermite polynomial in Eq.(175).

$$\mathit{\Sigma}_m^2 = \frac{1}{\sqrt{\xi(\kappa)}}\frac{1}{2^m m!}\Big[\sum_{k=0}^{m}\frac{m!m!\tau^k}{(m-k)!(m-k)!k!}\mathcal{H}_{m-k}(0,y)\mathcal{H}_{m-k}(0,y)\Big] \tag{180}$$

where for brevity we denote $\frac{\kappa^6}{\xi(\kappa)}$ and $\frac{2}{\xi(\kappa)}$ by y and β respectively. Then

$$\mathit{\Sigma}_m^2 = \frac{1}{\sqrt{\xi(\kappa)}}\frac{1}{2^m}\Big[\frac{1}{m!}\mathcal{H}_m(0,y)\mathcal{H}_m(0,y)\Big] \tag{181}$$

$$+\frac{1}{\sqrt{\xi(\kappa)}}\frac{1}{2^m}\Big[\sum_{k=1}^{m-1}\frac{m!\beta^k}{(m-k)!(m-k)!k!}\mathcal{H}_{m-k}(0,y)\mathcal{H}_{m-k}(0,y)\Big] \tag{182}$$

$$+\frac{1}{\sqrt{\xi(\kappa)}}\frac{1}{2^m}\beta^m, \tag{183}$$

where we divided the sum over k into $k=0$, $k=1\ldots m-1$ and $k=m$, shown in Eq.(181), Eq.(182) and Eq.(183) respectively. Now note that $\mathcal{H}_m(0,y)$ is non-zero only if m is even, so

$$\mathcal{H}_m(0,y) = (1-\mathrm{mod}(m,2))\, m!\frac{y^{\frac{m}{2}}}{\frac{m}{2}!} \tag{184}$$

$$\implies \mathcal{H}_m^2(0,y) = (1-\mathrm{mod}(m,2))\,\frac{m!m!y^m}{\frac{m}{2}!\frac{m}{2}!}., \tag{185}$$

where mod$(m,2)$ represents the remainder of $m/2$. Using Eq.(185), we have

$$\mathit{\Sigma}_m^2 = \frac{1}{\sqrt{\xi(\kappa)}}\left((1-\mathrm{mod}(m,2))\left(\frac{y}{2}\right)^m\frac{m!}{\frac{m}{2}!\frac{m}{2}!} + \left(\frac{\beta}{2}\right)^m\right)$$
$$+\frac{1}{\sqrt{\xi(\kappa)}}\frac{1}{2^m}\Big[\sum_{k=1}^{m-1}(1-\mathrm{mod}(m-k,2))\frac{m!\beta^k y^{m-k}}{(\frac{m-k}{2})!(\frac{m-k}{2})!k!}\Big]. \tag{186}$$

Now consider the natural log (ln) of the quantity

$$\bar{A}(m) = \frac{m!}{(\frac{m}{2})!(\frac{m}{2})!} \tag{187}$$

$$\implies \ln(\bar{A}(m)) = \ln(m!) - 2\ln\left(\left(\frac{m}{2}\right)!\right) \approx m\ln(2) \tag{188}$$

$$\implies \bar{A}(m) = 2^m, \tag{189}$$

where in Eq.(188), we use the Stirling's approximation for large m, i.e. $\ln(m!) = m\ln(m) - m$. Similarly consider

$$\bar{B}_k(m) = \frac{m!}{(\frac{m-k}{2})!(\frac{m-k}{2})!} \tag{190}$$

$$\implies \ln(\bar{B}_k(m)) = \ln(m!) - 2\ln\left(\left(\frac{m-k}{2}!\right)\right) - \ln(k!) \tag{191}$$

$$\approx m\ln(m) - m\ln\left(\frac{m-k}{2}\right) + k\ln\left(\frac{m-k}{2}\right) - k\ln(k) \tag{192}$$

$$= m\ln\left(\frac{2m}{m-k}\right) + k\ln\left(\frac{m-k}{2k}\right) \tag{193}$$

$$\implies \bar{B}_k(m) = 2^{m-k}e^{m\ln\left(\frac{m}{m-k}\right)+k\ln\left(\frac{m-k}{k}\right)} \tag{194}$$

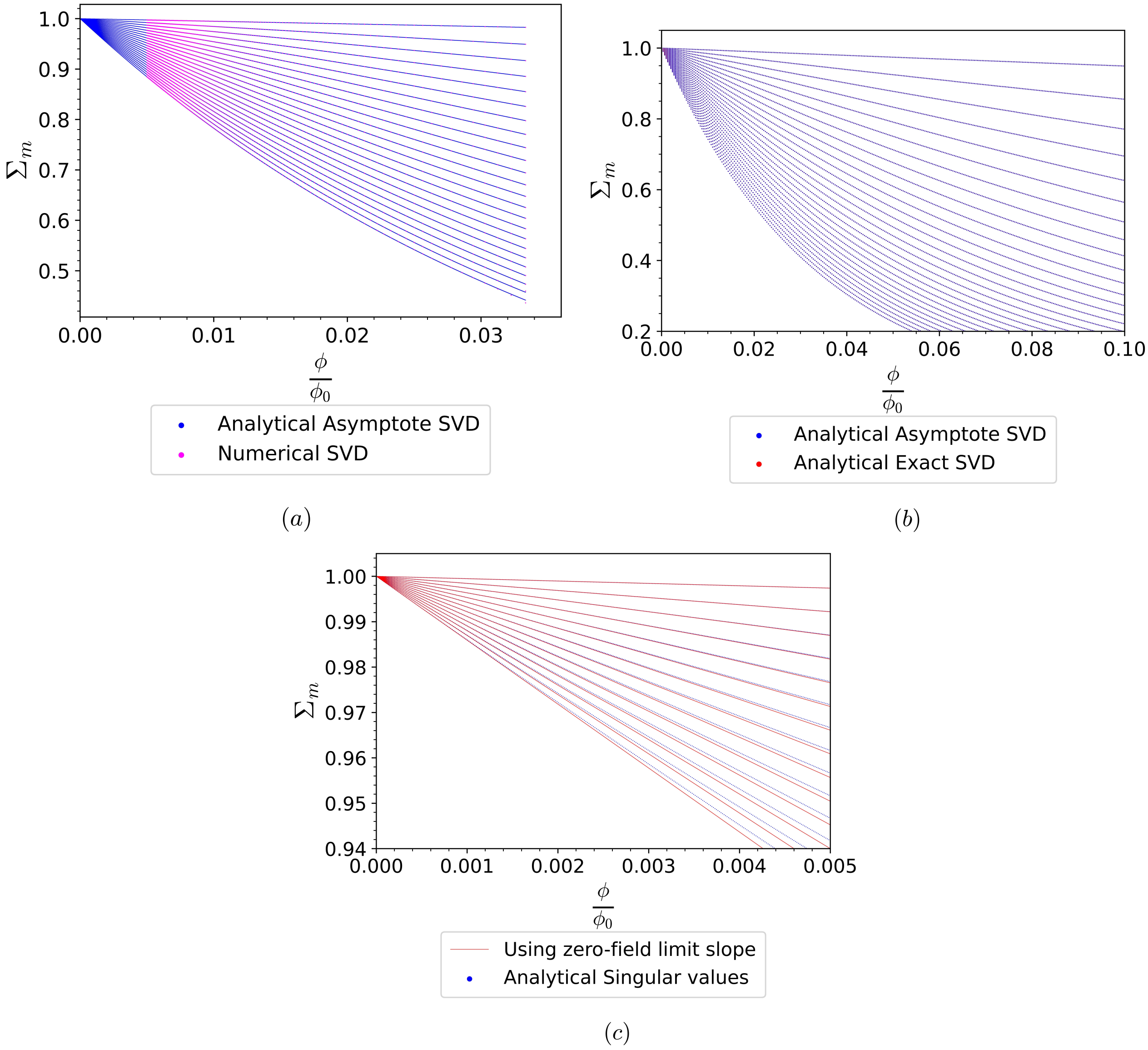

Supplementary Figure 5. (a) The comparison of first 25 numerically obtained singular values with the ones obtained using the analytical asymptotes for singular values. (b) The comparison of asymptotes with exact expression for singular values. (c) Comparing $1-(m+\frac{1}{2})\kappa^2$ with analytically computed singular values at extremely low field.

where in Eq.(192) we again have used the Stirling's approximation. Now substituting back Eq.(189) and Eq.(194) in Eq.(186) we have the following asymptote:

- If m is even

$$\Sigma_m^2 = \frac{1}{\sqrt{\xi(\kappa)}}\left[\left(\frac{1}{\xi(\kappa)}\right)^m + \left(\frac{\kappa^6}{\xi(\kappa)}\right)^m + \sum_{k=2,4,6,\ldots}^{m-2} e^{m\ln(\frac{m}{m-k})+k\ln(\frac{m-k}{k})}\left(\frac{1}{\xi(\kappa)}\right)^k\left(\frac{\kappa^6}{\xi(\kappa)}\right)^{m-k}\right]. \tag{195}$$

- If m is odd

$$\Sigma_m^2 = \frac{1}{\sqrt{\xi(\kappa)}}\left[\left(\frac{1}{\xi(\kappa)}\right)^m + \sum_{k=1,3,5,\ldots}^{m-1} e^{m\ln(\frac{m}{m-k})+k\ln(\frac{m-k}{k})}\left(\frac{1}{\xi(\kappa)}\right)^k\left(\frac{\kappa^6}{\xi(\kappa)}\right)^{m-k}\right]. \tag{196}$$

Supplementary Fig.(5b) shows that the asymptotic expression for singular values, given in Eq.(195) and Eq.(195), exactly matches the analytical singular values, given in Eq.(177), for all $m \neq 0$ (since the asymptotic formulation does not hold for $m = 0$). In the Supplementary Fig.(5b), $m = 0$ singular value on the asymptotic branch has been calculated using the analytical value, i.e. $\Sigma_0 = \frac{1}{\sqrt{\sqrt{\xi(\kappa)}}}$.

Supplementary note 6. DISCUSSION FOR VALLEY K

In this section we discuss finite **B** THFM at valley **K**, i.e. $\tau = +1$. Note that summation convention on repeated indices is implied unless explicitly stated. Using the SVD decomposition discussed we can re-write the matrix elements as

$$h^1_{[1m0],[1r']} c^\dagger_{11k0ms} f_{11kr's} = \gamma \Sigma_{m',\bar{r}} c^\dagger_{11k0ms} U_{mm'} V_{\bar{r},r'} f_{11kr's} = \sum_{m=0}^{m_{1,+1}} \sum_{\bar{r}=0}^{m_{1,+1}} \gamma \Sigma_m \delta_{m\bar{r}} c^\dagger_{11k0ms} \bar{f}_{11k\bar{r}s}, \tag{197}$$

where we used the fact that U is an identity matrix and the rectangular matrix $\Sigma_{m,r'}$ is non-zero only along its main diagonal.

$$\begin{aligned} h^1_{[2m0],[1r']} c^\dagger_{21k0m} f_{11kr's} &= -i\sqrt{2}\frac{v'_*}{\ell} \sum_{m=0}^{m_{2,+1}} \sqrt{m+1} c^\dagger_{21k0m} \Sigma_{m+1,\bar{r}} V_{\bar{r},r'} f_{11kr's} \\ &= -i\sqrt{2}\frac{v'_*}{\ell} \sum_{m=0}^{m_{2,+1}} \sum_{\bar{r}=0}^{m_{2,+1}+1} \sqrt{m+1} \Sigma_{m+1} \delta_{m+1,\bar{r}} c^\dagger_{21k0ms} \bar{f}_{11k\bar{r}s} \end{aligned} \tag{198}$$

Because $m_{2,+1} + 1 = m_{1,+1} = m_{max} + 1$, the upper bound on the $\bar{r}$ summation is the same in (197) and (198). Similarly

$$h^1_{[1m0],[2r']} c^\dagger_{11k0ms} f_{21kr's} = i\sqrt{2}\sqrt{m}\frac{v'_*}{\ell} \sum_{m=0}^{m_{1,+1}} \sum_{\bar{r}=0}^{m_{1,+1}-1} \Sigma_{\bar{r}} \delta_{m-1,\bar{r}} c^\dagger_{11k0ms} \bar{f}_{21k\bar{r}s}, \tag{199}$$

$$h^1_{[2m0],[2r']} c^\dagger_{21k0ms} f_{21kr's} = \gamma \sum_{m=0}^{m_{2,+1}} \sum_{\bar{r}=0}^{m_{2,+1}} \Sigma_m \delta_{m,\bar{r}} c^\dagger_{21k0ms} \bar{f}_{21k\bar{r}s}. \tag{200}$$

Because $m_{1,+1} - 1 = m_{2,+1} = m_{max}$, the upper bound on $\bar{r}$ summation is the same in Eq.(199) and Eq.(200). Thus for a given k, out of the available $2q$ $f-$modes, only $2m_{max} + 3$ couple; recall that $m_{max} \lesssim q/2$.

A. CNP

The mean-field interactions for f-fermion modes at CNP in $\bar{f}$ basis is given as

$$V^{f,\tau=+1,s} = \sum_{k \in [0,1) \otimes [0,\frac{1}{q})} V^{f,\tau=+1,s}_{coupled} + V^{f,\tau=+1,s}_{decoupled}, \tag{201}$$

$$V^{f,\tau=+1,s}_{coupled} = -\frac{U_1}{2} \left(\sum_{m=0}^{m_{max}+1} \bar{f}^\dagger_{11kms} \bar{f}_{11kms} + \sum_{m=0}^{m_{max}} \bar{f}^\dagger_{21kms} \bar{f}_{21kms} \right) \tag{202}$$

$$V^{f,\tau=+1,s}_{decoupled} = -\frac{U_1}{2} \sum_{b=1}^{2} \sum_{m'=m_{max}+\bar{b}}^{q-1} \bar{f}^\dagger_{b1km's} \bar{f}_{b1km's}, \tag{203}$$

where $\bar{1}(\bar{2}) = 2(1)$. Note that there are $2q - (2m_{max} + 3)$ decoupled f modes for each k. The coupled modes
The coupled modes can then be described as

$$H^{\tau=+1,s}_{coupled} = \sum_k \sum_{\alpha,\alpha'=1}^{6} \sum_{m=0}^{m_\alpha} \sum_{m'=0}^{m_{\alpha'}} \Xi_{m\alpha,m'\alpha'} d^\dagger_{m\alpha s}(k) d_{m'\alpha' s}(k), \tag{204}$$

where $m_{\alpha=1,\dots,4} = m_{\alpha,+1}$, $m_5 = m_{max}+1$ and $m_6 = m_{max}$, and

$$d^{\dagger}_{m\alpha s}(k) = \left(c^{\dagger}_{11k0ms}, c^{\dagger}_{21k0ms}, c^{\dagger}_{31k0ms}, c^{\dagger}_{41k0ms}, f^{\dagger}_{11kms}, f^{\dagger}_{21kms}\right)_{\alpha}. \tag{205}$$

We now define an operator

$$\hat{h}^{+1}_{\alpha,\alpha'} = \begin{pmatrix} 0 & 0 & -i\sqrt{2}\frac{v_*}{\ell}\hat{a} & 0 & \gamma\Sigma(\hat{a}^{\dagger}\hat{a}) & i\sqrt{2}\frac{v'_*}{\ell}\hat{a}^{\dagger}\Sigma(\hat{a}^{\dagger}\hat{a}) \\ 0 & 0 & 0 & i\sqrt{2}\frac{v_*}{\ell}\hat{a}^{\dagger} & -i\sqrt{2}\frac{v'_*}{\ell}\hat{a}\Sigma(\hat{a}^{\dagger}\hat{a}) & \gamma\Sigma(\hat{a}^{\dagger}\hat{a}) \\ i\sqrt{2}\frac{v_*}{\ell}\hat{a}^{\dagger} & 0 & -\frac{J}{2} & M & 0 & 0 \\ 0 & -i\sqrt{2}\frac{v_*}{\ell}\hat{a} & M & -\frac{J}{2} & 0 & 0 \\ \gamma\Sigma(\hat{a}^{\dagger}\hat{a}) & i\sqrt{2}\frac{v'_*}{\ell}\Sigma(\hat{a}^{\dagger}\hat{a})\hat{a}^{\dagger} & 0 & 0 & -\frac{U_1}{2} & 0 \\ -i\sqrt{2}\frac{v'_*}{\ell}\Sigma(\hat{a}^{\dagger}\hat{a})\hat{a} & \gamma\Sigma(\hat{a}^{\dagger}\hat{a}) & 0 & 0 & 0 & -\frac{U_1}{2} \end{pmatrix}_{\alpha,\alpha'}, \tag{206}$$

where $\hat{a}$ is a simple h.o. lowering operator in terms of which matrix $\Xi_{m\alpha,m'\alpha'}$ can be expressed as

$$\Xi_{m\alpha,m'\alpha'} = \langle m|\hat{h}^{+1}_{\alpha,\alpha'}|m'\rangle. \tag{207}$$

Here $|m\rangle$ is a simple h.o. eigenstate and $\Sigma(m) = \Sigma_m$. The non-interacting Hofstadter spectrum can be obtained by solving Eq.(206) after setting the mean field terms J, U_1 to zero. The spectrum for $M \neq 0$ including the $2q-(2m_{max}+3$ zero modes (contributed by decoupled fs) is shown in Fig.(10) with $2q$ modes at each k, i.e. 2 modes per moiré unit cell per valley for each spin projection.

We now discuss the exact solutions to the eigenstates of the operator in Eq.(206) in the flat band limit $M=0$. The **B** field independent $-J/2$ Landau level energy shown in main-text Fig.(1) comes from the anomalous c-mode

$$\theta_1 = [0,0,|0\rangle,0,0,0]^T. \tag{208}$$

The rest of the problem can be solved using the following ansätze:

$$\theta_3 = \left[c^{(3)}_1|0\rangle, 0, c^{(3)}_3|1\rangle, 0, c^{(3)}_5|0\rangle, 0\right]^T, \tag{209}$$

$$\theta_5 = \left[c^{(5)}_1|1\rangle, c^{(5)}_2|0\rangle, c^{(5)}_3|2\rangle, 0, c^{(5)}_5|1\rangle, c^{(5)}_6|0\rangle\right]^T, \tag{210}$$

$$\theta_{6_m} = \left[c^{(6_m)}_1|m\rangle, c^{(6_m)}_2|m-1\rangle, c^{(6_m)}_3|m+1\rangle, c^{(6_m)}_4|m-2\rangle, c^{(6_m)}_5|m\rangle, c^{(6_m)}_6|m-1\rangle\right]^T, \tag{211}$$

where $m \in \{2,\dots,m_{max}+1\}$. $c^{(\beta)}_{\alpha}$ denotes the coefficient of corresponding h.o state at index α in the 6-component spinor in Eq.(205) and β labels the ansatz index θ_{β}. Using the above, we can set up the eigen-equation and solve for the corresponding coefficients.

The ansätze θ_3 and θ_5 yield the 3×3 and 5×5 Hermitian matrices, whose eigenvectors are $c^{(3)}_{\alpha}$ and $c^{(5)}_{\alpha}$, respectively:

$$h^{+1}_3 = \begin{pmatrix} 0 & -i\frac{\sqrt{2}v_*}{\ell} & \gamma\Sigma_0 \\ i\frac{\sqrt{2}v_*}{\ell} & -\frac{J}{2} & 0 \\ \gamma\Sigma_0 & 0 & -\frac{U_1}{2} \end{pmatrix}, \tag{212}$$

$$h^{+1}_5 = \begin{pmatrix} 0 & 0 & -i\frac{2v_*}{\ell} & \gamma\Sigma_1 & i\frac{\sqrt{2}v'_*}{\ell}\Sigma_0 \\ 0 & 0 & 0 & -i\frac{\sqrt{2}v'_*}{\ell}\Sigma_1 & \gamma\Sigma_0 \\ i\frac{2v_*}{\ell} & 0 & -\frac{J}{2} & 0 & 0 \\ \gamma\Sigma_1 & i\frac{\sqrt{2}v'_*}{\ell}\Sigma_1 & 0 & -\frac{U_1}{2} & 0 \\ -i\frac{\sqrt{2}v'_*}{\ell}\Sigma_0 & \gamma\Sigma_0 & 0 & 0 & -\frac{U_1}{2} \end{pmatrix}. \tag{213}$$

Similarly, the ansatz θ^m_6 yields the following 6×6 Hermitian matrix for each m, whose eigenvectors are $c^{(6,m)}_{\alpha}$:

$$h^{+1,m}_6 = \begin{pmatrix} 0 & 0 & -i\sqrt{2m+2}\frac{v_*}{\ell} & 0 & \gamma\Sigma_m & i\sqrt{2m}\frac{v'_*}{\ell}\Sigma_{m-1} \\ 0 & 0 & 0 & i\sqrt{2m-2}\frac{v_*}{\ell} & -i\sqrt{2m}\frac{v'_*}{\ell}\Sigma_m & \gamma\Sigma_{m-1} \\ +i\sqrt{2m+2}\frac{v_*}{\ell} & 0 & -\frac{J}{2} & 0 & 0 & 0 \\ 0 & -i\sqrt{2m-2}\frac{v_*}{\ell} & 0 & -\frac{J}{2} & 0 & 0 \\ \gamma\Sigma_m & i\sqrt{2m}\frac{v'_*}{\ell}\Sigma_m & 0 & 0 & -\frac{U_1}{2} & 0 \\ -i\sqrt{2m}\frac{v'_*}{\ell}\Sigma_{m-1} & \gamma\Sigma_{m-1} & 0 & 0 & 0 & -\frac{U_1}{2} \end{pmatrix}. \tag{214}$$

The magnetic subbands within the narrow bands from the coupled modes emanate out of the $\mathbf{B} \to 0$ energy eigenvalue of the above decoupled matrices, $-\frac{J}{2}$, which is 2 fold degenerate for matrix in Eq.(214)$\forall m$ and singly degenerate for matrices in Eq.(212) and Eq.(213). Including the decoupled c mode, we have $2m_{max}+3$ magnetic modes emanating out of this $2m_{max}+3$ fold degenerate $\mathbf{B} \to 0$ energy eigenvalue. Now recall that we have $2q-(2m_{max}+3)$ decoupled f modes with energy $-\frac{U_1}{2}$. Thus in total we have $2q$ magnetic modes within the narrow bands, which corresponds to 2 states per moiré unit cell per spin. The spectrum for flat band limit has been shown in the main text Fig.(2). Note that the $\mathbf{B} \to 0$ energies recovered by the decoupled matrices are the corresponding zero field energies of THFM at Γ in mBZ.

B. $\nu = \pm 1$, Spin $\uparrow$

Here we discuss the finite $\mathbf{B}$ THFM for valley $\mathbf{K}$ spin $\uparrow$. The interactions read as [2]

$$V^{\tau=+1,s=\uparrow}_{\nu=\pm1} = \nu \sum_k \Bigg(\sum_{a=1}^{4} \sum_{m=0}^{m_{a,+1}} \sum_{r=0}^{p-1} W_a c^\dagger_{a1krm\uparrow} c_{a1krm\uparrow} + \sum_{a=3,4} \sum_{m=0}^{m_{a,-1}} \sum_{r=0}^{p-1} \frac{J}{2} c^\dagger_{a1krm\uparrow} c_{a1krm\uparrow}$$
$$+ \sum_{b=1,2} \sum_{r'=0}^{q-1} \left(\frac{3U_1}{2} + 6U_2\right) f^\dagger_{b1kr'\uparrow} f_{b1kr'\uparrow} \Bigg). \tag{215}$$

The interaction for f modes in the $\bar{f}$ basis can then be given as

$$V^{f,\tau=+1,\uparrow}_{\nu=\pm1} = \sum_{k\in[0,1)\otimes[0,\frac{1}{q})} V^{f,\tau=+1,\uparrow,\nu=\pm1}_{coupled} + V^{f,\tau=+1,\uparrow,\nu=\pm1}_{decoupled} \tag{216}$$

where

$$V^{f,\tau=+1,\uparrow,\nu=\pm1}_{coupled} = \nu \left(\frac{3U_1}{2} + 6U_2\right) \left(\sum_{m=0}^{m_{max}+1} \bar{f}^\dagger_{11kms} \bar{f}_{11km\uparrow} + \sum_{m=0}^{m_{max}} \bar{f}^\dagger_{21km\uparrow} \bar{f}_{21km\uparrow} \right), \tag{217}$$

$$V^{f,\tau=+1,s,\nu=\pm1}_{decoupled} = \nu \sum_{b=1}^{2} \sum_{m'=m_{max}+\bar{b}}^{q-1} \left(\frac{3U_1}{2} + 6U_2\right) \bar{f}^\dagger_{b1km'\uparrow} \bar{f}_{b1km'\uparrow}. \tag{218}$$

where $\bar{1}(\bar{2}) = 2(1)$. Yet again there are $2q-(2m_{max}+3)$ decoupled f modes for each k. Physically this corresponds to $2-(2m_{max}+3)/q$ per moiré unit cell. The coupled modes can then be described by

$$H^{\tau=+1,\uparrow,\nu=\pm1}_{coupled} = \sum_k \sum_{\alpha,\alpha'=1}^{6} \sum_{m=0}^{m_\alpha} \sum_{m'=0}^{m_{\alpha'}} \Xi^{\uparrow,\nu=\pm1}_{m\alpha,m'\alpha'} d^\dagger_{m\alpha\uparrow}(k) d_{m'\alpha'\uparrow}(k), \tag{219}$$

where $m_{\alpha=1,\dots,4} = m_{\alpha,+1}$, $m_5 = m_{max}+1$ and $m_6 = m_{max}$, and

$$d^\dagger_{m\alpha\uparrow}(k) = \left(c^\dagger_{11k0m\uparrow}, c^\dagger_{21k0m\uparrow}, c^\dagger_{31k0m\uparrow}, c^\dagger_{41k0m\uparrow}, \bar{f}^\dagger_{11km\uparrow}, \bar{f}^\dagger_{21km\uparrow} \right)_\alpha, \tag{220}$$

with

$$\Xi^{\uparrow,\nu=\pm1}_{m\alpha,m'\alpha'} = \langle m | \hat{h}^{+1,\uparrow,\nu=\pm1}_{\alpha,\alpha'} | m' \rangle, \tag{221}$$

where the operators $\hat{h}^{+1,\uparrow,\nu=\pm1}_{\alpha,\alpha'}$ are given as

$$\hat{h}^{+1,\uparrow,\nu=\pm1}_{\alpha,\alpha'} = \begin{pmatrix} \nu W_1 & 0 & -i\sqrt{2}\frac{v_*}{\ell}\hat{a} & 0 & \gamma\Sigma(\hat{a}^\dagger\hat{a}) & i\sqrt{2}\frac{v'_*}{\ell}\hat{a}^\dagger\Sigma(\hat{a}^\dagger\hat{a}) \\ 0 & \nu W_1 & 0 & i\sqrt{2}\frac{v_*}{\ell}\hat{a}^\dagger & -i\sqrt{2}\frac{v'_*}{\ell}\hat{a}\Sigma(\hat{a}^\dagger\hat{a}) & \gamma\Sigma(\hat{a}^\dagger\hat{a}) \\ i\sqrt{2}\frac{v_*}{\ell}\hat{a}^\dagger & 0 & \nu(W_3+\frac{J}{2}) & M & 0 & 0 \\ 0 & -i\sqrt{2}\frac{v_*}{\ell}\hat{a} & M & \nu(W_3+\frac{J}{2}) & 0 & 0 \\ \gamma\Sigma(\hat{a}^\dagger\hat{a}) & i\sqrt{2}\frac{v'_*}{\ell}\Sigma(\hat{a}^\dagger\hat{a})\hat{a}^\dagger & 0 & 0 & \nu(\frac{3U_1}{2}+6U_2) & 0 \\ -i\sqrt{2}\frac{v'_*}{\ell}\Sigma(\hat{a}^\dagger\hat{a})\hat{a} & \gamma\Sigma(\hat{a}^\dagger\hat{a}) & 0 & 0 & 0 & \nu(\frac{3U_1}{2}+6U_2) \end{pmatrix}_{\alpha,\alpha'} \tag{222}$$

where $\hat{a}$ is a simple h.o. lowering operator with $|m\rangle$ being a simple h.o. eigenstate and $\Sigma(m) = \Sigma_m$. The exact eigenstates for the above operator are exactly solvable in flat band limit, $M = 0$. The $\mathbf{B}$ field independent level

$\nu(W_3+J/2)$ is formed by the anomalous c-mode in Eq.(208). The rest of the problem can be solved using the ansätze: Eq.(209)-Eq.(211). The corresponding coefficients $c_\alpha^{(3)}$, $c_\alpha^{(5)}$ and $c_\alpha^{(6,m)}$ can thus be solved as eigenvectors of the following 3×3, 5×5 and m_{max} 6×6 Hermitian matrices respectively:

$$h_3^{+1,\nu=\pm1} = \begin{pmatrix} \nu W_1 & -i\frac{\sqrt{2}v_*}{\ell} & \gamma\Sigma_0 \\ i\frac{\sqrt{2}v_*}{\ell} & \nu(W_3+\frac{J}{2}) & 0 \\ \gamma\Sigma_0 & 0 & \nu(\frac{3}{2}U_1+6U_2) \end{pmatrix}, \tag{223}$$

$$h_5^{+1,\nu=\pm1} = \begin{pmatrix} \nu W_1 & 0 & -i\frac{2v_*}{\ell} & \gamma\Sigma_1 & i\frac{\sqrt{2}v_*'}{\ell}\Sigma_0 \\ 0 & \nu W_1 & 0 & -i\frac{\sqrt{2}v_*'}{\ell}\Sigma_1 & \gamma\Sigma_0 \\ i\frac{2v_*}{\ell} & 0 & \nu(W_3+\frac{J}{2}) & 0 & 0 \\ \gamma\Sigma_1 & i\frac{\sqrt{2}v_*'}{\ell}\Sigma_1 & 0 & \nu(\frac{3U_1}{2}+6U_2) & 0 \\ -i\frac{\sqrt{2}v_*'}{\ell}\Sigma_0 & \gamma\Sigma_0 & 0 & 0 & \nu(\frac{3U_1}{2}+6U_2) \end{pmatrix}, \tag{224}$$

$$h_6^{+1,m,\nu=\pm1} = \begin{pmatrix} \nu W_1 & 0 & -i\sqrt{2m+2}\frac{v_*}{\ell} & 0 & \gamma\Sigma_m & i\sqrt{2m}\frac{v_*'}{\ell}\Sigma_{m-1} \\ 0 & \nu W1 & 0 & i\sqrt{2m-2}\frac{v_*}{\ell} & -i\sqrt{2m}\frac{v_*'}{\ell}\Sigma_m & \gamma\Sigma_{m-1} \\ +i\sqrt{2m+2}\frac{v_*}{\ell} & 0 & \nu(W_3+\frac{J}{2}) & 0 & 0 & 0 \\ 0 & -i\sqrt{2m-2}\frac{v_*}{\ell} & 0 & \nu(W_3+\frac{J}{2}) & 0 & 0 \\ \gamma\Sigma_m & i\sqrt{2m}\frac{v_*'}{\ell}\Sigma_m & 0 & 0 & \nu(\frac{3U_1}{2}+6U_2) & 0 \\ -i\sqrt{2m}\frac{v_*'}{\ell}\Sigma_{m-1} & \gamma\Sigma_{m-1} & 0 & 0 & 0 & \nu(\frac{3U_1}{2}+6U_2) \end{pmatrix} \tag{225}$$

The magnetic subbands within the narrow bands from the coupled modes emanate out of the $\mathbf{B}\to0$ energy eigenvalue of the above decoupled matrices, $\nu(W_3+\frac{J}{2})$, which is 2 fold degenerate for matrix in Eq.(225)$\forall m$ and singly degenerate for matrices in Eq.(223) and Eq.(224). Including the decoupled c mode, we have $2m_{max}+3$ magnetic modes emanating out of this $\mathbf{B}\to0$ energy eigenvalue. Now recall that we have $2q-(2m_{max}+3)$ decoupled f modes with energy $\nu(\frac{3U_1}{2}+6U_2)$. Thus in total we have $2q$ magnetic modes within the narrow bands, which corresponds to 2 states per moiré unit cell. The spectrum for flat band limit has been shown in Supplementary Fig.(8b). Note that the $\mathbf{B}\to0$ energies recovered by the decoupled matrices are the corresponding zero field energies of THFM at Γ in mBZ.

C. $\nu=\pm1$, Spin $\downarrow$

Here we discuss the finite $\mathbf{B}$ THFM at $\nu=\pm1$ for $\tau=+1$ and spin $\downarrow$. The interactions read as[2]

$$V_{\nu=\pm1}^{\tau=+1,\downarrow} = \nu\sum_k\Bigg(\sum_{a=1}^{4}\sum_{m=0}^{m_{a,+1}}\sum_{r=0}^{p-1} W_a c^\dagger_{a1krm\downarrow}c_{a1krm\downarrow} + \sum_{a=3,4}\sum_{m=0}^{m_{a,+1}}\sum_{r=0}^{p-1}(-1)^{a+1}\frac{J}{2}c^\dagger_{a1krm\downarrow}c_{a1krm\downarrow}$$
$$+\sum_{b=1,2}\sum_{r'=0}^{q-1}\left(\frac{2+(-1)^{b+1}}{2}U_1+6U_2\right)f^\dagger_{b1kr'\downarrow}f_{b1kr'\downarrow}\Bigg). \tag{226}$$

Here $W_{a\in\{1\ldots4\}}$ and U_2 are mean field coefficients with $W_1=W_2$ and $W_3=W_4$ [2]. In the $\bar{f}$ basis we can re-write the interaction for f-fermion modes as

$$V_{\nu=\pm1}^{f,\tau=+1,\downarrow} = \sum_{k\in[0,1)\otimes[0,\frac{1}{q})} V_{coupled}^{f,\tau=+1,\downarrow,\nu=\pm1} + V_{decoupled}^{f,\tau=+1,\downarrow,\nu=\pm1} \tag{227}$$

where

$$V_{coupled}^{f,\tau=+1,\downarrow,\nu=\pm1} = \nu\left(\frac{3}{2}U_1+6U_2\right)\left(\sum_{m=0}^{m_{max}+1}\bar{f}^\dagger_{11km\downarrow}\bar{f}_{11km\downarrow}\right)+\nu\left(\frac{1}{2}U_1+6U_2\right)\left(\sum_{m=0}^{m_{max}}\bar{f}^\dagger_{21kms}\bar{f}_{21km\downarrow}\right), \tag{228}$$

$$V_{decoupled}^{f,\tau=+1,s,\nu=\pm2} = \nu\sum_{b=1}^{2}\sum_{m'=m_{max}+\bar{b}}^{q-1}\left(\frac{2+(-1)^{b+1}}{2}U_1+6U_2\right)\bar{f}^\dagger_{b1km'\downarrow}\bar{f}_{b1km'\downarrow}, \tag{229}$$

where $\bar{1}(\bar{2}) = 2, 1$. Note that out of the available $2q$ f modes, $q-(m_{max}+2)$ are decoupled with energy $\nu\left(\frac{3}{2}U_1 + 6U_2\right)$ and $q-(m_{max}+1)$ are decoupled with energy $\nu\left(\frac{1}{2}U_1 + 6U_2\right)$, i.e. a total of $2-(2m_{max}+3)/q$ decoupled f modes per moiré unit cell. The coupled modes can then be described by

$$H^{\tau=+1,\downarrow,\nu=\pm1}_{coupled} = \sum_k \sum_{\alpha,\alpha'=1}^{6} \sum_{m=0}^{m_\alpha} \sum_{m'=0}^{m_{\alpha'}} \Xi^{\downarrow,\nu=\pm1}_{m\alpha,m'\alpha'} d^\dagger_{m\alpha\downarrow}(k) d_{m'\alpha'\downarrow}(k), \tag{230}$$

where $m_{\alpha=1,\dots,4} = m_{\alpha,+1}$, $m_5 = m_{max}+1$ and $m_6 = m_{max}$, and

$$d^\dagger_{m\alpha\downarrow}(k) = \left(c^\dagger_{11k0m\downarrow}, c^\dagger_{21k0m\downarrow}, c^\dagger_{31k0m\downarrow}, c^\dagger_{41k0m\downarrow}, \bar{f}^\dagger_{11km\downarrow}, \bar{f}^\dagger_{21km\downarrow}\right)_\alpha, \tag{231}$$

with

$$\Xi^{\downarrow,\nu=\pm1}_{m\alpha,m'\alpha'} = \langle m|\hat{h}^{+1,\downarrow,\nu=\pm1}_{\alpha,\alpha'}|m'\rangle, \tag{232}$$

where the operators $\hat{h}^{+1,\downarrow,\nu=\pm1}_{\alpha,\alpha'}$ are given as

$$\hat{h}^{+1,\downarrow,\nu=\pm1}_{\alpha,\alpha'} = \begin{pmatrix} \nu W_1 & 0 & -i\sqrt{2}\frac{v_*}{\ell}\hat{a} & 0 & \gamma\Sigma(\hat{a}^\dagger\hat{a}) & i\sqrt{2}\frac{v'_*}{\ell}\hat{a}^\dagger\Sigma(\hat{a}^\dagger\hat{a}) \\ 0 & \nu W_1 & 0 & i\sqrt{2}\frac{v_*}{\ell}\hat{a}^\dagger & -i\sqrt{2}\frac{v'_*}{\ell}\hat{a}\Sigma(\hat{a}^\dagger\hat{a}) & \gamma\Sigma(\hat{a}^\dagger\hat{a}) \\ i\sqrt{2}\frac{v_*}{\ell}\hat{a}^\dagger & 0 & \nu(W_3+\frac{J}{2}) & M & 0 & 0 \\ 0 & -i\sqrt{2}\frac{v_*}{\ell}\hat{a} & M & \nu(W_3-\frac{J}{2}) & 0 & 0 \\ \gamma\Sigma(\hat{a}^\dagger\hat{a}) & i\sqrt{2}\frac{v'_*}{\ell}\Sigma(\hat{a}^\dagger\hat{a})\hat{a}^\dagger & 0 & 0 & \nu(\frac{3}{2}U_1+6U_2) & 0 \\ -i\sqrt{2}\frac{v'_*}{\ell}\Sigma(\hat{a}^\dagger\hat{a})\hat{a} & \gamma\Sigma(\hat{a}^\dagger\hat{a}) & 0 & 0 & 0 & \nu(\frac{1}{2}U_1+6U_2) \end{pmatrix}_{\alpha,\alpha'} \tag{233}$$

The eigenstates of the above operator are exactly solvable for the flat band limit, i.e. $M = 0$. The decoupled c fermion given in Eq.(208) forms the field independent $\nu(W_3+\frac{J}{2})$ level. The remaining eigenstates can be obtained using the ansätze given in Eqs.(209)-(211). Setting up eigen-equation for these ansätze yield us the following 3×3, 5×5 and m_{max} 6×6 matrices:

$$h^{+1,\nu=\pm1}_3 = \begin{pmatrix} \nu W_1 & -i\frac{\sqrt{2}v_*}{\ell} & \gamma\Sigma_0 \\ i\frac{\sqrt{2}v_*}{\ell} & \nu(W_3+\frac{J}{2}) & 0 \\ \gamma\Sigma_0 & 0 & \nu(\frac{3}{2}U_1+6U_2) \end{pmatrix}, \tag{234}$$

$$h^{+1,\nu=\pm1}_5 = \begin{pmatrix} \nu W_1 & 0 & -i\frac{2v_*}{\ell} & \gamma\Sigma_1 & i\frac{\sqrt{2}v'_*}{\ell}\Sigma_0 \\ 0 & \nu W_1 & 0 & -i\frac{\sqrt{2}v'_*}{\ell}\Sigma_1 & \gamma\Sigma_0 \\ i\frac{2v_*}{\ell} & 0 & \nu(W_3+\frac{J}{2}) & 0 & 0 \\ \gamma\Sigma_1 & i\frac{\sqrt{2}v'_*}{\ell}\Sigma_1 & 0 & \nu(\frac{3U_1}{2}+6U_2) & 0 \\ -i\frac{\sqrt{2}v'_*}{\ell}\Sigma_0 & \gamma\Sigma_0 & 0 & 0 & \nu(\frac{U_1}{2}+6U_2) \end{pmatrix}, \tag{235}$$

$$h^{+1,m,\nu=\pm1}_6 = \begin{pmatrix} \nu W_1 & 0 & -i\sqrt{2m+2}\frac{v_*}{\ell} & 0 & \gamma\Sigma_m & i\sqrt{2m}\frac{v'_*}{\ell}\Sigma_{m-1} \\ 0 & \nu W1 & 0 & i\sqrt{2m-2}\frac{v_*}{\ell} & -i\sqrt{2m}\frac{v'_*}{\ell}\Sigma_m & \gamma\Sigma_{m-1} \\ +i\sqrt{2m+2}\frac{v_*}{\ell} & 0 & \nu(W_3+\frac{J}{2}) & 0 & 0 & 0 \\ 0 & -i\sqrt{2m-2}\frac{v_*}{\ell} & 0 & \nu(W_3-\frac{J}{2}) & 0 & 0 \\ \gamma\Sigma_m & i\sqrt{2m}\frac{v'_*}{\ell}\Sigma_m & 0 & 0 & \nu(\frac{3U_1}{2}+6U_2) & 0 \\ -i\sqrt{2m}\frac{v'_*}{\ell}\Sigma_{m-1} & \gamma\Sigma_{m-1} & 0 & 0 & 0 & \nu(\frac{U_1}{2}+6U_2) \end{pmatrix}, \tag{236}$$

where $m \in \{2\dots m_{max}+1\}$. The magnetic subbands within the narrow bands from the coupled modes emanate out of the $\mathbf{B}\to0$ energy eigenvalue of the above decoupled matrices, $\nu(W_3\pm\frac{J}{2})$. $\nu(W_3+\frac{J}{2})$ here is singly degenerate for each of the above matrices and thus in total $m_{max}+2$ fold degenerate. On the other hand $\nu(W_3-\frac{J}{2})$ is in total m_{max} fold degenerate for the matrix in Eq.(236) including all values of m. Including the anomalous c mode and $2q-(2m_{max}+3)$ decoupled f modes, we have a total of $2q$ magnetic modes within the narrow bands, which corresponds to 2 states per moiré unit cell per spin. The spectrum for flat band limit has been shown in the main text Fig.(5-b). Note that the $\mathbf{B}\to0$ energies recovered by the decoupled matrices are the corresponding zero field energies of THFM at Γ in mBZ.

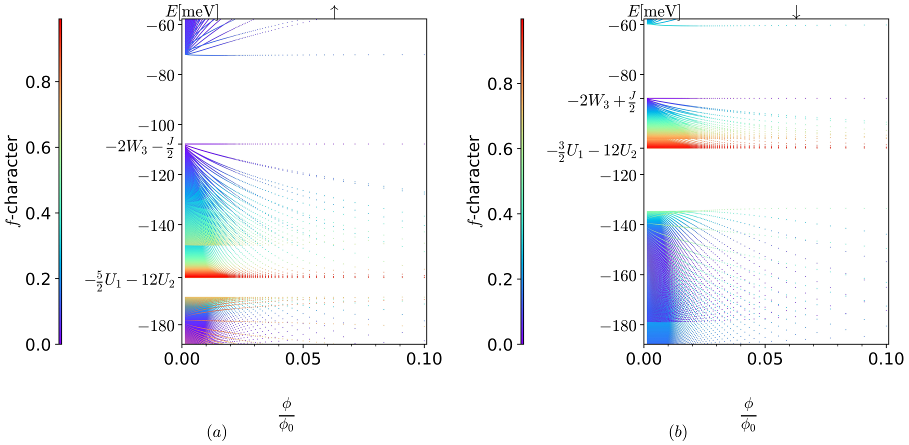

Supplementary Figure 6. Interacting heavy fermion Hofstadter spectra for valley **K** (a) spin ↑ and (b) spin ↓ at filling $\nu=-2$ at $w_0/w_1=0.7$ in the flat band limit $M=0$. $m_{max}=\lceil\frac{q-3}{2}\rceil$.

D. $\nu=\pm2$, Spin ↑↓

In this section we discuss the finite **B** THFM at $\nu=\pm2$, Valley **K** and spin ↑↓ sectors. The mean field interaction at filling $\nu=\pm2$ for VP state at $\tau=+1$ for spin sector s reads[2]

$$V^{\tau=+1,s}_{\nu=\pm2}=\nu\sum_k\left(\sum_{a=1}^{4}\sum_{m=0}^{m_{a,+1}}\sum_{r=0}^{p-1}W_a c^\dagger_{a1krms}c_{a1krms}+\sigma_s\sum_{a=3,4}\sum_{m=0}^{m_{a,+1}}\sum_{r=0}^{p-1}\frac{J}{4}c^\dagger_{a1krms}c_{a1krms}\right.$$
$$\left.+\sum_{b=1,2}\sum_{r'=0}^{q-1}\left(\frac{4+\sigma_s}{4}U_1+6U_2\right)f^\dagger_{b1kr's}f_{b1kr's}\right),\tag{237}$$

where $\sigma_s=\pm1$ for $s=$↑↓ respectively. In the $\bar f$ basis we can re-write the interaction for f-fermion modes as

$$V^{f,\tau=+1,s}_{\nu=\pm2}=\sum_{k\in[0,1)\otimes[0,\frac{1}{q})}V^{f,\tau=+1,s,\nu=\pm2}_{coupled}+V^{f,\tau=+1,s,\nu=\pm2}_{decoupled}\tag{238}$$

where

$$V^{f,\tau=+1,s,\nu=\pm2}_{coupled}=\nu\left(\frac{4+\sigma_s}{4}U_1+6U_2\right)\left(\sum_{m=0}^{m_{max}+1}\bar f^\dagger_{11kms}\bar f_{11kms}+\sum_{m=0}^{m_{max}}\bar f^\dagger_{21kms}\bar f_{21kms}\right),\tag{239}$$

$$V^{f,\tau=+1,s,\nu=\pm2}_{decoupled}=\nu\left(\frac{4+\sigma_s}{4}U_1+6U_2\right)\sum_{b=1}^{2}\sum_{m'=m_{max}+\bar b}^{q-1}\bar f^\dagger_{b1km's}\bar f_{b1km's},\tag{240}$$

where $\bar 1(\bar 2)=2,1$. Note that we have $2-(2m_{max}+3)/q$ decoupled f states per moiré unit cell for each spin projection. The coupled modes can then be described by

$$H^{\tau=+1,s,\nu=\pm2}_{coupled}=\sum_k\sum_{\alpha,\alpha'=1}^{6}\sum_{m=0}^{m_\alpha}\sum_{m'=0}^{m_{\alpha'}}\Xi^{s,\nu=\pm2}_{m\alpha,m'\alpha'}d^\dagger_{m\alpha s}(k)d_{m'\alpha's}(k),\tag{241}$$

where $m_{\alpha=1,\ldots,4}=m_{\alpha,+1}$, $m_5=m_{max}+1$ and $m_6=m_{max}$, and

$$d^\dagger_{m\alpha s}(k)=\left(c^\dagger_{11k0ms},c^\dagger_{21k0ms},c^\dagger_{31k0ms},c^\dagger_{41k0ms},\bar f^\dagger_{11kms},\bar f^\dagger_{21kms}\right)_\alpha,\tag{242}$$

with

$$\Xi^{s,\nu=\pm2}_{m\alpha,m'\alpha'}=\langle m|\hat h^{+1,s,\nu=\pm2}_{\alpha,\alpha'}|m'\rangle,\tag{243}$$

where the operators $\hat{h}^{+1,s,\nu=\pm2}_{\alpha,\alpha'}$ are given as

$$\hat{h}^{+1,s,\nu=\pm2}_{\alpha,\alpha'} = \begin{pmatrix} \nu W_1 & 0 & -i\sqrt{2}\frac{v_*}{\ell}\hat{a} & 0 & \gamma\Sigma(\hat{a}^\dagger\hat{a}) & i\sqrt{2}\frac{v'_*}{\ell}\hat{a}^\dagger\Sigma(\hat{a}^\dagger\hat{a}) \\ 0 & \nu W_1 & 0 & i\sqrt{2}\frac{v_*}{\ell}\hat{a}^\dagger & -i\sqrt{2}\frac{v'_*}{\ell}\hat{a}\Sigma(\hat{a}^\dagger\hat{a}) & \gamma\Sigma(\hat{a}^\dagger\hat{a}) \\ i\sqrt{2}\frac{v_*}{\ell}\hat{a}^\dagger & 0 & \nu(W_3+\sigma_s\frac{J}{4}) & M & 0 & 0 \\ 0 & -i\sqrt{2}\frac{v_*}{\ell}\hat{a} & M & \nu(W_3+\sigma_s\frac{J}{4}) & 0 & 0 \\ \gamma\Sigma(\hat{a}^\dagger\hat{a}) & i\sqrt{2}\frac{v'_*}{\ell}\Sigma(\hat{a}^\dagger\hat{a})\hat{a}^\dagger & 0 & 0 & \nu(\frac{4+\sigma_s}{4}U_1+6U_2) & 0 \\ -i\sqrt{2}\frac{v'_*}{\ell}\Sigma(\hat{a}^\dagger\hat{a})\hat{a} & \gamma\Sigma(\hat{a}^\dagger\hat{a}) & 0 & 0 & 0 & \nu(\frac{4+\sigma_s}{4}U_1+6U_2) \end{pmatrix}_{\alpha,\alpha'} \tag{244}$$

where $\hat{a}$ is a simple h.o. lowering operator, $|m\rangle$ is a simple h.o. eigenstate and $\Sigma(m) = \Sigma_m$. The exact eigenstates for the above operator are exactly solvable in flat band limit, $M = 0$. The field independent $\pm(2W_3 + \frac{J}{2})$ and $\pm(2W_3 - \frac{J}{2})$ levels at fillings $\nu = \pm2$ and $\tau = +1$ for spin $\uparrow\downarrow$ sectors respectively is formed by the anomalous c-mode in Eq.(208). The rest of the problem can be solved using the ansätze: Eq.(209)-Eq.(211). The corresponding coefficients $c^{(3)}_\alpha$, $c^{(5)}_\alpha$ and $c^{(6,m)}_\alpha$ can thus be solved as eigenvectors of the following 3×3, 5×5 and m_{max} 6×6 Hermitian matrices respectively:

$$h^{+1,s,\nu=\pm2}_3 = \begin{pmatrix} \nu W_1 & -i\frac{\sqrt{2}v_*}{\ell} & \gamma\Sigma_0 \\ i\frac{\sqrt{2}v_*}{\ell} & \nu(W_3+\sigma_s\frac{J}{4}) & 0 \\ \gamma\Sigma_0 & 0 & \nu(\frac{4+\sigma_s}{4}U_1+6U_2) \end{pmatrix}, \tag{245}$$

$$h^{+1,s,\nu=\pm2}_5 = \begin{pmatrix} \nu W_1 & 0 & -i\frac{2v_*}{\ell} & \gamma\Sigma_1 & i\frac{\sqrt{2}v'_*}{\ell}\Sigma_0 \\ 0 & \nu W_1 & 0 & -i\frac{\sqrt{2}v'_*}{\ell}\Sigma_1 & \gamma\Sigma_0 \\ i\frac{2v_*}{\ell} & 0 & \nu(W_3+\sigma_s\frac{J}{4}) & 0 & 0 \\ \gamma\Sigma_1 & i\frac{\sqrt{2}v'_*}{\ell}\Sigma_1 & 0 & \nu(\frac{4+\sigma_s}{4}U_1+6U_2) & 0 \\ -i\frac{\sqrt{2}v'_*}{\ell}\Sigma_0 & \gamma\Sigma_0 & 0 & 0 & \nu(\frac{4+\sigma_s}{4}U_1+6U_2) \end{pmatrix}, \tag{246}$$

$$h^{+1,m,s,\nu=\pm2}_6 = \begin{pmatrix} \nu W_1 & 0 & -i\sqrt{2m+2}\frac{v_*}{\ell} & 0 & \gamma\Sigma_m & i\sqrt{2m}\frac{v'_*}{\ell}\Sigma_{m-1} \\ 0 & \nu W1 & 0 & i\sqrt{2m-2}\frac{v_*}{\ell} & -i\sqrt{2m}\frac{v'_*}{\ell}\Sigma_m & \gamma\Sigma_{m-1} \\ +i\sqrt{2m+2}\frac{v_*}{\ell} & 0 & \nu(W_3+\sigma_s\frac{J}{4}) & 0 & 0 & 0 \\ 0 & -i\sqrt{2m-2}\frac{v_*}{\ell} & 0 & \nu(W_3+\sigma_s\frac{J}{4}) & 0 & 0 \\ \gamma\Sigma_m & i\sqrt{2m}\frac{v'_*}{\ell}\Sigma_m & 0 & 0 & \nu(\frac{4+\sigma_s}{4}U_1+6U_2) & 0 \\ -i\sqrt{2m}\frac{v'_*}{\ell}\Sigma_{m-1} & \gamma\Sigma_{m-1} & 0 & 0 & 0 & \nu(\frac{4+\sigma_s}{4}U_1+6U_2) \end{pmatrix} \tag{247}$$

The magnetic subbands within the narrow bands from the coupled modes emanate out of the $\mathbf{B}\to0$ energy eigenvalue of the above decoupled matrices, $\nu(W_3+\sigma_s\frac{J}{4})$, which is 2 fold degenerate for matrix in Eq.(247)$\forall m$ and singly degenerate for matrices in Eq.(245) and Eq.(246), for each spin projection. Including the decoupled c mode, we have $2m_{max}+3$ magnetic modes emanating out of this $\mathbf{B}\to0$ energy eigenvalue. Now recall that we have $2q-(2m_{max}+3)$ decoupled f modes with energy $\nu(\frac{4+\sigma_s}{4}U_1+6U_2)$. Thus in total we have $2q$ magnetic modes within the narrow bands, which corresponds to 2 states per moiré unit cell for each spin projection. The spectrum for flat band limit has been shown in Supplementary Fig.(6). Note that the $\mathbf{B}\to0$ energies recovered by the decoupled matrices are the corresponding zero field energies of THFM at Γ in mBZ.

Supplementary note 7. DISCUSSION FOR VALLEY $\mathbf{K}'$

In this section we discuss finite $\mathbf{B}$ THFM at valley $\mathbf{K}'$, i.e $\tau=-1$. Note that summation convention on repeated indices is implied unless explicitly stated. Using the SVD decomposition discussed we can re-write the matrix elements as

$$h^{-1}_{[1m0],[1r']}c^\dagger_{1-1k0ms}f_{1-1kr's} = \gamma\Sigma_{m',\bar{r}}c^\dagger_{1-1k0ms}U_{mm'}V_{\bar{r},r'}f_{1-1kr's} = \sum_{m=0}^{m_{1,-1}}\sum_{\bar{r}=0}^{m_{1,-1}}\gamma\Sigma_m\delta_{m\bar{r}}c^\dagger_{1-1k0ms}\bar{f}_{1-1k\bar{r}s}, \tag{248}$$

where we used the fact that U is an identity matrix and the rectangular matrix $\Sigma_{m,r'}$ is non-zero only along its main diagonal. Similarly

$$h^{-1}_{[2m0],[1r']}c^{\dagger}_{2-1k0ms}f_{1-1kr's} = -i\sqrt{2}\frac{v'_*}{\ell}\sum_{m=0}^{m_{2,-1}}\sqrt{m}c^{\dagger}_{2-1k0ms}\Sigma_{m-1,\bar{r}}V_{\bar{r},r'}f_{1-1kr's}$$
$$= -i\sqrt{2}\frac{v'_*}{\ell}\sum_{m=0}^{m_{2,-1}}\sum_{\bar{r}=0}^{m_{2,-1}-1}\sqrt{m}\Sigma_{\bar{r}}\delta_{m-1,\bar{r}}c^{\dagger}_{2-1k0ms}\bar{f}_{1-1k\bar{r}s}. \quad (249)$$

Because $m_{2,-1}-1 = m_{1,-1} = m_{max}$, the upper bound on the $\bar{r}$ summation is the same in Eq.(248) and Eq.(249). Similarly

$$h^{-1}_{[1m0],[2r']}c^{\dagger}_{1-1k0ms}f_{2-1kr's} = i\sqrt{2}\frac{v'_*}{\ell}\sum_{m=0}^{m_{1,-1}}\sqrt{m+1}c^{\dagger}_{1-1k0ms}\Sigma_{m+1,\bar{r}}V_{\bar{r},r'}f_{2-1kr's}$$
$$= i\sqrt{2}\frac{v'_*}{\ell}\sum_{m=0}^{m_{1,-1}}\sum_{\bar{r}=0}^{m_{1,-1}+1}\sqrt{m+1}c^{\dagger}_{1-1k0ms}\bar{f}_{2-1k\bar{r}s} \quad (250)$$

$$h^{1}_{[2m0],[2r']}c^{\dagger}_{2-1k0ms}f_{2-1kr's} = \sum_{m=0}^{m_{2,-1}}\sum_{\bar{r}=0}^{m_{2,-1}}\gamma\Sigma_m\delta_{m,\bar{r}}c^{\dagger}_{2-1k0ms}\bar{f}_{2-1k\bar{r}s}. \quad (251)$$

Because $m_{1,-1}+1 = m_{2,-1} = m_{max}+1$, the upper bound on $\bar{r}$ summation is the same in Eq.(250) and Eq.(251). Thus for a given k, out of the available $2q$ $f-$modes, only $2m_{max}+3$ couple.

A. CNP

The interactions in $\bar{f}$ basis at CNP [2] can be given as

$$V^{f,\tau=-1,s} = \sum_{k\in[0,1)\otimes[0,\frac{1}{q})}V^{f,\tau=-1,s}_{coupled} + V^{f,\tau=-1,s}_{decoupled}, \quad (252)$$

$$V^{f,\tau=-1,s}_{coupled} = \frac{U_1}{2}\left(\sum_{m=0}^{m_{max}}\bar{f}^{\dagger}_{1-1kms}\bar{f}_{1-1kms} + \sum_{m=0}^{m_{max}+1}\bar{f}^{\dagger}_{2-1kms}\bar{f}_{2-1kms}\right), \quad (253)$$

$$V^{f,\tau=-1,s}_{decoupled} = \frac{U_1}{2}\sum_{b=1}^{2}\sum_{m'=m_{max}+b}^{q-1}\bar{f}^{\dagger}_{b-1km's}\bar{f}_{b-1km's}, \quad (254)$$

Note that there are $2q-(2m_{max}+3)$ decoupled f modes for each k. Physically this corresponds to $2-(2m_{max}+3)/q$ states per moiré unit cell. The coupled modes can then be described as

$$H^{\tau=-1,s}_{coupled} = \sum_k\sum_{\alpha,\alpha'=1}^{6}\sum_{m=0}^{m_\alpha}\sum_{m'=0}^{m_{\alpha'}}\Xi_{m\alpha,m'\alpha'}\bar{d}^{\dagger}_{m\alpha s}(k)\bar{d}_{m'\alpha's}(k), \quad (255)$$

where $m_{\alpha=1,\dots,4} = m_{\alpha,-1}$, $m_5 = m_{max}$ and $m_6 = m_{max}+1$, and

$$\bar{d}^{\dagger}_{m\alpha s}(k) = \left(c^{\dagger}_{1-1k0ms}, c^{\dagger}_{2-1k0ms}, c^{\dagger}_{3-1k0ms}, c^{\dagger}_{4-1k0ms}, \bar{f}^{\dagger}_{1-1kms}, \bar{f}^{\dagger}_{2-1kms}\right)_\alpha. \quad (256)$$

The following operator can be defined for $\tau=-1$

$$\hat{h}^{-1}_{\alpha,\alpha'} = \begin{pmatrix} 0 & 0 & -i\sqrt{2}\frac{v_*}{\ell}\hat{a}^\dagger & 0 & \gamma\Sigma(\hat{a}^\dagger\hat{a}) & i\sqrt{2}\frac{v'_*}{\ell}\hat{a}\Sigma(\hat{a}^\dagger\hat{a}) \\ 0 & 0 & 0 & i\sqrt{2}\frac{v_*}{\ell}\hat{a} & -i\sqrt{2}\frac{v'_*}{\ell}\hat{a}^\dagger\Sigma(\hat{a}^\dagger\hat{a}) & \gamma\Sigma(\hat{a}^\dagger\hat{a}) \\ i\sqrt{2}\frac{v_*}{\ell}\hat{a} & 0 & \frac{J}{2} & M & 0 & 0 \\ 0 & -i\sqrt{2}\frac{v_*}{\ell}\hat{a}^\dagger & M & \frac{J}{2} & 0 & 0 \\ \gamma\Sigma(\hat{a}^\dagger\hat{a}) & i\sqrt{2}\frac{v'_*}{\ell}\Sigma(\hat{a}^\dagger\hat{a})\hat{a} & 0 & 0 & \frac{U_1}{2} & 0 \\ -i\sqrt{2}\frac{v'_*}{\ell}\Sigma(\hat{a}^\dagger\hat{a})\hat{a}^\dagger & \gamma\Sigma(\hat{a}^\dagger\hat{a}) & 0 & 0 & 0 & \frac{U_1}{2} \end{pmatrix}_{\alpha,\alpha'}, \quad (257)$$

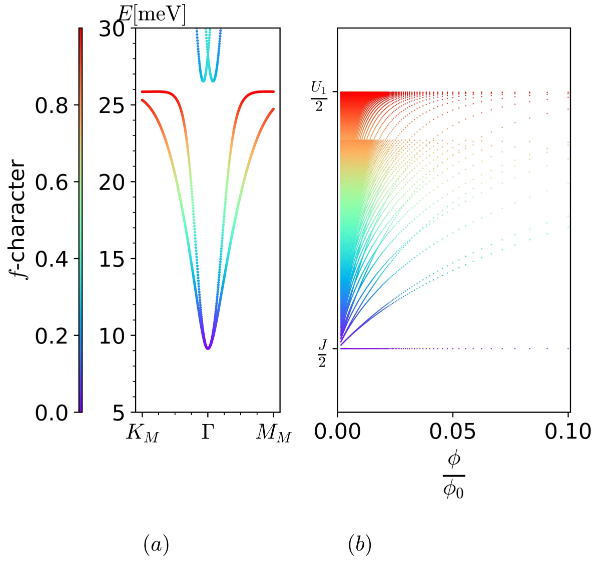

Supplementary Figure 7. Interacting heavy fermion Hofstadter spectra for (b) CNP at $\mathbf{K}'$ contrasted with (a) corresponding zero-field spectra at $w_0/w_1 = 0.7$ in the flat band limit $M = 0$. $m_{max} = \lceil \frac{q-3}{2} \rceil$.

where $\hat{a}$ is a simple h.o. lowering operator in terms of which matrix $\bar{\Xi}_{m\alpha,m'\alpha'}$ can be expressed as

$$\bar{\Xi}_{m\alpha,m'\alpha'} = \langle m|\hat{h}^{-1}_{\alpha,\alpha'}|m'\rangle. \tag{258}$$

Here $|m\rangle$ is a simple h.o. eigenstate and $\Sigma(m) = \Sigma_m$. We now discuss the the exact solutions to the eigenstates of the operator in Eq.(257) in flat band limit, $M = 0$. The $\mathbf{B}$ field independent $J/2$ Landau level comes from the anomalous c-mode

$$\theta_1 = [0, 0, 0, |0\rangle, 0, 0]^T. \tag{259}$$

The rest of the problem can be solved using the following ansätze:

$$\bar{\theta}_3 = \left[0, c^{(3)}_2 |0\rangle, 0, c^{(3)}_4 |1\rangle, 0, c^{(3)}_6 |0\rangle\right]^T, \tag{260}$$

$$\bar{\theta}_5 = \left[c^{(5)}_1 |0\rangle, c^{(5)}_2 |1\rangle, 0, c^{(5)}_4 |2\rangle, c^{(5)}_5 |0\rangle, c^{(5)}_6 |1\rangle\right]^T, \tag{261}$$

$$\bar{\theta}_{6_m} = \left[c^{(6_m)}_1 |m-1\rangle, c^{(6_m)}_2 |m\rangle, c^{(6_m)}_3 |m-2\rangle, c^{(6_m)}_4 |m+1\rangle, c^{(6_m)}_5 |m-1\rangle, c^{(6_m)}_6 |m\rangle\right]^T, \tag{262}$$

where $m \in \{2, \ldots, m_{max}+1\}$. $c^{(\beta)}_\alpha$ denotes the coefficient of corresponding h.o state at index α in the 6-component spinor in Eq.(256) and β labels the ansatz index θ_β. Using the above, we can set up the eigen-equation and solve for the corresponding coefficients.

The ansätze $\bar{\theta}_3$ and $\bar{\theta}_5$ yield the 3×3 and 5×5 Hermitian matrices, whose eigenvectors are $c^{(3)}_\alpha$ and $c^{(5)}_\alpha$, respectively:

$$h^{-1}_3 = \begin{pmatrix} 0 & i\frac{\sqrt{2}v_*}{\ell} & \gamma\Sigma_0 \\ -i\frac{\sqrt{2}v_*}{\ell} & \frac{J}{2} & 0 \\ \gamma\Sigma_0 & 0 & \frac{U_1}{2} \end{pmatrix}, \tag{263}$$

$$h^{-1}_5 = \begin{pmatrix} 0 & 0 & 0 & \gamma\Sigma_0 & i\frac{\sqrt{2}v'_*}{\ell}\Sigma_1 \\ 0 & 0 & i\frac{2v_*}{\ell} & -i\frac{\sqrt{2}v'_*}{\ell}\Sigma_0 & \gamma\Sigma_1 \\ 0 & -i\frac{2v_*}{\ell} & \frac{J}{2} & 0 & 0 \\ \gamma\Sigma_0 & i\frac{\sqrt{2}v'_*}{\ell}\Sigma_0 & 0 & \frac{U_1}{2} & 0 \\ -i\frac{\sqrt{2}v'_*}{\ell}\Sigma_1 & \gamma\Sigma_1 & 0 & 0 & \frac{U_1}{2} \end{pmatrix}. \tag{264}$$

Similarly, the ansatz $\bar{\theta}_6^m$ yields the following 6×6 Hermitian matrix for each m, whose eigenvectors are $c_\alpha^{(6,m)}$:

$$h_6^{-1,m} = \begin{pmatrix} 0 & 0 & -i\sqrt{2m-2}\frac{v_*}{\ell} & 0 & \gamma\Sigma_{m-1} & i\sqrt{2m}\frac{v_*'}{\ell}\Sigma_m \\ 0 & 0 & 0 & i\sqrt{2m+2}\frac{v_*}{\ell} & -i\sqrt{2m}\frac{v_*'}{\ell}\Sigma_{m-1} & \gamma\Sigma_m \\ +i\sqrt{2m-2}\frac{v_*}{\ell} & 0 & \frac{J}{2} & 0 & 0 & 0 \\ 0 & -i\sqrt{2m+2}\frac{v_*}{\ell} & 0 & \frac{J}{2} & 0 & 0 \\ \gamma\Sigma_{m-1} & i\sqrt{2m}\frac{v_*'}{\ell}\Sigma_{m-1} & 0 & 0 & \frac{U_1}{2} & 0 \\ -i\sqrt{2m}\frac{v_*'}{\ell}\Sigma_m & \gamma\Sigma_m & 0 & 0 & 0 & \frac{U_1}{2} \end{pmatrix}. \quad (265)$$

The magnetic subbands within the narrow bands from the coupled modes emanate out of the $\mathbf{B}\to 0$ energy eigenvalue of the above decoupled matrices, $\frac{J}{2}$, which is 2 fold degenerate for matrix in Eq.(265)$\forall m$ and singly degenerate for matrices in Eq.(263) and Eq.(264). Including the decoupled c mode, we have $2m_{max}+3$ magnetic modes emanating out of this $\mathbf{B}\to 0$ energy eigenvalue. Now recall that we have $2q-(2m_{max}+3)$ decoupled f modes with energy $\frac{U_1}{2}$. Thus in total we have $2q$ magnetic modes within the narrow bands, which corresponds to 2 states per moiré unit cell per spin. The spectrum for flat band limit has been shown in Supplementary Fig.(7b). Note that the $\mathbf{B}\to 0$ energies recovered by the decoupled matrices are the corresponding zero field energies of THFM at Γ in mBZ.

B. $\nu=\pm 1$ Spin $\uparrow\downarrow$

The interactions at $\nu=\pm1$ for valley $\mathbf{K}'$ spin $\uparrow\downarrow$[2] is given as

$$V_{\nu=\pm1}^{\tau=-1,s=\uparrow\downarrow} = \nu\sum_k\Bigg(\sum_{a=1}^{4}\sum_{m=0}^{m_{a,-1}}\sum_{r=0}^{p-1} W_a c^\dagger_{a-1krms}c_{a-1krms} - \sum_{a=3,4}\sum_{m=0}^{m_{a,-1}}\sum_{r=0}^{p-1}\frac{J}{2}c^\dagger_{a-1krms}c_{a-1krms} \\ + \sum_{b=1,2}\sum_{r'=0}^{q-1}\left(\frac{1}{2}U_1+6U_2\right)f^\dagger_{b-1kr's}f_{b-1kr's}\Bigg), \quad (266)$$

$W_{a\in\{1\ldots4\}}$ and U_2 are mean field coefficients with $W_1=W_2$ and $W_3=W_4$ [2]. The interaction for f modes in the $\bar{f}$ basis can then be given as

$$V_{\nu=\pm1}^{f,\tau=-1,s} = \sum_{k\in[0,1)\otimes[0,\frac{1}{q})} V_{coupled}^{f,\tau=-1,s,\nu=\pm1} + V_{decoupled}^{f,\tau=-1,s,\nu=\pm1} \quad (267)$$

where

$$V_{coupled}^{f,\tau=-1,s,\nu=\pm1} = \nu\left(\frac{1}{2}U_1+6U_2\right)\left(\sum_{m=0}^{m_{max}}\bar{f}^\dagger_{1-1kms}\bar{f}_{1-1kms}+\sum_{m=0}^{m_{max}+1}\bar{f}^\dagger_{2-1kms}\bar{f}_{2-1kms}\right), \quad (268)$$

$$V_{decoupled}^{f,\tau=-1,s,\nu=\pm1} = \nu\sum_{b=1}^{2}\sum_{m'=m_{max}+b}^{q-1}\left(\frac{1}{2}U_1+6U_2\right)\bar{f}^\dagger_{b-1km's}\bar{f}_{b-1km's}. \quad (269)$$

Yet again there are $2q-(2m_{max}+3)$ decoupled f modes for each k. Physically this corresponds to $2-(2m_{max}+3)/q$ states per moiré unit cell for each spin projection. The coupled modes can then be described by

$$H_{coupled}^{\tau=-1,s,\nu=\pm1} = \sum_k\sum_{\alpha,\alpha'=1}^{6}\sum_{m=0}^{m_\alpha}\sum_{m'=0}^{m_{\alpha'}}\Xi^{s,\nu=\pm2}_{m\alpha,m'\alpha'}\bar{d}^\dagger_{m\alpha s}(k)\bar{d}_{m'\alpha' s}(k), \quad (270)$$

where $m_{\alpha=1,\ldots,4}=m_{\alpha,-1}$, $m_5=m_{max}$ and $m_6=m_{max}+1$, and

$$\bar{d}^\dagger_{m\alpha s}(k) = \left(c^\dagger_{1-1k0ms}, c^\dagger_{2-1k0ms}, c^\dagger_{3-1k0ms}, c^\dagger_{4-1k0ms}, \bar{f}^\dagger_{1-1kms}, \bar{f}^\dagger_{2-1kms}\right)_\alpha. \quad (271)$$

with

$$\Xi^{s,\nu=\pm1}_{m\alpha,m'\alpha'} = \langle m|\hat{h}^{-1,s,\nu=\pm1}_{\alpha,\alpha'}|m'\rangle. \quad (272)$$

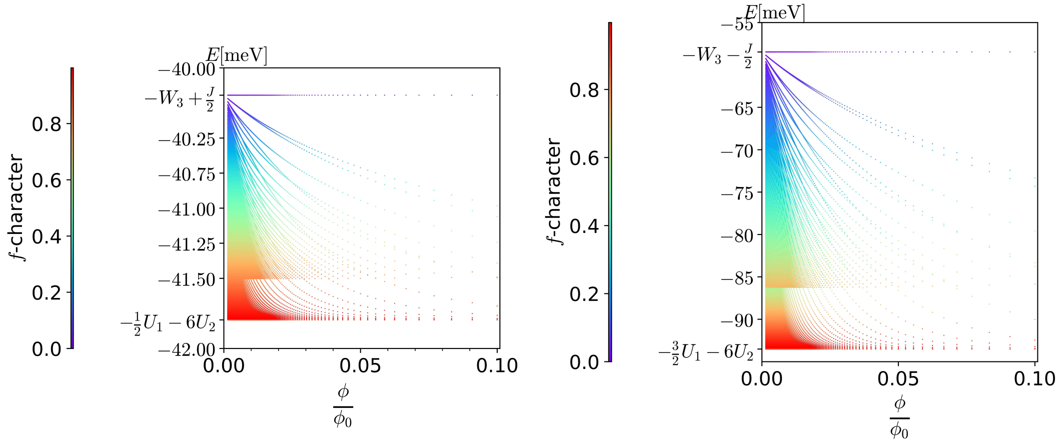

(a) (b)

Supplementary Figure 8. Interacting heavy fermion Hofstadter spectra for (a) valley $\mathbf{K}'$ spin $\uparrow\downarrow$ (degenerate) and (b) valley $\mathbf{K}$ spin $\uparrow$ at filling $\nu = -1$ at $w_0/w_1 = 0.7$ in the flat band limit $M = 0$. The value of parameters used are $W_1 = 44.05$meV, $W_3 = 49.33$meV and $U_2 = 2.656$meV. $m_{max} = \lceil \frac{q-3}{2} \rceil$.

where the operators $\hat{h}^{-1,s,\nu=\pm1}_{\alpha,\alpha'}$ are given as

$$\hat{h}^{-1,s,\nu=\pm1}_{\alpha,\alpha'} = \begin{pmatrix} \nu W_1 & 0 & -i\sqrt{2}\frac{v_*}{\ell}\hat{a}^\dagger & 0 & \gamma\Sigma(\hat{a}^\dagger\hat{a}) & i\sqrt{2}\frac{v'_*}{\ell}\hat{a}\Sigma(\hat{a}^\dagger\hat{a}) \\ 0 & \nu W_1 & 0 & i\sqrt{2}\frac{v_*}{\ell}\hat{a} & -i\sqrt{2}\frac{v'_*}{\ell}\hat{a}^\dagger\Sigma(\hat{a}^\dagger\hat{a}) & \gamma\Sigma(\hat{a}^\dagger\hat{a}) \\ i\sqrt{2}\frac{v_*}{\ell}\hat{a} & 0 & \nu(W_3 - \frac{J}{2}) & M & 0 & 0 \\ 0 & -i\sqrt{2}\frac{v_*}{\ell}\hat{a}^\dagger & M & \nu(W_3 - \frac{J}{2}) & 0 & 0 \\ \gamma\Sigma(\hat{a}^\dagger\hat{a}) & i\sqrt{2}\frac{v'_*}{\ell}\Sigma(\hat{a}^\dagger\hat{a})\hat{a} & 0 & 0 & \nu(\frac{U_1}{2} + 6U_2) & 0 \\ -i\sqrt{2}\frac{v'_*}{\ell}\Sigma(\hat{a}^\dagger\hat{a})\hat{a}^\dagger & \gamma\Sigma(\hat{a}^\dagger\hat{a}) & 0 & 0 & 0 & \nu(\frac{U_1}{2} + 6U_2) \end{pmatrix}_{\alpha,\alpha'} \tag{273}$$

where $\hat{a}$ is a simple h.o. lowering operator with $|m\rangle$ being a simple h.o. eigenstate and $\Sigma(m) = \Sigma_m$. The exact eigenstates for the above operator are exactly solvable in flat band limit, $M = 0$. The field independent $\nu(W_3 - \frac{J}{2})$ level is formed by the decoupled anomalous c level given in Eq.(259). The rest of eigenstates can be solved using the ansätze given in Eq.(260)-Eq.(262). The corresponding coefficients $c^{(3)}_\alpha$, $c^{(5)}_\alpha$ and $c^{(6,m)}_\alpha$ can be solved as eigenvectors of the following 3×3, 5×5 and m_{max} 6×6 Hermitian matrices respectively:

$$h_3^{-1,\nu=\pm1} = \begin{pmatrix} \nu W_1 & i\frac{\sqrt{2}v_*}{\ell} & \gamma\Sigma_0 \\ -i\frac{\sqrt{2}v_*}{\ell} & \nu(W_3 - \frac{J}{2}) & 0 \\ \gamma\Sigma_0 & 0 & \nu(\frac{U_1}{2} + 6U_2) \end{pmatrix}, \tag{274}$$

$$h_5^{-1,\nu=\pm1} = \begin{pmatrix} \nu W_1 & 0 & 0 & \gamma\Sigma_0 & i\frac{\sqrt{2}v'_*}{\ell}\Sigma_1 \\ 0 & \nu W_1 & i\frac{2v_*}{\ell} & -i\frac{\sqrt{2}v'_*}{\ell}\Sigma_0 & \gamma\Sigma_1 \\ 0 & -i\frac{2v_*}{\ell} & \nu(W_3 - \frac{J}{2}) & 0 & 0 \\ \gamma\Sigma_0 & i\frac{\sqrt{2}v'_*}{\ell}\Sigma_0 & 0 & \nu(\frac{U_1}{2} + 6U_2) & 0 \\ -i\frac{\sqrt{2}v'_*}{\ell}\Sigma_1 & \gamma\Sigma_1 & 0 & 0 & \nu(\frac{U_1}{2} + 6U_2) \end{pmatrix}. \tag{275}$$

$$h_6^{-1,m,\nu=\pm1} = \begin{pmatrix} \nu W_1 & 0 & -i\sqrt{2m-2}\frac{v_*}{\ell} & 0 & \gamma\Sigma_{m-1} & i\sqrt{2m}\frac{v'_*}{\ell}\Sigma_m \\ 0 & \nu W_1 & 0 & i\sqrt{2m+2}\frac{v_*}{\ell} & -i\sqrt{2m}\frac{v'_*}{\ell}\Sigma_{m-1} & \gamma\Sigma_m \\ +i\sqrt{2m-2}\frac{v_*}{\ell} & 0 & \nu(W_3 - \frac{J}{2}) & 0 & 0 & 0 \\ 0 & -i\sqrt{2m+2}\frac{v_*}{\ell} & 0 & \nu(W_3 - \frac{J}{2}) & 0 & 0 \\ \gamma\Sigma_{m-1} & i\sqrt{2m}\frac{v'_*}{\ell}\Sigma_{m-1} & 0 & 0 & \nu(\frac{U_1}{2} + 6U_2) & 0 \\ -i\sqrt{2m}\frac{v'_*}{\ell}\Sigma_m & \gamma\Sigma_m & 0 & 0 & 0 & \nu(\frac{U_1}{2} + 6U_2) \end{pmatrix} \tag{276}$$

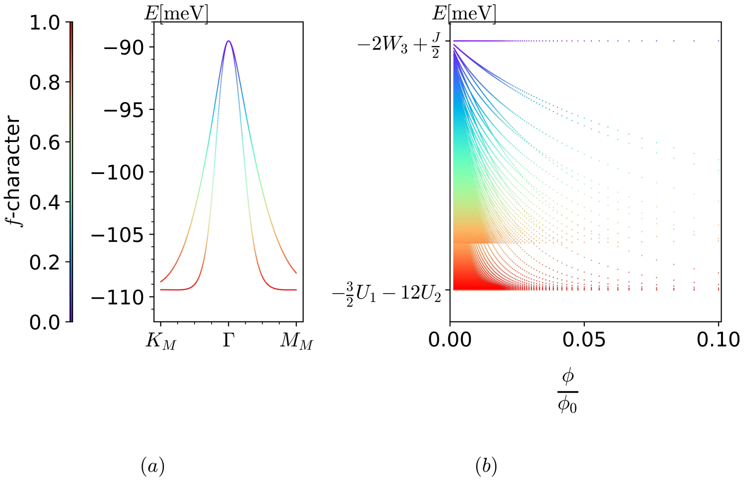

Supplementary Figure 9. Interacting heavy fermion Hofstadter spectra for (b) valley $\mathbf{K}'$ spin ↑↓ (degenerate) contrasted with (a) corresponding zero-field spectra at filling $\nu=-2$ at $w_0/w_1=0.7$ in the flat band limit $M=0$. $m_{max}=\lceil\frac{q-3}{2}\rceil$.

The magnetic subbands within the narrow bands from the coupled modes emanate out of the $\mathbf{B}\to 0$ energy eigenvalue of the above decoupled matrices, $\nu(W_3-\frac{J}{2})$, which is 2 fold degenerate for matrix in Eq.(276)$\forall m$ and singly degenerate for matrices in Eq.(274) and Eq.(275). Including the decoupled c mode, we have $2m_{max}+3$ magnetic modes emanating out of this $\mathbf{B}\to 0$ energy eigenvalue. Now recall that we have $2q-(2m_{max}+3)$ decoupled f modes with energy $\nu(\frac{U_1}{2}+6U_2)$. Thus in total we have $2q$ magnetic modes within the narrow bands, which corresponds to 2 states per moiré unit cell for each spin projection. The spectrum for flat band limit has been shown in Supplementary Fig.(8a). Note that the $\mathbf{B}\to 0$ energies recovered by the decoupled matrices are the corresponding zero field energies of THFM at Γ in mBZ.

C. $\nu=\pm 2$ Spin ↑↓

The interactions at $\nu=\pm 2$ for valley $\mathbf{K}'$ spin ↑↓[2] is given as

$$V^{\tau=-1,s=\uparrow\downarrow}_{\nu=\pm 2}=\nu\sum_k\Bigg(\sum_{a=1}^{4}\sum_{m=0}^{m_{a,-1}}\sum_{r=0}^{p-1}W_a c^\dagger_{a-1krms}c_{a-1krms}-\sum_{a=3,4}\sum_{m=0}^{m_{a,-1}}\sum_{r=0}^{p-1}\frac{J}{4}c^\dagger_{a-1krms}c_{a-1krms}$$
$$+\sum_{b=1,2}\sum_{r'=0}^{q-1}\left(\frac{3}{4}U_1+6U_2\right)f^\dagger_{b-1kr's}f_{b-1kr's}\Bigg). \tag{277}$$

The interaction for f modes in the $\bar{f}$ basis can then be given as

$$V^{f,\tau=-1,s}_{\nu=\pm 1}=\sum_{k\in[0,1)\otimes[0,\frac{1}{q})}V^{f,\tau=-1,s,\nu=\pm 2}_{coupled}+V^{f,\tau=-1,s,\nu=\pm 2}_{decoupled} \tag{278}$$

where

$$V^{f,\tau=-1,s,\nu=\pm 2}_{coupled}=\nu\left(\frac{3}{4}U_1+6U_2\right)\left(\sum_{m=0}^{m_{max}}\bar{f}^\dagger_{1-1kms}\bar{f}_{1-1kms}+\sum_{m=0}^{m_{max}+1}\bar{f}^\dagger_{2-1kms}\bar{f}_{2-1kms}\right), \tag{279}$$

$$V^{f,\tau=-1,s,\nu=\pm 2}_{decoupled}=\nu\sum_{b=1}^{2}\sum_{m'=m_{max}+b}^{q-1}\left(\frac{3}{4}U_1+6U_2\right)\bar{f}^\dagger_{b-1km's}\bar{f}_{b-1km's}. \tag{280}$$

Yet again there are $2q-(2m_{max}+3)$ decoupled f modes for each k. Physically this corresponds to $2-(2m_{max}+3)/q$ states per moiré unit cell for each spin projection. The coupled modes can then be described by

$$H^{\tau=-1,s,\nu=\pm 2}_{coupled}=\sum_k\sum_{\alpha,\alpha'=1}^{6}\sum_{m=0}^{m_\alpha}\sum_{m'=0}^{m_{\alpha'}}\Xi^{s,\nu=\pm 1}_{m\alpha,m'\alpha'}\bar{d}^\dagger_{m\alpha s}(k)\bar{d}_{m'\alpha' s}(k), \tag{281}$$

where $m_{\alpha=1,\ldots,4} = m_{\alpha,-1}$, $m_5 = m_{max}$ and $m_6 = m_{max}+1$, and

$$\bar{d}^{\dagger}_{m\alpha s}(k) = \left(c^{\dagger}_{1-1k0ms}, c^{\dagger}_{2-1k0ms}, c^{\dagger}_{3-1k0ms}, c^{\dagger}_{4-1k0ms}, \bar{f}^{\dagger}_{1-1kms}, \bar{f}^{\dagger}_{2-1kms}\right)_{\alpha}. \tag{282}$$

with

$$\Xi^{s,\nu=\pm 2}_{m\alpha,m'\alpha'} = \langle m|\hat{h}^{-1,s,\nu=\pm 2}_{\alpha,\alpha'}|m'\rangle, \tag{283}$$

where the operators $\hat{h}^{-1,s,\nu=\pm 2}_{\alpha,\alpha'}$ are given as

$$\hat{h}^{-1,s,\nu=\pm 2}_{\alpha,\alpha'} = \begin{pmatrix} \nu W_1 & 0 & -i\sqrt{2}\frac{v_*}{\ell}\hat{a}^{\dagger} & 0 & \gamma\Sigma(\hat{a}^{\dagger}\hat{a}) & i\sqrt{2}\frac{v'_*}{\ell}\hat{a}\Sigma(\hat{a}^{\dagger}\hat{a}) \\ 0 & \nu W_1 & 0 & i\sqrt{2}\frac{v_*}{\ell}\hat{a} & -i\sqrt{2}\frac{v'_*}{\ell}\hat{a}^{\dagger}\Sigma(\hat{a}^{\dagger}\hat{a}) & \gamma\Sigma(\hat{a}^{\dagger}\hat{a}) \\ i\sqrt{2}\frac{v_*}{\ell}\hat{a} & 0 & \nu(W_3-\frac{J}{4}) & M & 0 & 0 \\ 0 & -i\sqrt{2}\frac{v_*}{\ell}\hat{a}^{\dagger} & M & \nu(W_3-\frac{J}{4}) & 0 & 0 \\ \gamma\Sigma(\hat{a}^{\dagger}\hat{a}) & i\sqrt{2}\frac{v'_*}{\ell}\Sigma(\hat{a}^{\dagger}\hat{a})\hat{a} & 0 & 0 & \nu(\frac{3U_1}{4}+6U_2) & 0 \\ -i\sqrt{2}\frac{v'_*}{\ell}\Sigma(\hat{a}^{\dagger}\hat{a})\hat{a}^{\dagger} & \gamma\Sigma(\hat{a}^{\dagger}\hat{a}) & 0 & 0 & 0 & \nu(\frac{3U_1}{4}+6U_2) \end{pmatrix}_{\alpha,\alpha'} \tag{284}$$

where $\hat{a}$ is a simple h.o. lowering operator with $|m\rangle$ being a simple h.o. eigenstate and $\Sigma(m) = \Sigma_m$. The exact eigenstates for the above operator are exactly solvable in flat band limit, $M = 0$. The field independent $\nu(W_3 - \frac{J}{4})$ level is formed by the decoupled anomalous c level given in Eq.(259). The rest of eigenstates can be solved using the ansätze given in Eq.(260)-Eq.(262). The corresponding coefficients $c^{(3)}_{\alpha}$, $c^{(5)}_{\alpha}$ and $c^{(6,m)}_{\alpha}$ can be solved as eigenvectors of the following 3×3, 5×5 and m_{max} 6×6 Hermitian matrices respectively:

$$h_3^{-1,\nu=\pm 2} = \begin{pmatrix} \nu W_1 & i\frac{\sqrt{2}v_*}{\ell} & \gamma\Sigma_0 \\ -i\frac{\sqrt{2}v_*}{\ell} & \nu(W_3-\frac{J}{4}) & 0 \\ \gamma\Sigma_0 & 0 & \nu(\frac{3U_1}{4}+6U_2) \end{pmatrix}, \tag{285}$$

$$h_5^{-1,\nu=\pm 2} = \begin{pmatrix} \nu W_1 & 0 & 0 & \gamma\Sigma_0 & i\frac{\sqrt{2}v'_*}{\ell}\Sigma_1 \\ 0 & \nu W_1 & i\frac{2v_*}{\ell} & -i\frac{\sqrt{2}v'_*}{\ell}\Sigma_0 & \gamma\Sigma_1 \\ 0 & -i\frac{2v_*}{\ell} & \nu(W_3-\frac{J}{4}) & 0 & 0 \\ \gamma\Sigma_0 & i\frac{\sqrt{2}v'_*}{\ell}\Sigma_0 & 0 & \nu(\frac{3U_1}{4}+6U_2) & 0 \\ -i\frac{\sqrt{2}v'_*}{\ell}\Sigma_1 & \gamma\Sigma_1 & 0 & 0 & \nu(\frac{3U_1}{4}+6U_2) \end{pmatrix}. \tag{286}$$

$$h_6^{-1,m,\nu=\pm 2} = \begin{pmatrix} \nu W_1 & 0 & -i\sqrt{2m-2}\frac{v_*}{\ell} & 0 & \gamma\Sigma_{m-1} & i\sqrt{2m}\frac{v'_*}{\ell}\Sigma_m \\ 0 & \nu W_1 & 0 & i\sqrt{2m+2}\frac{v_*}{\ell} & -i\sqrt{2m}\frac{v'_*}{\ell}\Sigma_{m-1} & \gamma\Sigma_m \\ +i\sqrt{2m-2}\frac{v_*}{\ell} & 0 & \nu(W_3-\frac{J}{4}) & 0 & 0 & 0 \\ 0 & -i\sqrt{2m+2}\frac{v_*}{\ell} & 0 & \nu(W_3-\frac{J}{4}) & 0 & 0 \\ \gamma\Sigma_{m-1} & i\sqrt{2m}\frac{v'_*}{\ell}\Sigma_{m-1} & 0 & 0 & \nu(\frac{3U_1}{4}+6U_2) & 0 \\ -i\sqrt{2m}\frac{v'_*}{\ell}\Sigma_m & \gamma\Sigma_m & 0 & 0 & 0 & \nu(\frac{3U_1}{4}+6U_2) \end{pmatrix} \tag{287}$$

The magnetic subbands within the narrow bands from the coupled modes emanate out of the $\mathbf{B}\to 0$ energy eigenvalue of the above decoupled matrices, $\nu(W_3-\frac{J}{4})$, which is 2 fold degenerate for matrix in Eq.(287)$\forall m$ and singly degenerate for matrices in Eq.(285) and Eq.(286). Including the decoupled c mode, we have $2m_{max}+3$ magnetic modes emanating out of this $\mathbf{B}\to 0$ energy eigenvalue. Now recall that we have $2q-(2m_{max}+3)$ decoupled f modes with energy $\nu(\frac{3U_1}{4}+6U_2)$. Thus in total we have $2q$ magnetic modes within the narrow bands, which corresponds to 2 states per moiré unit cell for each spin projection. The spectrum for flat band limit has been shown in Supplementary Fig.(9b). Note that the $\mathbf{B}\to 0$ energies recovered by the decoupled matrices are the corresponding zero field energy of THFM at Γ in mBZ.

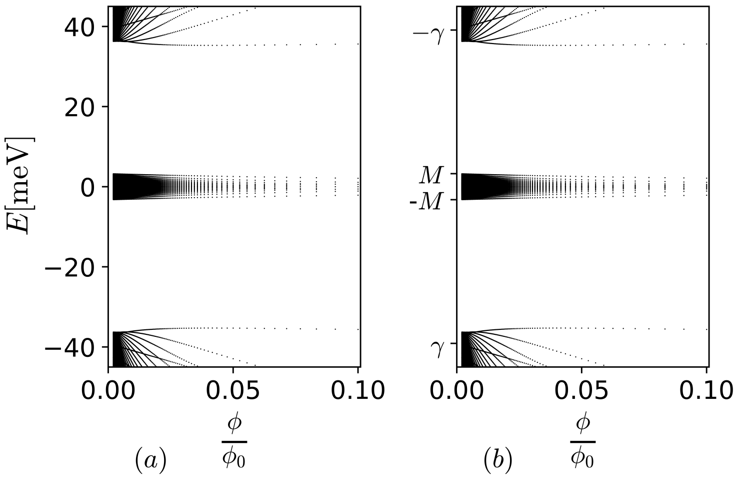

Supplementary Figure 10. The spin valley degenerate non-interacting Hofstadter spectrum for $M \neq 0$ at $\omega_0/\omega_1 = 0.7$ (a) obtained using naive minimal coupling compared to the (b) spectrum obtained using the formalism constructed in the paper for $\mathbf{B} \neq 0$ THFM. The magnetic subbands within the narrow bands are spread in the energy window of $|2M|$, which also sets the bandwidth of narrow bands at $\mathbf{B} = 0$ in THFM. The total number of subbands within the narrow bands i.e. in the energy widow [-M,M] at flux $1/q$ in (a) is $(2m_* + 1)$ while in (b) is correctly $2q$. We use $m_{max} = m_* = \lfloor \frac{q-3}{2} \rfloor$

.

Supplementary note 8. NAIVE MINIMAL SUBSTITUTION

Including both the c-c and c-f coupling in Eq.(2) of main-text, the zero $\mathbf{B}$ field THFM can be written as a 6×6 matrix, say at $\tau = +1$

$$h_0^{\tau=1} = \begin{pmatrix} 0 & 0 & v_* k & 0 & e^{-\frac{\mathbf{k}^2\lambda^2}{2}}\gamma & e^{-\frac{\mathbf{k}^2\lambda^2}{2}} v'_* \bar{k} \\ 0 & 0 & 0 & v_* \bar{k} & e^{-\frac{\mathbf{k}^2\lambda^2}{2}} v'_* k & e^{-\frac{\mathbf{k}^2\lambda^2}{2}}\gamma \\ v_* \bar{k} & 0 & 0 & M & 0 & 0 \\ 0 & v_* k & M & 0 & 0 & 0 \\ e^{-\frac{\mathbf{k}^2\lambda^2}{2}}\gamma & e^{-\frac{\mathbf{k}^2\lambda^2}{2}} v'_* \bar{k} & 0 & 0 & 0 & 0 \\ e^{-\frac{\mathbf{k}^2\lambda^2}{2}} v'_* k & e^{-\frac{\mathbf{k}^2\lambda^2}{2}}\gamma & 0 & 0 & 0 & 0 \end{pmatrix}_{\alpha,\alpha'} , \tag{288}$$

(289)

where $k = k_x + ik_y$ and $\bar{k} = k_x - ik_y$. Following a naive minimal substitution, we promote $k_x + ik_y \to -i\sqrt{2}\hat{a}/\ell$, where $\hat{a}$ is the Landau level (LL) lowering operator. Although it is completely unclear on how to perform the minimal substitution on $e^{-\frac{1}{2}\mathbf{k}^2\lambda^2}$, if we were to Taylor expand it as $1 - \frac{1}{2}\mathbf{k}^2\lambda^2 + \ldots = 1 - \frac{1}{2}\frac{k\bar{k}+\bar{k}k}{2}\lambda^2 + \ldots$ and use the finite $\mathbf{B}$ substitution for k and $\bar{k}$ mentioned above we get $1 - (a^\dagger a + \frac{1}{2})\frac{\lambda^2}{\ell^2} + \ldots$. If this operator acts on LL state $|m\rangle$, upto $\mathcal{O}(\frac{\lambda^2}{\ell^2})$, we get $1 - (m + \frac{1}{2})\frac{\lambda^2}{\ell^2}$. But recall that this is same as the $\mathbf{B} \to 0$ limit of Σ_m as given in Eq.(179). Based on this assumption, we thus promote $e^{-\frac{1}{2}\mathbf{k}^2\lambda^2}$ to $\Sigma_{a^\dagger a} = \Sigma(a^\dagger a)$ at finite $\mathbf{B}$. We thus have

$$\hat{h}_B^{\tau=1} = \begin{pmatrix} 0 & 0 & -i\sqrt{2}\frac{v_*}{\ell}\hat{a} & 0 & \gamma\Sigma(\hat{a}^\dagger\hat{a}) & i\sqrt{2}\frac{v'_*}{\ell}\hat{a}^\dagger\Sigma(\hat{a}^\dagger\hat{a}) \\ 0 & 0 & 0 & i\sqrt{2}\frac{v_*}{\ell}\hat{a}^\dagger & -i\sqrt{2}\frac{v'_*}{\ell}\hat{a}\Sigma(\hat{a}^\dagger\hat{a}) & \gamma\Sigma(\hat{a}^\dagger\hat{a}) \\ i\sqrt{2}\frac{v_*}{\ell}\hat{a}^\dagger & 0 & 0 & M & 0 & 0 \\ 0 & -i\sqrt{2}\frac{v_*}{\ell}\hat{a} & M & 0 & 0 & 0 \\ \gamma\Sigma(\hat{a}^\dagger\hat{a}) & i\sqrt{2}\frac{v'_*}{\ell}\Sigma(\hat{a}^\dagger\hat{a})\hat{a}^\dagger & 0 & 0 & 0 & 0 \\ -i\sqrt{2}\frac{v'_*}{\ell}\Sigma(\hat{a}^\dagger\hat{a})\hat{a} & \gamma\Sigma(\hat{a}^\dagger\hat{a}) & 0 & 0 & 0 & 0 \end{pmatrix}_{\alpha,\alpha'} , \tag{290}$$

where we assume that the correct way of ordering $\Sigma(a^\dagger a)$ with respect to the operators a and $a^\dagger$ (coming from the promotion of k and $\bar{k}$ respectively) is the one we have in Eq.(206). The Hofstadter spectrum based on the naive minimal substitution approach can thus be obtained by solving the eigenstates of $\hat{h}^{\tau=1}_{B_{\alpha,\alpha'}}$ in the LL basis. The LL basis $|m\rangle$ used for computing the matrix elements for index $\alpha \in \{1,6\}$ are: $m \in \{0, m_*\}$ for index $\alpha = 1$ ($a = 1$

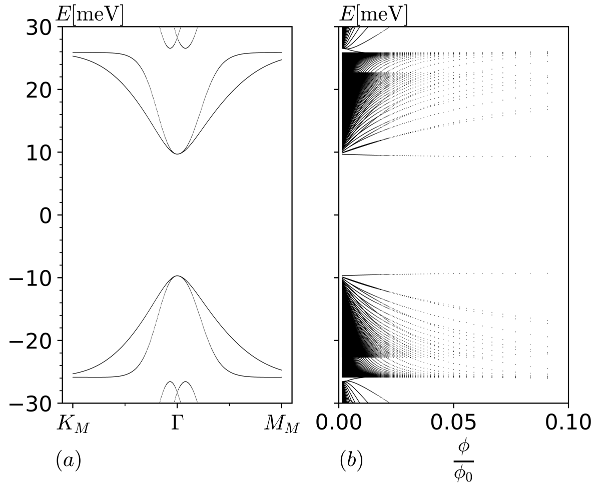

Supplementary Figure 11. Spin degenerate (b) interacting heavy fermion Hofstadter spectrum for KIVC state at CNP contrasted with (a) corresponding zero-field spectrum at $w_0/w_1 = 0.7$. $m_{max} = \lceil \frac{q-3}{2} \rceil$.

c-fermion), $m \in \{0, m_* - 1\}$ for index $\alpha = 2$ ($a = 2$ c-fermion), $m \in \{0, m_* + 1\}$ for index $\alpha = 3$ ($a = 1$ c-fermion), $m \in \{0, m_* - 2\}$ for index $\alpha = 4$ ($a = 1$ c-fermion), $m \in \{0, m_*\}$ for index $\alpha = 5$ ($b = 1$ f-fermion) and $m \in \{0, m_* - 1\}$ for index $\alpha = 6$ ($b = 2$ f-fermion). The spectrum is shown in Fig.(10a). Although the magnetic subbands within the narrow bands are well separated from the remote bands, the total number of states within the narrow bands is $(2m_\star + 1)\phi/\phi_0$ per moié unit cell per spin per valley (same as the number of zero modes for $M = 0$, shown in main-text), which of course is incorrect as the total number of states should rather be 2 per moiré unit cell per spin per valley, independent of $\mathbf{B}$.

Supplementary note 9. PARENT KRAMERS INTER-VALLEY COHERENT STATE

In this section, we discuss the Landau quantization of the one-shot Hartree Fock (HF) bands obtained for a parent Kramers inter-valley coherent (KIVC) state at fillings $\nu = 0$ (CNP) and $\nu = -2$.

A. CNP

The spin degenerate $\mathbf{B} = 0$ one-shot Hartree-Fock (HF) mean-field (MF) Hamiltonian for parent KIVC state at $\nu = 0$ is given as[2]

$$H^{\nu=0,s=\uparrow,\downarrow}_{\mathbf{0},\mathrm{KIVC}}(\mathbf{k}) = \begin{pmatrix} 0 & v_\star(k_x\sigma_0\tau_z + ik_y\sigma_z\tau_0) & e^{-\frac{\mathbf{k}^2\lambda^2}{2}}\left(\gamma\sigma_0\tau_0 + v'_\star(k_x\sigma_x\tau_z + k_y\sigma_y\tau_0)\right) \\ & -\frac{J}{2}\sigma_y\tau_y + M\sigma_x\tau_0 & 0 \\ h.c. & & \frac{U_1}{2}\sigma_y\tau_y \end{pmatrix}, \quad (291)$$

where $\mathbf{k} = (k_x, k_y)$ ∈moiré BZ, the Pauli matrices σ and τ act in the orbital and valley space respectively and h.c. represents hermitian conjugate.

Let us begin the discussion by first analyzing the decoupled $\bar{f}$ modes at $\mathbf{B} \neq 0$. Recall that at every k ∈magnetic BZ, $q - (m_{max} + 2)$ of $\bar{f}_{11kr's}$ and $\bar{f}_{2-1kr's}$ modes, i.e. the ones with $r' \geq m_{max} + 1$ decouple from the cs. Similarly $q - (m_{max} + 1)$ of $\bar{f}_{21kr's}$ and $\bar{f}_{1-1kr's}$ modes, i.e. the ones with $r' \geq m_{max}$ decouple from the c's at every k ∈magnetic BZ. We see that even for the KIVC state, the decoupled $\bar{f}$ modes contribute to $2q - (2m_{max} + 3)$ $\mathbf{B}$ independent energy levels at $\pm U_1/2$. Physically, these modes are the linear combination $\frac{1}{\sqrt{2}}\left(\bar{f}_{11kr's} \pm \bar{f}_{2-1kr's}\right)$ with $r' \geq m_{max} + 1$ and $\frac{1}{\sqrt{2}}\left(\bar{f}_{21kr's} \pm \bar{f}_{1-1kr's}\right)$ with $r' \geq m_{max}$, which contribute $q - (m_{max} + 2)$ and $q - (m_{max} + 1)$ modes at $\pm U_1/2$, respectively. We thus have $2q - (2m_{max} + 3)$ modes at $\pm U_1/2$ as motivated above.

Before discussing the Hofstadter spectrum for the coupled modes, we first perform a $U(4)$ rotation ($e^{i\frac{\pi}{2}\hat{\Sigma}_{x0}}$, where $\hat{\Sigma}_{x0}$ is defined in Eq.(4) of Ref.[2])) on the zero magnetic field Hamiltonian in Eq.(291) to re-write it in a much

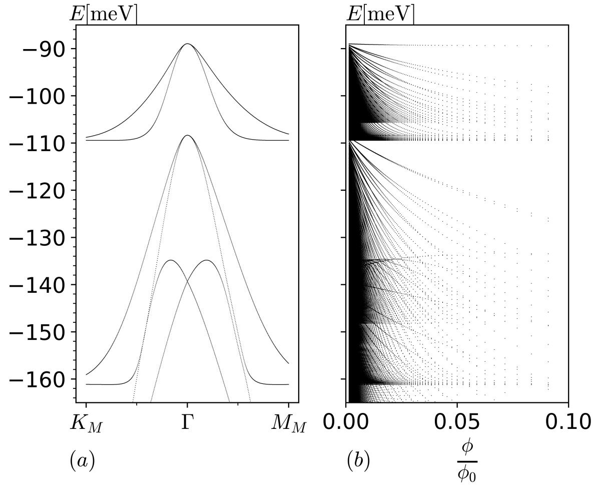

Supplementary Figure 12. The (b) interacting heavy fermion Hofstadter spectrum for KIVC state at $\nu = -2$ spin $\uparrow$ sector contrasted with (a) corresponding zero-field spectrum at $w_0/w_1 = 0.7$. $m_{max} = \lceil \frac{q-3}{2} \rceil$.

familiar form as

$$\tilde{H}^{\nu=0,s=\uparrow,\downarrow}_{\mathbf{0},\text{KIVC}}(\mathbf{k}) = \begin{pmatrix} 0 & v_\star(k_x\sigma_0\tau_z + ik_y\sigma_z\tau_0) & e^{-\frac{\mathbf{k}^2\lambda^2}{2}}(\gamma\sigma_0\tau_0 + v'_\star(k_x\sigma_x\tau_z + k_y\sigma_y\tau_0)) \\ & -\frac{J}{2}\sigma_0\tau_z - M\sigma_z\tau_x & 0 \\ h.c. & & -\frac{U_1}{2}\sigma_0\tau_z \end{pmatrix}, \tag{292}$$

If we were to set $M = 0$ the two valleys in the above Hamiltonian decouple (the model becomes identical to that for VP state due to the $U(4)$ symmetry in flat band limit [2]). Using the results in Sections.(Supplementary note 6 A) and (Supplementary note 7 A), we know how to promote each of the valley block to $\mathbf{B} \neq 0$. The role of M is to couple both the valleys. Hence the Hofstadter spectrum for the coupled modes of c and $\bar{f}$, for the KIVC state at $\nu = 0$, can be obtained by solving the eigenvalues of the operator

$$\hat{h}^{\text{KIVC},\nu=0}_{\alpha,\alpha'} = \begin{pmatrix} \hat{h}^{+1,\nu=0} & \hat{h}_M \\ \hat{h}_M & \hat{h}^{-1,\nu=0} \end{pmatrix}_{\alpha,\alpha'}, \tag{293}$$

The intra-valley operator $\hat{h}^{\tau,\nu=0}$ can be obtained by setting $M = 0$ in operators given in Eqs.(206) and (257) for $\tau = +1$ and -1, respectively. The inter-valley part is given as

$$\hat{h}_M = \begin{pmatrix} 0 & 0 & 0 \\ & -M\sigma_z & 0 \\ h.c. & & 0 \end{pmatrix} \tag{294}$$

The Hofstadter spectrum is shown in Fig.(11).

B. $\nu = -2$

1. *Spin* $\uparrow$

For filling $\nu = -2$, in this section we discuss the Landau quantization of spin flavor $s = \uparrow$ of the $\mathbf{B} = 0$ one-shot HF MF Hamiltonian obtained for the parent KIVC state The $\mathbf{B} = 0$ Hamiltonian upto a $U(4)$ rotation (($e^{i\frac{\pi}{2}\hat{\Sigma}_{x0}}$, where $\hat{\Sigma}_{x0}$ is defined in Eq.(4) of Ref.[2])) can be given as[2]

$$H^{\nu=-2\uparrow}_{\mathbf{0},\text{KIVC}}(\mathbf{k}) = \begin{pmatrix} -2W_1 & v_\star(k_x\sigma_0\tau_z + ik_y\sigma_z\tau_0) & e^{-\frac{\mathbf{k}^2\lambda^2}{2}}(\gamma\sigma_0\tau_0 + v'_\star(k_x\sigma_x\tau_z + k_y\sigma_y\tau_0)) \\ & -2W_3\sigma_0\tau_0 - \frac{J}{2}\sigma_0\tau_z - M\sigma_z\tau_x & 0 \\ h.c. & & -(2U_1 + 12U_2)\sigma_0\tau_0 - \frac{U_1}{2}\sigma_0\tau_z \end{pmatrix}, \tag{295}$$

where $\mathbf{k} = (k_x, k_y) \in$moiré BZ, the Pauli matrices σ and τ act in the orbital and valley space respectively and h.c. represents hermitian conjugate. The intra-valley terms in Eq.(295) can be promoted to $\mathbf{B} \neq 0$ using the results in

Sections.(Supplementary note 6 D) and (Supplementary note 7 C). The two valleys can then be coupled by M. Hence the Hofstadter spectrum for the coupled modes of c and $\bar{f}$, for the KIVC state at $\nu = -2$ spin sector $\uparrow$, can be obtained by solving the eigenvalues of the operator

$$\hat{h}^{\text{KIVC},\uparrow,\nu=-2}_{\alpha,\alpha'} = \begin{pmatrix} \hat{h}^{+1,\nu=-2,\uparrow} & \hat{h}_M \\ \hat{h}_M & \hat{h}^{-1,\nu=-2,\uparrow} \end{pmatrix}_{\alpha,\alpha'}, \tag{296}$$

where the operator $\hat{h}^{\tau,\nu=-2,\uparrow}$ can be obtained by setting $M = 0$ and $s =\uparrow$ in operators given in Eqs.(244) and (284) for $\tau = +1$ and -1, respectively. The inter-valley coupling $\hat{h}_M$ is given in Eq.(294). As argued in the previous section, the decoupled $\bar{f}$ modes give rise to the $2q - (2m_{max} + 3)$ **B** independent energy levels at $-12U_2 - 2U_1 \pm \frac{U_1}{2}$, which physically are linear combinations $\frac{1}{\sqrt{2}}\left(\bar{f}_{11kr's} \pm \bar{f}_{2-1kr's}\right)$ and $\frac{1}{\sqrt{2}}\left(\bar{f}_{21kr's} \pm \bar{f}_{1-1kr's}\right)$, with $r' \geq m_{max} + 1$ and $r' \geq m_{max}$, respectively. The Hofstadter spectrum is shown in Fig.(12b)

Supplementary note 10. EFFECTIVE HAMILTONIANS FOR LANDAU QUANTIZATION OF LIGHT MODES

In order to better understand the Landau quantization of the zero **B** bands of interacting THFM in vicinity of $\Gamma \in$mBZ, i.e. where the lowest energy single particle excitations (light modes) reside, we derive an effective low energy Hamiltonian for THFM at $\mathbf{B} \neq 0$ in this section. Such an analysis offers us with a deeper qualitative understanding of not only the low energy but also the low **B** physics of THFM. As discussed in the main text, the decoupled $\bar{f}$ modes are only responsible for forming the **B** independent higher energy level (heavy modes) and it is rather the coupled modes of c and f fermions which dictate the Hofstadter spectrum of THFM. Thus the results obtained for coupled modes in previous sections serves as the starting point for the following analysis.

A. CNP

In the flat band limit $M = 0$, THFM is U(4) symmetric[2]. The coupled modes at CNP can be described by 6×6 operators given in Eq.(206) and Eq.(257) with M set to zero, for $\tau = \pm 1$ respectively. The magnetic subbands within the narrow bands emanate out of the $\mathbf{B} \to 0$ energy $\mp J/2$, while the remote subbands emanate out of $\mathbf{B} \to 0$ energy $-\tau U_1/4 - \sqrt{U_1^2/16 + \gamma^2}$ and $-\tau U_1/4 + \sqrt{U_1^2/16 + \gamma^2}$, for $\tau = \pm 1$ and marked by $\pm \mathcal{E}_{\mp\tau}$ in main text Fig.(2). These energies correspond to the eigenvalues of the $\mathbf{B} = 0$ flat band THFM at Γ obtained for the parent VP state. Recall that it has the form[2] $\begin{pmatrix} F^{\tau=+1} & \\ & F^{\tau=-1} \end{pmatrix}$ at Γ, where $F^\tau = \begin{pmatrix} 0 & 0 & \gamma\sigma_0 \\ & -\tau J/2\sigma_0 & 0 \\ h.c. & & -\tau U_1/2\sigma_0 \end{pmatrix}$, where Pauli matrix σ_0 acts in orbital space. The corresponding eigenstates, labelled by $|\rho, j, \tau\rangle$ with $\rho \in \{1, 2, 3\}$ and $j \in \{1, 2\}$ are given as

$$|1, 1, \tau = +1\rangle = (0, 0, 0, 1, 0, 0, 0_{6\times1})^T\,; \qquad |1, 1, \tau = -1\rangle = (0_{6\times1}, 0, 0, 0, 1, 0, 0)^T \tag{297}$$

$$|1, 2, \tau = +1\rangle = (0, 0, 1, 0, 0, 0, 0_{6\times1})^T\,; \qquad |1, 2, \tau = -1\rangle = (0_{6\times1}, 0, 0, 1, 0, 0, 0)^T \tag{298}$$

$$|2, 1, \tau = +1\rangle = \frac{1}{\sqrt{N_{X1}}}(0, X_1, 0, 0, 0, 1, 0_{6\times1})^T\,; \quad |2, 1, \tau = -1\rangle = \frac{1}{\sqrt{N_{X-1}}}(0_{6\times1}, 0, X_{-1}, 0, 0, 0, 1)^T \tag{299}$$

$$|2, 2, \tau = +1\rangle = \frac{1}{\sqrt{N_{X1}}}(X_1, 0, 0, 0, 1, 0, 0_{6\times1})^T\,; \quad |2, 2, \tau = -1\rangle = \frac{1}{\sqrt{N_{X-1}}}(0_{6\times1}, X_{-1}, 0, 0, 0, 1, 0)^T \tag{300}$$

$$|3, 1, \tau = +1\rangle = \frac{1}{\sqrt{N_{Y1}}}(0, Y_1, 0, 0, 0, 1, 0_{6\times1})^T\,; \quad |3, 1, \tau = -1\rangle = \frac{1}{\sqrt{N_{Y-1}}}(0_{6\times1}, 0, Y_{-1}, 0, 0, 0, 1)^T \tag{301}$$

$$|3, 2, \tau = +1\rangle = \frac{1}{\sqrt{N_{Y1}}}(Y_1, 0, 0, 0, 1, 0, 0_{6\times1})^T\,; \quad |3, 2, \tau = -1\rangle = \frac{1}{\sqrt{N_{Y-1}}}(0_{6\times1}, Y_{-1}, 0, 0, 0, 1, 0)^T \tag{302}$$

where $(0_{6\times1})$ represent 6 zero entries $(0, \ldots, 0)$,

$$X_\tau = -\frac{-\tau U_1/4 + \sqrt{U_1^2/16 + \gamma^2}}{\gamma}, \tag{303}$$

$$Y_\tau = -\frac{-\tau U_1/4 - \sqrt{U_1^2/16 + \gamma^2}}{\gamma}, \tag{304}$$

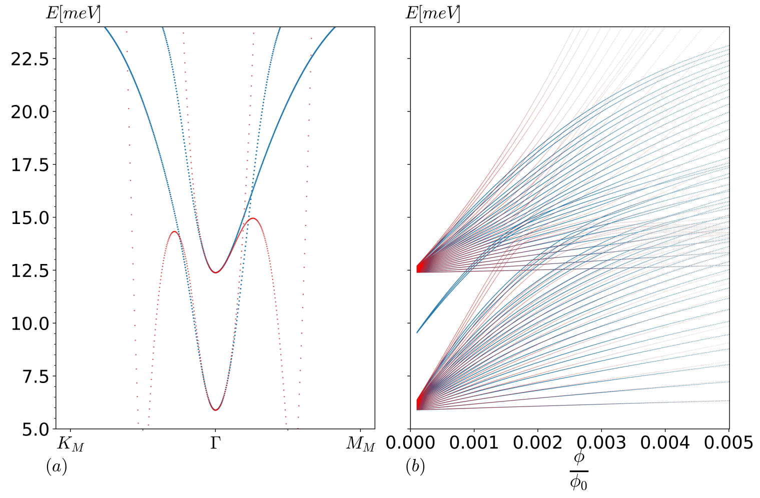

Supplementary Figure 13. The comparison of (b) LL spectrum obtained using the effective Hamiltonian (·) with the exact calculation (·) contrasted with the corresponding comparison at (a) $\mathbf{B}=0$ for valley $\mathbf{K}'$ at CNP. The spectrum at valley $\mathbf{K}$ is related by particle hole symmetry. For the comparison to be tractable we set $m_{max}=26$. Note that the above figure is spin degenerate.

The normalizations $N_{X\tau}=1+X_\tau^2$ and $N_{Y,\tau}=1+Y_\tau^2$. The energy of state $|\rho,j,\tau\rangle$ can be labelled as $E_{\rho,j,\tau}$, where

$$E_{1j\tau}=-\tau J/2\equiv E_\tau, \tag{305}$$

$$E_{2j\tau}=-\tau U_1/4-\sqrt{U_1^2/16+\gamma^2}\equiv E_{X\tau} \tag{306}$$

$$E_{3j\tau}=-\tau U_1/4+\sqrt{U_1^2/16+\gamma^2}\equiv E_{Y\tau}. \tag{307}$$

As shown in the section(Supplementary note 8), the $\mathbf{B}\neq 0$ THFM for the coupled modes, upto the ambiguity of correct ordering of singular values, is the same as having naively minimally substituted in $\mathbf{B}=0$ THFM. Hence, throughout the discussion we consider the $\mathbf{B}\neq 0$ basis to be the finite $\mathbf{B}$ $\mathbf{k}\cdot\mathbf{p}$ basis, i.e. given as $|\rho,j,\tau,m\rangle=|\rho,j,\tau\rangle|m\rangle$, where $|m\rangle$ is the mth LL.

1. *Parent Valley Polarized State*

Clearly the subspace of interest, for which we want to derive an effective Hamiltonian, is the one spanned by states $|\rho=1,j,\tau,m\rangle$, i.e. the $a=\{3,4\}$ c fermions for each $\tau=\pm1$. To obtain the effective Hamiltonian we eliminate all the terms in operator Eqs.(206) and (257) (i.e. at $\tau=+1,-1$ respectively) which can mix the subspaces using Schrieffer Wolff Transformation (SWT). We moreover omit the contributions to effective Hamiltonian of order greater than $B^{3/2}$, i.e. $1/\ell^3$. Below, we discuss the SWT scheme followed to obtain the effective Hamiltonian. We first rewrite the operators in Eqs.(206) and (257) with respect to the $\mathbf{B}=0$ eigenstates at Γ, $|\rho,j,\tau\rangle$, given in Eqs.(297)-(302) as

$$H=H_0+\Delta V, \tag{308}$$

where Δ is an artificial parameter that helps us keeping track of the order in perturbation, to which we compute the effective Hamiltonian and is later set to 1. Along with M, we treat all the terms in operator in Eqs.(206) and (257) (i.e. at $\tau=+1,-1$ respectively) which can mix the subspaces as perturbation V. The unperturbed part $H_0=\begin{pmatrix} H_0^{\tau=+1} & \\ & H_0^{\tau=-1}\end{pmatrix}$ where

$$H_0^\tau=\begin{pmatrix} E_\tau\sigma_0 & 0 & 0\\ 0 & E_{X\tau}\sigma_0 & 0\\ 0 & 0 & E_{Y\tau}\sigma_0\end{pmatrix}. \tag{309}$$

The Pauli matrix σ_0 above acts in the $j \in \{1,2\}$ space of for each ρ, and the energies E, $E_{X\tau}$, $E_{Y\tau}$ are given in Eqs.(305)-(307). The perturbation is given as $V = \begin{pmatrix} V^{\tau=+1} & 0 \\ 0. & V^{\tau=-1} \end{pmatrix}$, where

$$V^{\tau=+1} = \begin{pmatrix} 0 & M & -i\frac{\sqrt{2}}{\ell}\frac{X_1 v_\star}{\sqrt{N_{X1}}}a & 0 & -i\frac{\sqrt{2}}{\ell}\frac{Y_1 v_\star}{\sqrt{N_{Y1}}}a & 0 \\ & 0 & 0 & i\frac{\sqrt{2}}{\ell}\frac{X_1 v_\star}{\sqrt{N_{X1}}}a^\dagger & 0 & i\frac{\sqrt{2}}{\ell}\frac{Y_1 v_\star}{\sqrt{N_{Y1}}}a^\dagger \\ & & 0 & -i\frac{2\sqrt{2}}{\ell}\frac{X_1 v'_\star}{N_{X1}}a & 0 & -i\frac{\sqrt{2}}{\ell}\frac{(X_1+Y_1)v'_\star}{\sqrt{N_{X1}N_{Y1}}}a \\ & & & 0 & i\frac{\sqrt{2}}{\ell}\frac{(X_1+Y_1)v'_\star}{\sqrt{N_{X1}N_{Y1}}}a^\dagger & 0 \\ & & & & 0 & -i\frac{2\sqrt{2}}{\ell}\frac{Y_1 v'_\star}{N_{Y1}}a \\ h.c. & & & & & 0 \end{pmatrix}, \tag{310}$$

where $h.c.$ represents hermitian conjugate. In above matrix, the terms of $O(\frac{1}{\ell^2})$ have been opted out as they contribute terms of $O(\frac{1}{\ell^4})$ to the effective Hamiltonian. $V^{\tau=-1}$ can be obtained by replacing $X_1, Y_1 \to X_{-1}, Y_{-1}$, $N_{X1}, N_{Y1} \to N_{X-1}, N_{Y-1}$ and $a \leftrightarrow a^\dagger$ in Eq.(310). Let us now consider the SWT, generated by $S = S^\dagger$, such that it decouples the three subspaces in the transformed Hamiltonian. The transformed Hamiltonian is given as

$$H' = e^{iS}He^{-iS} \tag{311}$$

$$= H + i[S,H] + \frac{i^2}{2!}[S,[S,H]] + \frac{i^3}{3!}[S,[S,[S,H]]] + \dots. \tag{312}$$

The generator of the transformation can be expanded in orders of Δ as

$$S = \Delta S_1 + \Delta^2 S_2 + \Delta^3 S_3 + \dots \tag{313}$$

Substituting Eq.(313) into Eq.(312) gives us

$$H' = H_0 + \Delta\left(V + i[S_1,H_0]\right) + \Delta^2\left(i[S_2,H_0] + i[S_1,V] + \frac{i^2}{2!}[S_1,[S_1,H_0]]\right) + \tag{314}$$

$$\Delta^3\left(i[S_3,H_0] + i[S_2,V] + \frac{i^2}{2!}[S_2,[S_1,H_0]] + \frac{i^2}{2!}[S_1,[S_2,H_0]] + \frac{i^2}{2!}[S_1,[S_1,V]]] + \frac{i^3}{3!}[S_1,[S_1,[S_1,H_0]]]\right) \tag{315}$$

$$+O(\Delta^4) + \dots \tag{316}$$

$$= H_0 + \Delta H'_1 + \Delta^2 H'_2 + \Delta^3 H'_3 + O(\Delta^4) + \dots \tag{317}$$

Recall that the generator S is defined by the condition that the SWT decouples the three subspaces. Since the two valleys are decoupled, for either valley τ, we have

$$\langle \rho, j, \tau, m | H'_n | \rho', j', \tau, m' \rangle = \delta_{\rho,\rho'} \langle \rho, j, \tau, m | H'_n | \rho, j', \tau, m' \rangle. \tag{318}$$

We thus have, for $\rho \neq \rho'$ and $n = 1$ in above

$$\langle \rho, j, \tau, m | \left(V + i[S_1,H_0]\right) | \rho', j', \tau, m' \rangle = 0 \tag{319}$$

$$\implies \langle \rho, j, \tau, m | S_1 | \rho', j', \tau, m' \rangle = \begin{cases} i\frac{\langle \rho,j,\tau,m|V|\rho',j',\tau,m'\rangle}{E_{\rho'j'\tau} - E_{\rho j\tau}} & \text{for} \quad \rho \neq \rho', \\ 0 & \text{for} \quad \rho = \rho', \end{cases} \tag{320}$$

Similarly, for $\rho \neq \rho'$ and $n = 2$, we have

$$\langle \rho, j, \tau, m | \left(i[S_2,H_0] + i[S_1,V] + \frac{i^2}{2!}[S_1,[S_1,H_0]]\right) | \rho', j', \tau, m' \rangle = 0 \tag{321}$$

$$\implies \langle \rho, j, \tau, m | S_2 | \rho', j', \tau, m' \rangle = \frac{1}{E_{\rho j\tau} - E_{\rho' j'\tau}} \left(\sum_{\tilde{\rho}\neq\rho;\tilde{j};\tilde{m}} \langle \rho, j, \tau, m | S_1 | \tilde{\rho}, \tilde{j}, \tau, \tilde{m} \rangle \langle \tilde{\rho}, \tilde{j}, \tau, \tilde{m} | V | \rho', j', \tau, m' \rangle - \right.$$

$$\sum_{\tilde{\rho}\neq\rho';\tilde{j};\tilde{m}} \langle \rho, j, \tau, m | V | \tilde{\rho}, \tilde{j}, \tau, \tilde{m} \rangle \langle \tilde{\rho}, \tilde{j}, \tau, \tilde{m} | S_1 | \rho', j', \tau, m' \rangle$$

$$\left. - \sum_{\tilde{\rho}\neq\rho\rho';\tilde{j},\tilde{m}} \left(E_{\tilde{\rho}\tilde{j}\tau} - \frac{E_{\rho j\tau} + E_{\rho' j'\tau}}{2} \right) \langle \rho, j, \tau, m | S_1 | \tilde{\rho}, \tilde{j}, \tau, \tilde{m} \rangle \langle \tilde{\rho}, \tilde{j}, \tau, \tilde{m} | S_1 | \rho', j', \tau, m' \rangle \right) \text{for} \quad \rho \neq \rho',$$

$$= 0 \quad \text{for} \quad \rho = \rho', \tag{322}$$

were $\tilde{j}$ is summed over $\{1,2\}$, i.e. the two states constituting each subspace. We further assume that

$$\sum_{\tilde{m}} |\tilde{\rho},\tilde{j},\tau,\tilde{m}\rangle\langle\tilde{\rho},\tilde{j},\tau,\tilde{m}| = |\tilde{\rho},\tilde{j},\tau\rangle\langle\tilde{\rho},\tilde{j},\tau|, \tag{323}$$

which is justified in the $(\mathbf{k}\cdot\mathbf{p})$ continuum limit, as the upper cutoff on LLs in such a limit is unbounded. The effective Hamiltonian for either subspace ρ, can now be deduced in orders of Δ. The $O(\Delta^0)$ contribution to the effective Hamiltonian in subspace ρ is trivially $E_{\rho j\tau}\delta_{jj'}$ and $O(\Delta^1)$ terms are

$$\langle\rho,j,\tau,m|H_1'|\rho,j',\tau,m'\rangle = \langle\rho,j,\tau,m|V|\rho,j',\tau,m'\rangle. \tag{324}$$

The $O(\Delta^2)$ terms are found out to be

$$\langle\rho,j,\tau,m|H_2'|\rho,j',\tau,m'\rangle = \frac{1}{2}\sum_{\tilde{\rho}\neq\rho;\tilde{j}}\langle\rho,j,\tau,m|V|\tilde{\rho},\tilde{j},\tau\rangle\langle\tilde{\rho},\tilde{j},\tau|V|\rho,j',\tau,m'\rangle\left(\frac{1}{E_{\rho j\tau}-E_{\tilde{\rho}\tilde{j}\tau}}+\frac{1}{E_{\tilde{\rho}\tilde{j}\tau}-E_{\rho j'\tau}}\right). \tag{325}$$

The $O(\Delta^3)$ terms are found out to be

$$\langle\rho,j,\tau,m|H_3'|\rho,j',\tau,m'\rangle = \frac{i}{2}\sum_{\tilde{\rho}\neq\rho;\tilde{j}}\left[\langle\rho,j,\tau,m|S_2|\tilde{\rho},\tilde{j},\tau\rangle\langle\tilde{\rho},\tilde{j},\tau|V|\rho,j',\tau,m'\rangle-\langle\rho,j,\tau,m|V|\tilde{\rho},\tilde{j},\tau\rangle\langle\tilde{\rho},\tilde{j},\tau|S_2|\rho,j',\tau,m'\rangle\right]$$
$$-\frac{1}{12}\sum_{\tilde{\rho}\neq\rho;\tilde{j}}\sum_{\bar{\rho}\neq\rho\tilde{\rho};\bar{j}}\left[\langle\rho,j,\tau,m|S_1|\bar{\rho},\bar{j},\tau\rangle\langle\bar{\rho},\bar{j},\tau|S_1|\tilde{\rho},\tilde{j},\tau\rangle\langle\tilde{\rho},\tilde{j},\tau|V|\rho,j',\tau,m'\rangle+\right.$$
$$\left.\langle\rho,j,\tau,m|V|\bar{\rho},\bar{j},\tau\rangle\langle\bar{\rho},\bar{j},\tau|S_1|\tilde{\rho},\tilde{j},\tau\rangle\langle\tilde{\rho},\tilde{j},\tau|S_1|\rho,j',\tau,m'\rangle-2\langle\rho,j,\tau,m|S_1|\bar{\rho},\bar{j},\tau\rangle\langle\bar{\rho},\bar{j},\tau|V|\tilde{\rho},\tilde{j},\tau\rangle\langle\tilde{\rho},\tilde{j},\tau|S_1|\rho,j',\tau,m'\rangle\right] \tag{326}$$

Substituting Eqs.(320) and (322) in Eqs.(325) and (326) and further substituting them and Eq.(309) in Eq.(317), upto $O(\Delta^3)$, the effective Hamiltonian for $\rho=1$ is given as

$$H^{eff} = \begin{pmatrix} H_{VP}^{\tau=+1} & 0 \\ 0 & H_{VP}^{\tau=-1}\end{pmatrix}, \tag{327}$$

where

$$H^{\tau=+1} = \begin{pmatrix} -\frac{J}{2}+\hbar\omega_c^{(1)}aa^\dagger & i\frac{A^{(1)}}{\ell^3}a^3 \\ -i\frac{A^{(1)}}{\ell^3}a^{\dagger 3} & -\frac{J}{2}+\hbar\omega_c^{(1)}a^\dagger a\end{pmatrix} + M\left(1+\frac{M_c^{(1)}}{\ell^2}(aa^\dagger+a^\dagger a)\right)\sigma_x, \tag{328}$$

where the Pauli matrix acts in the orbital space of $a\in\{4,3\}$ c-fermions. The cyclotron frequency ω_c^τ and the other coefficients above are

$$\hbar\omega_c^{(\tau)} = \frac{2v_\star^2}{\ell^2}\left(\frac{X_\tau^2}{N_{X\tau}(E_\tau-E_{X\tau})}+\frac{Y_\tau^2}{N_{Y\tau}(E_\tau-E_{Y\tau})}\right), \tag{329}$$

$$A^{(\tau)} = 4\sqrt{2}v_\star^2 v_\star'\left(\frac{X_\tau Y_\tau(X_\tau+Y_\tau)}{N_{X\tau}N_{Y\tau}(E_\tau-E_{X\tau})(E_\tau-E_{Y\tau})}+\frac{X_\tau^3}{N_{X\tau}^2(E_\tau-E_{X\tau})^2}+\frac{Y_\tau^3}{N_{Y\tau}^2(E_\tau-E_{Y\tau})^2}\right), \tag{330}$$

$$M_c^{(\tau)} = -v_\star^2\left(\frac{X_\tau^2}{N_{X\tau}(E_\tau-E_{X\tau})^2}+\frac{Y_\tau^2}{N_{Y\tau}(E_\tau-E_{Y\tau})^2}\right). \tag{331}$$

$H_{VP}^{\tau=-1}$ can be obtained by replacing $a\leftrightarrow a^\dagger$, $\omega_c^{(1)},A^{(1)},M_c^{(1)}\to\omega_c^{(-1)},A^{(-1)},M_c^{(-1)}$ and $-\frac{J}{2}\to\frac{J}{2}$ in Eq.(328). Substituting Eqs.(303)-(307) in the coefficients, we have $\ell^2\hbar\omega_c^{(\tau)}=-\tau(399586.49352)$meVÅ^2, $A^{(\tau)}=-\tau(42668446.86852)$ meVÅ^3 and $M_c^{(\tau)}=-12847.14767$Å^2.

The above effective Hamiltonians H^τ describe the Landau quantization of the light single particle excitations towards CNP obtained for parent VP state. Clearly, the LLs emanate out of $-\tau J/2\pm M$. For the U(4) symmetric THFM (i.e. $M=0$), in the $\mathbf{B}\to 0$ limit, we can the drop off-diagonal $O(\ell^{-3})$ terms in H^τ. Then apart from the mode $(0,|0\rangle)^T$ and $(|0\rangle,0)^T$ at $\tau=+1$ and -1, respectively, all other LLs come in degenerate pair of two. This degeneracy at $\mathbf{B}\to 0$ limit gets split as we tune back M. The comparison of the LL spectrum obtained using the above effective Hamiltonian with the one obtained via the exact calculation for valley sector $\mathbf{K'}$ is shown in Fig.(13). The LL basis used to generate the plot is $(0,|m_1\rangle,|m_1\rangle,0)^T$, $(|m_2\rangle,|m_2+3\rangle,|m_2+3\rangle,|m_2\rangle)^T$ and $(|m_3\rangle,0,0,|m_3\rangle)^T$, where $m_1\in\{0,1,2\}$, $m_2\in\{0,\ldots,m_{max}-4\}$ and $m_3\in\{m_{max}-3,m_{max}-2,m_{max}-1\}$ respectively. We use $(|m_3\rangle,0,0,|m_3\rangle)^T$ rather than $(|m_3\rangle,|m_3+3\rangle,|m_3+3\rangle,|m_3\rangle)^T$ in order to avoid three modes emanating out of spurious $\mathbf{B}\to 0$ energy $-\tau J/2$, for each τ. These three spurious modes are present in the exact calculation, as can be seen in Fig.(13). However, recall that in practice we use $m_{max}=\lceil\frac{q-3}{2}\rceil$, so these three LLs are lost as $\mathbf{B}(q)$ increases(decreases).

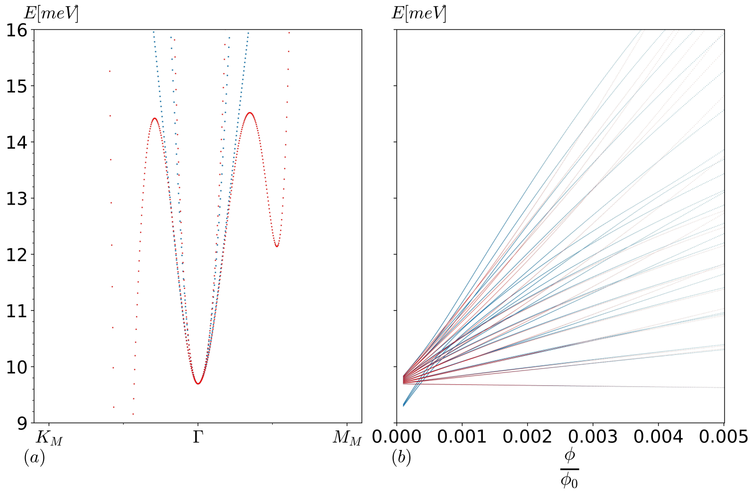

Supplementary Figure 14. The comparison of (b) LL spectrum obtained using the effective Hamiltonian (•) with the exact calculation (•) contrasted with the corresponding comparison at (a) $\mathbf{B} = 0$ for KIVC state above CNP. The spectrum below CNP is related by particle hole symmetry. For the comparison to be tractable we set $m_{max} = 11$. Note that the above figure is spin degenerate.

2. *Parent Kramers Intervalley Coherent State*

In the case of the parent Kramers intervalley coherent (KIVC) state, the coupled c and f modes are described by the operator in Eq.(293). Unlike in previous section, we now break the $\mathbf{B} = 0$ Hilbert space at Γ into 5 subspaces. The subspace of interest is the one spanned by states $|1, j, \tau\rangle$ with $j \in \{1, 2\}$ and $\tau = \pm 1$, i.e. the one spanned by $a = \{3, 4\}$ c fermions at each valley. The remaining four subspaces are spanned by $|2, j, +1\rangle$, $|2, j, -1\rangle$, $|3, j, +1\rangle$ and $|3, j, -1\rangle$, where $j \in \{1, 2\}$. These states are given in Eqs.(297)-(302). In the following discussion, we refer to these states and their energies by $|\epsilon, l\rangle$ and $E_{\epsilon l}$, respectively. Here $\epsilon \in \{1, \ldots, 5\}$ labels the 5 subspaces. The index $l \in \{1, \ldots, 4\}$ for $\epsilon = 1$ and $l \in \{1, 2\}$ for $\epsilon = \{3, 4, 5\}$, labels the states spanning the subspace ϵ. We promote these states to $\mathbf{B} \neq 0$ as $|\epsilon, l, m\rangle = |\epsilon, l\rangle|m\rangle$, where $|m\rangle$ is the mth LL, i.e. the finite $\mathbf{B}$ $\mathbf{k}\cdot\mathbf{p}$ basis. Using the SWT procedure introduced in the previous section, we eliminate the terms in the operator given in Eq.(293) which can mix between these subspaces to obtain an effective Hamiltonian for the subspace spanned by by states $|\epsilon = 1, l, m\rangle$.

To begin with, we re-write the operator in Eq.(293) with respect to the above $\mathbf{B} = 0$ eigenstates at Γ as

$$H = H_0 + \Delta V, \tag{332}$$

where $H_0 = \begin{pmatrix} H_0^{\tau=+1} & \\ & H_0^{\tau=-1} \end{pmatrix}$ and $V = \begin{pmatrix} \bar{V}^{\tau=+1} & V_M \\ h.c. & \bar{V}^{\tau=-1} \end{pmatrix}$. H_0^τ is given in Eq.(309). The intra-valley perturbation $\bar{V}^{\tau=+1}$ can be obtained by setting $M = 0$ in Eq.(310). We can obtain $\bar{V}^{\tau=-1}$ by replacing $X_1, Y_1 \to X_{-1}, Y_{-1}$, $N_{X1}, N_{Y1} \to N_{X-1}, N_{Y-1}$ and $a \leftrightarrow a^\dagger$ in $V^{\tau=+1}$. The inter-valley perturbation V_M is given as

$$V_M = \begin{pmatrix} M\sigma_z & 0 & 0 \\ 0 & 0 & 0 \\ 0 & 0 & 0 \end{pmatrix} \tag{333}$$

The effective Hamiltonian can then be expanded in orders of Δ, as given in Eq.(317). Following the condition that the transformation decouples each subspace, we have

$$\langle \epsilon, l, m|H'_n|\epsilon', l', m'\rangle = \delta_{\epsilon,\epsilon'}\langle \epsilon, l, m|H'_n|\epsilon, l', m'\rangle \tag{334}$$

For $\epsilon \neq \epsilon'$ and substituting $n = 1$ in H'_n gives

$$\langle \epsilon, l, m| \left(V + i[S_1, H_0]\right)|\epsilon', l', m'\rangle = 0 \tag{335}$$

$$\implies \langle \epsilon, l, m|S_1|\epsilon', l', m'\rangle = \begin{cases} i\frac{\langle \epsilon, l, m|V|\epsilon', l', m'\rangle}{E_{\epsilon' l'} - E_{\epsilon l}} & \text{for } \epsilon \neq \epsilon', \\ 0 & \text{for } \epsilon = \epsilon', \end{cases} \tag{336}$$

For $\epsilon \neq \epsilon'$ and substituting $n = 2$ in H'_n gives

$$\langle \epsilon, l, m| \left(i[S_2, H_0] + i[S_1, V] + \frac{i^2}{2!}[S_1, [S_1, H_0]] \right) |\epsilon', l', m'\rangle = 0 \tag{337}$$

$$\implies \langle \epsilon, l, m|S_2|\epsilon', l', m'\rangle = \frac{1}{E_{\epsilon l} - E_{\epsilon' l'}} \left(\sum_{\tilde{\epsilon}\neq\epsilon;\tilde{l};\tilde{m}} \langle \epsilon, l, m|S_1|\tilde{\epsilon}, \tilde{l}, \tilde{m}\rangle\langle\tilde{\epsilon}, \tilde{l}, \tilde{m}|V|\epsilon', l', m'\rangle - \right.$$
$$\sum_{\tilde{\epsilon}\neq\epsilon';\tilde{l};\tilde{m}} \langle \epsilon, l, m|V|\tilde{\epsilon}, \tilde{l}, \tilde{m}\rangle\langle\tilde{\epsilon}, \tilde{l}, \tilde{m}|S_1|\epsilon', l', m'\rangle$$
$$\left. - \sum_{\tilde{\epsilon}\neq\epsilon\epsilon';\tilde{l},\tilde{m}} \left(E_{\tilde{\epsilon}\tilde{l}} - \frac{E_{\epsilon l} + E_{\epsilon' l'}}{2} \right) \langle \epsilon, l, m|S_1|\tilde{\epsilon}, \tilde{l}, , \tilde{m}\rangle\langle\tilde{\epsilon}, \tilde{l}, \tilde{m}|S_1|\epsilon', l', m'\rangle \right) \text{for} \quad \epsilon \neq \epsilon',$$
$$= 0 \quad \text{for} \quad \epsilon = \epsilon', \tag{338}$$

As in the previous section, we further assume that

$$\sum_{\tilde{m}} |\tilde{\epsilon}, \tilde{l}, \tilde{m}\rangle\langle\tilde{\epsilon}, \tilde{l}, \tilde{m}| = |\tilde{\epsilon}, \tilde{l}\rangle\langle\tilde{\epsilon}, \tilde{l}|, \tag{339}$$

which is justified in the $(\mathbf{k}\cdot\mathbf{p})$ continuum limit, as the upper cutoff on LLs in such a limit is unbounded. Substituting Eqs.(336) and (338) into the expansion in Eq.(317), we can now obtain the effective Hamiltonian in orders of Δ. The $O(\Delta^0)$ contribution to the effective Hamiltonian in subspace ϵ is trivially $E_{\epsilon l}\delta_{ll'}$ and $O(\Delta^1)$ terms are

$$\langle \epsilon, l, m|H'_1|\epsilon, l', m'\rangle = \langle \epsilon, l, m|V|\epsilon, l', m'\rangle. \tag{340}$$

The $O(\Delta^2)$ terms are found out to be

$$\langle \epsilon, l, m|H'_2|\epsilon, l', m'\rangle = \frac{1}{2}\sum_{\tilde{\epsilon}\neq\epsilon;\tilde{l}} \langle \epsilon, l, m|V|\tilde{\epsilon}, \tilde{l}\rangle\langle\tilde{\epsilon}, \tilde{l}|V|\epsilon, l', m'\rangle \left(\frac{1}{E_{\epsilon l} - E_{\tilde{\epsilon}\tilde{l}}} + \frac{1}{E_{\tilde{\epsilon}\tilde{l}} - E_{\epsilon l'}} \right). \tag{341}$$

The $O(\Delta^3)$ terms are found out to be

$$\langle \epsilon, l, m|H'_3|\epsilon, l', m'\rangle = \frac{i}{2}\sum_{\tilde{\epsilon}\neq\epsilon;\tilde{l}} \left[\langle \epsilon, l, m|S_2|\tilde{\epsilon}, \tilde{l}\rangle\langle\tilde{\epsilon}, \tilde{l}|V|\epsilon, l', m'\rangle - \langle \epsilon, l, m|V|\tilde{\epsilon}, \tilde{l}\rangle\langle\tilde{\epsilon}, \tilde{l}|S_2|\epsilon, l', m'\rangle \right]$$
$$-\frac{1}{12}\sum_{\tilde{\epsilon}\neq\epsilon;\tilde{l}}\sum_{\bar{\epsilon}\neq\epsilon\tilde{\epsilon};\bar{l}} \left[\langle \epsilon, l, m|S_1|\bar{\epsilon}, \bar{l}\rangle\langle\bar{\epsilon}, \bar{l}|S_1|\tilde{\epsilon}, \tilde{l}\rangle\langle\tilde{\epsilon}, \tilde{l}|V|\epsilon, l', m'\rangle + \right.$$
$$\left. \langle \epsilon, l, m|V|\bar{\epsilon}, \bar{l}\rangle\langle\bar{\epsilon}, \bar{l}|S_1|\tilde{\epsilon}, \tilde{l}\rangle\langle\tilde{\epsilon}, \tilde{l}|S_1|\epsilon, l', m'\rangle - 2\langle \epsilon, l, m|S_1|\bar{\epsilon}, \bar{l}\rangle\langle\bar{\epsilon}, \bar{l}|V|\tilde{\epsilon}, \tilde{l}\rangle\langle\tilde{\epsilon}, \tilde{l}|S_1|\epsilon, l', m'\rangle \right] \tag{342}$$

Up to the $O(\frac{1}{\ell^3})$, the effective Hamiltonian for subspace $\epsilon = 1$ is found to be

$$H^{eff} = \begin{pmatrix} H^{\tau=+1}_{\text{KIVC}} & H_M \\ h.c. & H^{\tau=-1}_{\text{KIVC}} \end{pmatrix}, \tag{343}$$

where H^{τ}_{KIVC} can be obtained by setting $M = 0$ in the expressions for H^{τ}_{VP} in Eq.(327). The matrix H_M couples both the valleys and is given as

$$H_M = M\left(1 + \frac{M_{c1}}{\ell^2}a^\dagger a + \frac{M_{c2}}{\ell^2}aa^\dagger \right)\sigma_z \tag{344}$$

where the Pauli matrix acts in the orbital space of $a \in \{4, 3\}$ c-fermions and

$$M_{c1} = v_\star^2 \left(\frac{X_{-1}^2}{N_{X-1}(E_1 - E_{X-1})(E_{X-1} - E_{-1})} + \frac{Y_{-1}^2}{N_{Y-1}(E_1 - E_{Y-1})(E_{Y-1} - E_{-1})} \right), \tag{345}$$

$$M_{c2} = v_\star^2 \left(\frac{X_1^2}{N_{X1}(E_1 - E_{X1})(E_{X1} - E_{-1})} + \frac{Y_1^2}{N_{Y1}(E_1 - E_{Y1})(E_{Y1} - E_{-1})} \right). \tag{346}$$

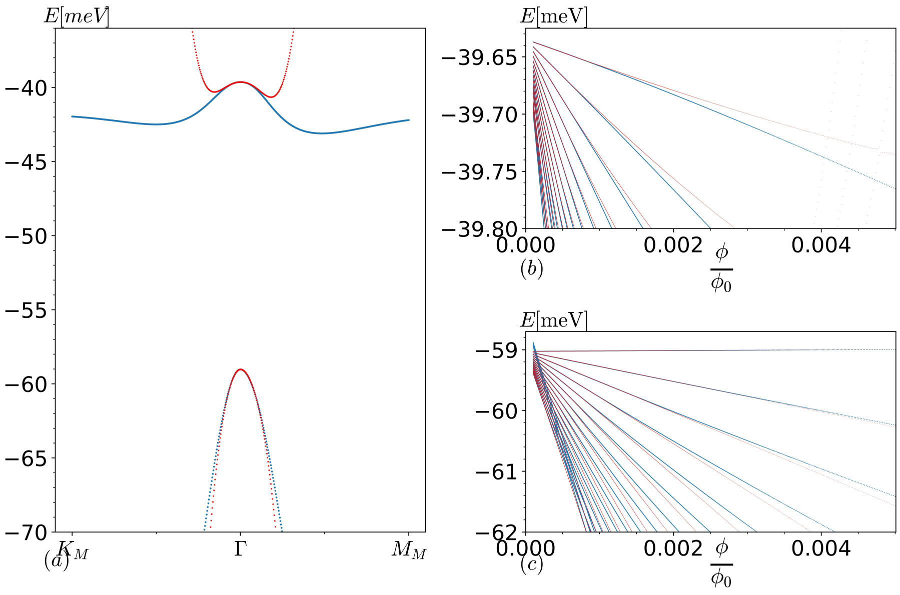

Supplementary Figure 15. The comparison of (b,c) LL spectrum obtained using the effective Hamiltonian (·) with the exact calculation (·) contrasted with the corresponding comparison at (a) $\mathbf{B} = 0$ for valley spin sector $\mathbf{K}\downarrow$ at $\nu = -1$. For the comparison to be tractable we set an upper-cutoff of 25 on the LL index. Note that the above figure is spin degenerate.

Substituting Eqs.(303)-(307) above we get $M_{c1} = M_{c2} = -20880.79644\text{Å}^2$. Clearly the LLs emanate out of $\mathbf{B} \to 0$ energy $\pm\sqrt{M^2 + J^2/4}$. In the $\mathbf{B} \to 0$ limit, we can drop the $\mathrm{O}(\frac{1}{\ell^3})$ terms in the effective Hamiltonian. The LL energies in this limit take the form $\pm E_n^{\pm}$, where $E_n^{\pm} = \sqrt{(\frac{J}{2} + \hbar\omega_c^{(-1)}(n+\frac{1}{2}))^2 + M^2(1 + \frac{M_{c1}}{\ell^2}(2n+1))^2} \pm \frac{\hbar\omega_c^{(-1)}}{2}$, and $n \in \{0,1,2,\dots\}$. For small LL index n, we further have $E_{n+1}^- \approx E_n^+$. Thus except the anomalous mode $\pm E_0^-$, the leading LLs appear in pairs approaching double degeneracy, as can be seen in Fig.(14) where we compare the LL spectrum obtained using the above effective Hamiltonian with the one obtained via the exact calculation. Note that such an approximate degeneracy is absent in the spectrum for VP state (see Fig.(13)).

The LL basis used to generate the plot is $(0, |m_1\rangle, |m_1\rangle, 0)^T$, $(|m_2\rangle, |m_2+3\rangle, |m_2+3\rangle, |m_2\rangle)^T$ and $(|m_3\rangle, 0, 0, |m_3\rangle)^T$, where $m_1 \in \{0,1,2\}$, $m_2 \in \{0,\dots,m_{max}-4\}$ and $m_3 \in \{m_{max}-3, m_{max}-2, m_{max}-1\}$ respectively. We use $(|m_3\rangle, 0, 0, |m_3\rangle)^T$ rather than $(|m_3\rangle, |m_3+3\rangle, |m_3+3\rangle, |m_3\rangle)^T$ in order to avoid three modes emanating out of spurious $\mathbf{B} \to 0$ energy $\pm J/2$. These six spurious modes are present in the exact calculation, as can be seen in Fig.(18). However, recall that in practice we use $m_{max} = \lceil \frac{q-3}{2} \rceil$, so these three LLs are lost as $\mathbf{B}(q)$ increases(decreases).

B. $\nu = -1$

1. *Parent Valley Polarized State*

$\mathbf{K}\downarrow$: In this section we discuss the derivation of the $\mathbf{B} \neq 0$ effective Hamiltonian for the spin and valley sector $\downarrow$, $\mathbf{K}$ for the case of parent VP state at $\nu = -1$. The coupled modes of c and f fermions are described by the operator in Eq.(233). As discussed in main text and Section(Supplementary note 6 C), for the above mean-field sector in flat band limit $M = 0$, the magnetic subbands within the narrow bands emanate out of the $\mathbf{B} \to 0$ energy $-W_3 \mp \frac{J}{2}$. The remote subbands on the other hand emanate out of the $\mathbf{B} \to 0$ energy $\frac{1}{2}\left(-W_1 - (6U_2 + \frac{U_1}{2}) \pm \sqrt{4\gamma^2 + (W_1 - (6U_2 + \frac{U_1}{2}))^2}\right)$ and $\frac{1}{2}\left(-W_1 - (6U_2 + \frac{3U_1}{2}) \pm \sqrt{4\gamma^2 + (W_1 - (6U_2 + \frac{3U_1}{2}))^2}\right)$. These energies correspond to the eigenvalues of the flat band $\mathbf{B} = 0$ THFM at Γ at $\nu = -1$, for the same mean-field sector. Recall that at Γ, it has the form[2] $\begin{pmatrix} -W_1\sigma_0 & 0 & \gamma\sigma_0 \\ & -W_3\sigma_0 - \frac{J}{2}\sigma_z & 0 \\ h.c. & & -(6U_2+U_1)\sigma_0 - \frac{U_1}{2}\sigma_z \end{pmatrix}$, where Pauli matrix σ acts in orbital space. The corresponding

eigenvectors can be listed as

$$|1\rangle = (0,0,1,0,0,0)^T \tag{347}$$
$$|2\rangle = (0,0,0,1,0,0)^T \tag{348}$$
$$|3\rangle = \frac{1}{\sqrt{N_X}}(X,0,0,0,1,0) \tag{349}$$
$$|4\rangle = \frac{1}{\sqrt{N_Y}}(Y,0,0,0,1,0) \tag{350}$$
$$|5\rangle = \frac{1}{\sqrt{N_V}}(0,V,0,0,0,1) \tag{351}$$
$$|6\rangle = \frac{1}{\sqrt{N_Z}}(0,Z,0,0,0,1) \tag{352}$$

where the normalizations are $N_X = 1 + X^2$, $N_Y = 1 + Y^2$, $N_V = 1 + V^2$ and $N_Z = 1 + Z^2$ and

$$X = -\frac{1}{2\gamma}\left(W_1 - (6U_2 + \frac{3U_1}{2}) + \sqrt{4\gamma^2 + (W_1 - (6U_2 + \frac{3U_1}{2}))^2}\right) \tag{353}$$
$$Y = -\frac{1}{2\gamma}\left(W_1 - (6U_2 + \frac{3U_1}{2}) - \sqrt{4\gamma^2 + (W_1 - (6U_2 + \frac{3U_1}{2}))^2}\right) \tag{354}$$
$$V = -\frac{1}{2\gamma}\left(W_1 - (6U_2 + \frac{U_1}{2}) + \sqrt{4\gamma^2 + (W_1 - (6U_2 + \frac{U_1}{2}))^2}\right) \tag{355}$$
$$Z = -\frac{1}{2\gamma}\left(W_1 - (6U_2 + \frac{U_1}{2}) - \sqrt{4\gamma^2 + (W_1 - (6U_2 + \frac{U_1}{2}))^2}\right). \tag{356}$$

The energy of the state $|j\rangle$ where $j \in \{1,\dots,6\}$, labelled by E_j is given as

$$E_1 = -W_3 - \frac{J}{2} \tag{357}$$
$$E_2 = -W_3 + \frac{J}{2} \tag{358}$$
$$E_3 = \frac{1}{2}\left(-W_1 - (6U_2 + \frac{3U_1}{2}) - \sqrt{4\gamma^2 + (W_1 - (6U_2 + \frac{3U_1}{2}))^2)}\right) \equiv E_X \tag{359}$$
$$E_4 = \frac{1}{2}\left(-W_1 - (6U_2 + \frac{3U_1}{2}) + \sqrt{4\gamma^2 + (W_1 - (6U_2 + \frac{3U_1}{2}))^2}\right) \equiv E_Y \tag{360}$$
$$E_5 = \frac{1}{2}\left(-W_1 - (6U_2 + \frac{U_1}{2}) - \sqrt{4\gamma^2 + (W_1 - (6U_2 + \frac{U_1}{2}))^2}\right) \equiv E_V \tag{361}$$
$$E_6 = \frac{1}{2}\left(-W_1 - (6U_2 + \frac{U_1}{2}) + \sqrt{4\gamma^2 + (W_1 - (6U_2 + \frac{U_1}{2}))^2}\right) \equiv E_Z \tag{362}$$

We break the $\mathbf{B} = 0$ Hilbert space at Γ into 5 subspaces. These five subspaces are spanned by the states $\{|1\rangle, |2\rangle\}$, $|3\rangle$, $|4\rangle$, $|5\rangle$ and $|6\rangle$ respectively. We can relabel these states by $|\epsilon, l\rangle$, where ϵ labels the subspace and l labels the state spanning the subspace, with $l \in \{1,2\}$ and $l = 1$ for $\epsilon = 1$ and $\epsilon \in \{2,\dots,5\}$, respectively. We promote these states to $\mathbf{B} \neq 0$ as $|\epsilon, l, m\rangle = |\epsilon, l\rangle|m\rangle$, where $|m\rangle$ is the mth LL, i.e. the finite $\mathbf{B}$ $\mathbf{k}\cdot\mathbf{p}$ basis. Now using the SWT procedure introduced in previous section, we eliminate terms in the operator in Eq.(233) which can mix the subspaces, to obtain an effective Hamiltonian for subspace $\epsilon = 1$.

To begin with, we rewrite the operators in Eqs.(233) with respect to the states given in Eqs.(297)-(302) as

$$H = H_0 + \Delta V, \tag{363}$$

where Δ is an artificial parameter that helps us keeping track of the orders in perturbation, to which we compute the effective Hamiltonian and is later set to 1. Along with M, we treat all the terms in operator in Eq.(233) which can

mix the subspaces as perturbation V. The unperturbed part is given as

$$H_0 = \begin{pmatrix} E_1 & 0 & 0 & 0 & 0 & 0 \\ 0 & E_2 & 0 & 0 & 0 & 0 \\ 0 & 0 & E_X & 0 & 0 & 0 \\ 0 & 0 & 0 & E_Y & 0 & 0 \\ 0 & 0 & 0 & 0 & E_V & 0 \\ 0 & 0 & 0 & 0 & 0 & E_Z \end{pmatrix}. \tag{364}$$

The perturbation V is given as

$$V = \begin{pmatrix} 0 & M & i\frac{\sqrt{2}}{\ell}\frac{Xv_\star}{\sqrt{N_X}}a^\dagger & i\frac{\sqrt{2}}{\ell}\frac{Yv_\star}{\sqrt{N_Y}}a^\dagger & 0 & 0 \\ & 0 & 0 & 0 & -i\frac{\sqrt{2}}{\ell}\frac{Vv_\star}{\sqrt{N_V}}a & -i\frac{\sqrt{2}}{\ell}\frac{Zv_\star}{\sqrt{N_Z}}a \\ & & 0 & 0 & i\frac{\sqrt{2}}{\ell}\frac{(X+V)v'_\star}{\sqrt{N_X N_V}}a^\dagger & i\frac{\sqrt{2}}{\ell}\frac{(X+Z)v'_\star}{\sqrt{N_X N_Z}}a^\dagger \\ & & & 0 & i\frac{\sqrt{2}}{\ell}\frac{(Y+V)v'_\star}{\sqrt{N_Y N_V}}a^\dagger & i\frac{\sqrt{2}}{\ell}\frac{(Y+Z)v'_\star}{\sqrt{N_Y N_Z}}a^\dagger \\ & & & & 0 & 0 \\ h.c. & & & & & 0 \end{pmatrix}, \tag{365}$$

Following the SWT procedure in Sec.(Supplementary note 10 A 2), i.e. the steps from Eq.(334)-(342), upto O($\frac{1}{\ell^3}$), the effective Hamiltonian for subspace $\epsilon = 1$ is found to be

$$H_{eff}^{\nu=-1,VP} = \begin{pmatrix} -W_3 + \frac{J}{2} + \hbar\tilde{\omega}_c aa^\dagger & i\frac{A}{\ell^3}a^3 \\ -i\frac{A}{\ell^3}{a^\dagger}^3 & -W_3 - \frac{J}{2} + \hbar\bar{\omega}_c a^\dagger a \end{pmatrix}$$
$$+M\left(1 + \frac{\bar{M}_c}{\ell^2}aa^\dagger + \frac{\tilde{M}_c}{\ell^2}a^\dagger a\right)\sigma_x, \tag{366}$$

where the Pauli matrix acts in the orbital space of $a \in \{4, 3\}$ c-fermions. The cyclotron frequencies and the coefficients accompanying M above are

$$\hbar\bar{\omega}_c = \frac{2v_\star^2}{\ell^2}\left(\frac{X^2}{N_X(E_1 - E_X)} + \frac{Y^2}{N_Y(E_1 - E_Y)}\right) \tag{367}$$
$$\hbar\tilde{\omega}_c = \frac{2v_\star^2}{\ell^2}\left(\frac{V^2}{N_V(E_2 - E_V)} + \frac{Z^2}{N_Z(E_2 - E_Z)}\right) \tag{368}$$
$$\bar{M}_c = v_\star^2\left(\frac{Z^2}{N_Z(E_Z - E_2)(E_1 - E_Z)} + \frac{V^2}{N_V(E_V - E_2)(E_1 - E_V)}\right) \tag{369}$$
$$\tilde{M}_c = v_\star^2\left(\frac{X^2}{N_X(E_1 - E_X)(E_X - E_2)} + \frac{Y^2}{N_Y(E_1 - E_Y)(E_Y - E_2)}\right). \tag{370}$$

The coefficient for $O(\frac{1}{\ell^3})$ term is

$$\begin{aligned} A = {} & 2\sqrt{2}v_\star^2 v'_\star\left(\frac{XV(X+V)}{12N_X N_V}\left(\frac{1}{(E_V - E_X)(E_2 - E_V)} + \frac{1}{(E_V - E_X)(E_X - E_1)} - \frac{2}{(E_X - E_1)(E_2 - E_V)}\right)\right) + \\ & 2\sqrt{2}v_\star^2 v'_\star\left(\frac{YV(Y+V)}{12N_Y N_V}\left(\frac{1}{(E_V - E_Y)(E_2 - E_V)} + \frac{1}{(E_V - E_Y)(E_Y - E_1)} - \frac{2}{(E_Y - E_1)(E_2 - E_V)}\right)\right) + \\ & 2\sqrt{2}v_\star^2 v'_\star\left(\frac{XZ(X+Z)}{12N_X N_Z}\left(\frac{1}{(E_Z - E_X)(E_2 - E_Z)} + \frac{1}{(E_Z - E_X)(E_X - E_1)} - \frac{2}{(E_X - E_1)(E_2 - E_Z)}\right)\right) + \\ & 2\sqrt{2}v_\star^2 v'_\star\left(\frac{YZ(Y+Z)}{12N_Y N_Z}\left(\frac{1}{(E_Z - E_Y)(E_2 - E_Z)} + \frac{1}{(E_Z - E_Y)(E_Y - E_1)} - \frac{2}{(E_Y - E_1)(E_2 - E_Z)}\right)\right) + \\ & -\frac{\sqrt{2}v_\star^2 v'_\star XV(X+V)}{N_X N_V(E_1 - E_V)}\left(\frac{1}{E_X - E_1} - \frac{1}{E_V - E_X} + \frac{E_X - \frac{E_1+E_V}{2}}{(E_X - E_1)(E_V - E_X)}\right) + \end{aligned}$$

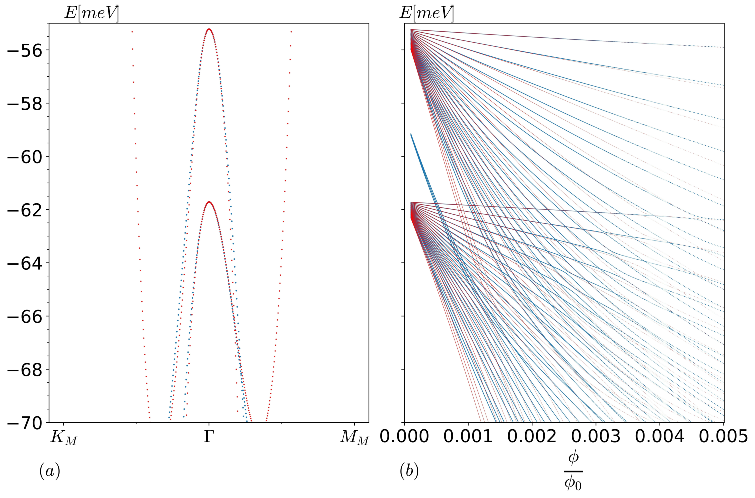

Supplementary Figure 16. The comparison of (b) LL spectrum obtained using the effective Hamiltonian (·) with the exact calculation (·) contrasted with the corresponding comparison at (a) $\mathbf{B}=0$ for VP state at $\nu=-1$ for sector $\mathbf{K}\uparrow$. For the comparison to be tractable we set $m_{max}=25$.

$$
\begin{aligned}
&-\frac{\sqrt{2}v_\star^2 v_\star' YV(Y+V)}{N_Y N_V(E_1-E_V)}\left(\frac{1}{E_Y-E_1}-\frac{1}{E_V-E_Y}+\frac{E_Y-\frac{E_1+E_V}{2}}{(E_Y-E_1)(E_V-E_Y)}\right)+\\
&-\frac{\sqrt{2}v_\star^2 v_\star' XZ(X+Z)}{N_X N_Z(E_1-E_Z)}\left(\frac{1}{E_X-E_1}-\frac{1}{E_Z-E_X}+\frac{E_X-\frac{E_1+E_Z}{2}}{(E_X-E_1)(E_Z-E_X)}\right)+\\
&-\frac{\sqrt{2}v_\star^2 v_\star' YZ(Y+Z)}{N_Y N_Z(E_1-E_Z)}\left(\frac{1}{E_Y-E_1}-\frac{1}{E_Z-E_Y}+\frac{E_Y-\frac{E_1+E_Z}{2}}{(E_Y-E_1)(E_Z-E_Y)}\right)+\\
&\frac{\sqrt{2}v_\star^2 v_\star' XV(X+V)}{N_X N_V(E_X-E_2)}\left(\frac{1}{E_V-E_X}-\frac{1}{E_2-E_V}+\frac{E_V-\frac{E_2+E_X}{2}}{(E_V-E_X)(E_2-E_V)}\right)+\\
&\frac{\sqrt{2}v_\star^2 v_\star' XZ(X+Z)}{N_X N_Z(E_X-E_2)}\left(\frac{1}{E_Z-E_X}-\frac{1}{E_2-E_Z}+\frac{E_Z-\frac{E_2+E_X}{2}}{(E_Z-E_X)(E_2-E_Z)}\right)+\\
&\frac{\sqrt{2}v_\star^2 v_\star' YV(Y+V)}{N_Y N_V(E_Y-E_2)}\left(\frac{1}{E_V-E_Y}-\frac{1}{E_2-E_V}+\frac{E_V-\frac{E_2+E_Y}{2}}{(E_V-E_Y)(E_2-E_V)}\right)+\\
&\frac{\sqrt{2}v_\star^2 v_\star' YZ(Y+Z)}{N_Y N_Z(E_Y-E_2)}\left(\frac{1}{E_Z-E_Y}-\frac{1}{E_2-E_Z}+\frac{E_Z-\frac{E_2+E_Y}{2}}{(E_Z-E_Y)(E_2-E_Z)}\right).
\end{aligned}
\tag{371}
$$

Substituting Eqs.(353)-(362) in the coefficients, we have $\ell^2\hbar\bar{\omega}_c=-692364.83538\text{meV}\text{Å}^2$, $\ell^2\hbar\tilde{\omega}_c=-42241.47578$, $A=-66946640.13364\ \text{meV}\text{Å}^3$, $\bar{M}_c=-15377.73807\text{Å}^2$ and $\tilde{M}_c=-25351.10053\text{Å}^2$. The comparison of the LL spectrum obtained using the above effective Hamiltonian with the one obtained via the exact calculation is shown in Fig.(15). The LL basis used to generate the plot is $(0,|m_1\rangle)^T$, $(|m_2\rangle,|m_2+3\rangle)^T$ and $(|m_3\rangle,0)^T$, where $m_1\in\{0,1,2\}$, $m_2\in\{0,\ldots,m_{max}-4\}$ and $m_3\in\{m_{max}-3,m_{max}-2,m_{max}-1\}$ respectively. We use $(|m_3\rangle,0)^T$ rather than $(|m_3\rangle,|m_3+3\rangle)^T$ in order to avoid three modes emanating out of spurious $\mathbf{B}\to 0$ energy $-(W_3+J/2)$. These three spurious modes are present in the exact calculation, as can be seen in Fig.(15). However, recall that in practice we use $m_{max}=\lceil\frac{q-3}{2}\rceil$, so these three LLs are lost as $\mathbf{B}(q)$ increases(decreases).

K↑ : In this section we discuss the derivation of $\mathbf{B}\neq 0$ effective Hamiltonian for the spin and valley sector $s=\uparrow\mathbf{K}$ for the case of parent VP state at $\nu=-1$. As discussed in Sec.(Supplementary note 6 B), for the above mean-field sector in flat band limit $M=0$, the magnetic subbands within the narrow bands emanate out of the $\mathbf{B}\to 0$ energy $-W_3-\frac{J}{2}$. The remote subbands on the other hand emanate out of the $\mathbf{B}\to 0$ energy $\frac{1}{2}\left(-W_1-(6U_2+3U_1/2)\pm\sqrt{4\gamma^2+(W_1-(6U_2+3U_1/2))^2}\right)$. These energies correspond to the eigenvalues of the flat band $\mathbf{B}=0$ THFM at Γ at $\nu=-1$ for the same sector. Recall that at Γ, it has the form[2]

$\begin{pmatrix} -W_1\sigma_0 & 0 & \gamma\sigma_0 \\ & -(W_3+J/2)\sigma_0 & 0 \\ h.c. & & -(6U_2+3U_1/2)\sigma_0 \end{pmatrix}$, where Pauli matrix σ_0 acts in orbital space. The corresponding eigenvectors can be listed as

$$|1,1\rangle = (0,0,0,1,0,0)^T\,, \tag{372}$$
$$|1,2\rangle = (0,0,1,0,0,0)^T\,, \tag{373}$$
$$|2,1\rangle = \frac{1}{\sqrt{N_X}}\,(0,X,0,0,0,1)^T\,, \tag{374}$$
$$|2,2\rangle = \frac{1}{\sqrt{N_X}}\,(X,0,0,0,1,0)^T\,, \tag{375}$$
$$|3,1\rangle = \frac{1}{\sqrt{N_Y}}\,(0,Y,0,0,0,1)^T\,, \tag{376}$$
$$|3,2\rangle = \frac{1}{\sqrt{N_Y}}\,(Y,0,0,0,1,0)^T\,, \tag{377}$$

where

$$X = -\frac{1}{2\gamma}\left(W_1-(6U_2+3U_1/2)+\sqrt{4\gamma^2+(W_1-(6U_2+3U_1/2))^2}\right), \tag{378}$$
$$Y = -\frac{1}{2\gamma}\left(W_1-(6U_2+3U_1/2)-\sqrt{4\gamma^2+(W_1-(6U_2+3U_1/2))^2}\right), \tag{379}$$

and normalizations $N_X = 1+X^2$, $N_Y = 1+Y^2$. The energy of state ρ, j with $\rho \in \{1,2,3\}$ and $j \in \{1,2\}$, $E_{\rho,j}$, are given as

$$E_{1,j} = -(W_3+\frac{J}{2}) \equiv E, \tag{380}$$
$$E_{2,j} = \frac{1}{2}\left(-W_1-(6U_2+3U_1/2)-\sqrt{4\gamma^2+(W_1-(6U_2+3U_1/2))^2}\right) \equiv E_X, \tag{381}$$
$$E_{3,j} = \frac{1}{2}\left(-W_1-(6U_2+3U_1/2)+\sqrt{4\gamma^2+(W_1-(6U_2+3U_1/2))^2}\right) \equiv E_Y. \tag{382}$$

We break the $\mathbf{B}=0$ Hilbert space at Γ into 3 subspaces. These three subspaces are spanned by the states $|1,j\rangle$, $|2,j\rangle$ and $|3,j\rangle$, respectively. We promote these states to $\mathbf{B}\neq 0$ as $|\rho,j,m\rangle = |\rho,j\rangle|m\rangle$, where $|m\rangle$ is the mth LL, i.e. the finite $\mathbf{B}$ $\mathbf{k}\cdot\mathbf{p}$ basis. Using the SWT procedure introduced in Sec.(Supplementary note 10 A 1), we eliminate all the terms in the operator in Eq.(222) that can mix the subspaces to obtain an effective Hamiltonian for the subspace spanned by states $|1,j,m\rangle$, i.e. the one spanned by $a=\{3,4\}$ c fermions. We first re-write the operator in Eq.(222) with respect to the eigenstates in Eqs.(372)-(377) as

$$H = H_0 + \Delta V, \tag{383}$$

where

$$H_0 = \begin{pmatrix} E\sigma_0 & 0 & 0 \\ 0 & E_X\sigma_0 & 0 \\ 0 & 0 & E_Y\sigma_0 \end{pmatrix}, \tag{384}$$

where Pauli matrix σ_0 acts in the j space for each ρ. Along with M, we treat all the terms in operator in Eq.(222) which can mix the subspaces as perturbation V. Δ is an artificial parameter that helps us keeping track of the orders in perturbation, to which we compute the effective Hamiltonian and is later set to 1. The matrix V can be obtained by replacing X_1, Y_1, N_{X1} and N_{Y1} in Eq.(310) by X, Y, N_X and N_Y respectively. Following the procedure for SWT in Sec.(Supplementary note 10 A 1), i.e. from Eq.(312)-(326) and replacing $|\rho,j,\tau,m\rangle$ by $|\rho,j,m\rangle$ in these steps, the effective Hamiltonian for $\rho=1$ and spin s is found to be

$$H_{VP}^{\tau=1,s=\uparrow,\nu=-1} = \begin{pmatrix} -(W_3+\frac{J}{2})+\hbar\omega_c aa^\dagger & i\frac{A}{\ell^3}a^3 \\ -i\frac{A}{\ell^3}{a^\dagger}^3 & -(W_3+\frac{J}{2})+\hbar\omega_c a^\dagger a \end{pmatrix} + M\left(1+\frac{M_c}{\ell^2}(aa^\dagger+a^\dagger a)\right)\sigma_x, \tag{385}$$

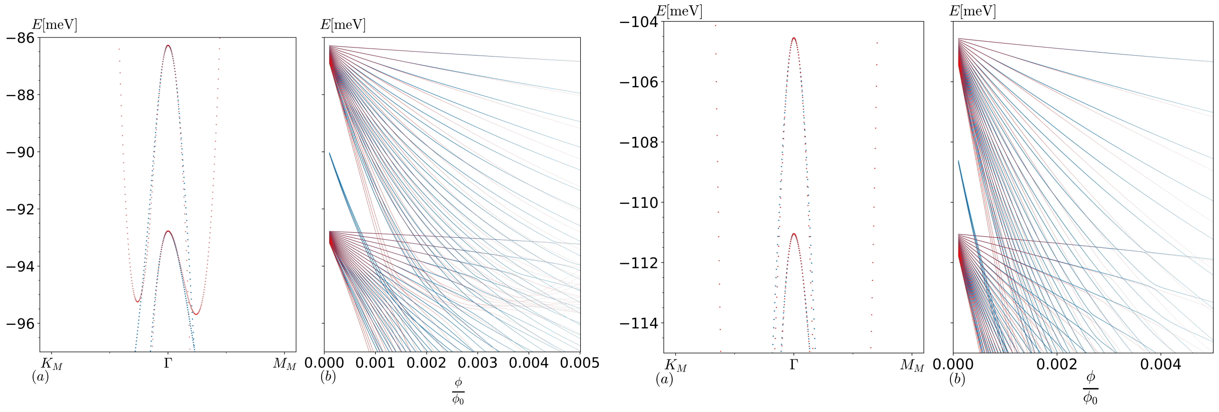

Supplementary Figure 17. (i)**Left Panel** - a,b : The comparison of (b) LL spectrum obtained using the effective Hamiltonian (•) with the exact calculation (•) contrasted with the corresponding comparison at (a) $\mathbf{B} = 0$ for valley $\mathbf{K}$ spin $\downarrow$ at $\nu = -2$. (ii) **Right Panel** - a,b : The comparison of (b) LL spectrum obtained using the effective Hamiltonian (•) with the exact calculation (•) contrasted with the corresponding comparison at (a) $\mathbf{B} = 0$ for valley $\mathbf{K}$ spin $\uparrow$ at $\nu = -2$. For the comparison to be tractable we set an upper-cutoff of 25 on the LL index.

where the Pauli matrix acts in the orbital space of $a \in \{4, 3\}$ c-fermions. The cyclotron frequency ω_c and the other coefficients above are

$$\hbar\omega_c = \frac{2v_\star^2}{\ell^2}\left(\frac{X^2}{N_X(E - E_X)} + \frac{Y^2}{N_Y(E - E_Y)}\right), \tag{386}$$

$$A = 4\sqrt{2}v_\star^2 v_\star'\left(\frac{XY(X+Y)}{N_X N_Y(E - E_X)(E - E_Y)} + \frac{X^3}{N_X^2(E - E_X)^2} + \frac{Y^3}{N_Y^2(E - E_Y)^2}\right), \tag{387}$$

$$M_c = -v_\star^2\left(\frac{X^2}{N_X(E - E_X)^2} + \frac{Y^2}{N_Y(E - E_Y)^2}\right). \tag{388}$$

Substituting Eqs.(378)-(379) in the coefficients, we have $\ell^2\hbar\omega_c = -692364.83538\text{meV}\text{Å}^2$, $A = -61125292.83865\text{meV}\text{Å}^3$ and $M_c = -13387.26641\text{Å}^2$. For the U(4) symmetric THFM (i.e. $M = 0$), in the $\mathbf{B} \to 0$ limit, we can the drop off-diagonal $\mathrm{O}(\ell^{-3})$ terms in in the above effective Hamiltonian. Then apart from the mode $(0, |0\rangle)^T$, all other LLs come in degenerate pair of two. This degeneracy at $\mathbf{B} \to 0$ limit gets split as we tune back M. The comparison of the LL spectrum obtained using the above effective Hamiltonian with the one obtained via the exact calculation is shown in Fig.(16). The LL basis used to generate the plot is $(0, |m_1\rangle)^T$, $(|m_2\rangle, |m_2 + 3\rangle)^T$ and $(|m_3\rangle, 0)^T$, where $m_1 \in \{0, 1, 2\}$, $m_2 \in \{0, \ldots, m_{max} - 4\}$ and $m_3 \in \{m_{max} - 3, m_{max} - 2, m_{max} - 1\}$ respectively. We use $(|m_3\rangle, 0)^T$ rather than $(|m_3\rangle, |m_3 + 3\rangle)^T$ in order to avoid three modes emanating out of spurious $\mathbf{B} \to 0$ energy $-(W_3 + J/2)$. These three spurious modes are present in the exact calculation, as can be seen in Fig.(16). However, recall that in practice we use $m_{max} = \lceil \frac{q-3}{2} \rceil$, so these three LLs are lost as $\mathbf{B}(q)$ increases(decreases).

C. $\nu = -2$

1. *Parent Valley Polarized State*

In this section we discuss the derivation of the case of $\mathbf{B} \neq 0$ effective Hamiltonian for the spin and valley sector $s =\uparrow, \downarrow$, $\mathbf{K}$ for the case of parent VP state at $\nu = -2$. As discussed in Sec.(Supplementary note 6 D), for the above mean-field sector in flat band limit $M = 0$, the magnetic subbands within the narrow bands emanate out of the $\mathbf{B} \to 0$ energy $-2W_3 - \zeta_s \frac{J}{2}$, where $\zeta_s = 1, -1$ for $s =\uparrow, \downarrow$ respectively. The remote subbands on the other hand emanate out of the $\mathbf{B} \to 0$ energy $\frac{1}{2}\left(-2W_1 - (12U_2 + \frac{4+\zeta_s}{2}U_1) \pm \sqrt{4\gamma^2 + (2W_1 - (12U_2 + \frac{4+\zeta_s}{2}U_1))^2}\right)$. These energies correspond to the eigenvalues of the flat band $\mathbf{B} = 0$ THFM at Γ at $\nu = -2$ for the same mean-field sector. Recall that at Γ, it has the form[2] $\begin{pmatrix} -2W_1\sigma_0 & 0 & \gamma\sigma_0 \\ & -(2W_3 + \zeta_s J/2)\sigma_0 & 0 \\ h.c. & & -(12U_2 + \frac{4+\zeta_s}{2}U_1)\sigma_0 \end{pmatrix}$, where Pauli matrix σ_0 acts in orbital space.

The corresponding eigenvectors for given spin s can be listed as

$$|1,1,s\rangle = (0,0,0,1,0,0)^T\,, \tag{389}$$
$$|1,2,s\rangle = (0,0,1,0,0,0)^T\,, \tag{390}$$
$$|2,1,s\rangle = \frac{1}{\sqrt{N_{Xs}}}(0,X_s,0,0,0,1)^T\,, \tag{391}$$
$$|2,2,s\rangle = \frac{1}{\sqrt{N_{Xs}}}(X_s,0,0,0,1,0)^T\,, \tag{392}$$
$$|3,1,s\rangle = \frac{1}{\sqrt{N_{Ys}}}(0,Y_s,0,0,0,1)^T\,, \tag{393}$$
$$|3,2,s\rangle = \frac{1}{\sqrt{N_{Ys}}}(Y_s,0,0,0,1,0)^T\,, \tag{394}$$

where

$$X_s = -\frac{1}{2\gamma}\left(2W_1-(12U_2+\frac{4+\zeta_s}{2}U_1)+\sqrt{4\gamma^2+(2W_1-(12U_2+\frac{4+\zeta_s}{2}U_1))^2}\right), \tag{395}$$
$$Y_s = -\frac{1}{2\gamma}\left(2W_1-(12U_2+\frac{4+\zeta_s}{2}U_1)-\sqrt{4\gamma^2+(2W_1-(12U_2+\frac{4+\zeta_s}{2}U_1))^2}\right), \tag{396}$$

and normalizations $N_{Xs}=1+X_s^2$, $N_{Ys}=1+Y_s^2$. The energy of state ρ,j,s with $\rho\in\{1,2,3\}$ and $j\in\{1,2\}$, $E_{\rho,j,s}$, are given as

$$E_{1,j,s} = -(2W_3+\zeta_s\frac{J}{2})\equiv E_s, \tag{397}$$
$$E_{2,j,s} = \frac{1}{2}\left(-2W_1-(12U_2+\frac{4+\zeta_s}{2}U_1)-\sqrt{4\gamma^2+(2W_1-(12U_2+\frac{4+\zeta_s}{2}U_1))^2}\right)\equiv E_{Xs}, \tag{398}$$
$$E_{3,j,s} = \frac{1}{2}\left(-2W_1-(12U_2+\frac{4+\zeta_s}{2}U_1)+\sqrt{4\gamma^2+(2W_1-(12U_2+\frac{4+\zeta_s}{2}U_1))^2}\right)\equiv E_{Ys}. \tag{399}$$

We break the $\mathbf{B}=0$ Hilbert space at Γ into 3 subspaces for each s. These three subspaces are spanned by the states $|1,j,s\rangle$, $|2,j,s\rangle$ and $|3,j,s\rangle$, respectively. We promote these states to $\mathbf{B}\neq 0$ as $|\rho,j,s,m\rangle = |\rho,j,s\rangle|m\rangle$, where $|m\rangle$ is the mth LL, i.e. the finite $\mathbf{B}$ $\mathbf{k}\cdot\mathbf{p}$ basis. Using the SWT procedure introduced in Sec.(Supplementary note 10 A 1), we eliminate all the terms in the operator in Eq.(244) that can mix the subspaces to obtain an effective Hamiltonian for the subspace spanned by states $|1,j,s,m\rangle$, i.e. the one spanned by $a=\{3,4\}$ c fermions.

We first re-write the operator in Eq.(244) with respect to the eigenstates in Eqs.(389)-(394) as

$$H = H_0+\Delta V, \tag{400}$$

where

$$H_0 = \begin{pmatrix} E_s\sigma_0 & 0 & 0\\ 0 & E_{Xs}\sigma_0 & 0\\ 0 & 0 & E_{Ys}\sigma_0\end{pmatrix}, \tag{401}$$

where Pauli matrix σ_0 acts in the j space for each ρ. Along with M, we treat all the terms in operator in Eq.(244) which can mix the subspaces as perturbation V. Δ is an artificial parameter that helps us keeping track of the orders in perturbation, to which we compute the effective Hamiltonian and is later set to 1. The matrix V can be obtained by replacing X_1, Y_1, N_{X1} and N_{Y1} in Eq.(310) by X_s, Y_s, N_{Xs} and N_{Ys} respectively. Following the procedure for SWT in Sec.(Supplementary note 10 A 1), i.e. from Eq.(312)-(326) and replacing $|\rho,j,\tau,m\rangle$ by $|\rho,j,s,m\rangle$ in these steps, the effective Hamiltonian for $\rho=1$ and spin s is found to be

$$H_{VP}^{\tau=1,s=\uparrow,\downarrow,\nu=-2} = \begin{pmatrix} -(2W_3+\zeta_s\frac{J}{2})+\hbar\omega_c^{(s)}aa^\dagger & i\frac{A^{(s)}}{\ell^3}a^3\\ -i\frac{A^{(s)}}{\ell^3}a^{\dagger 3} & -(2W_3+\zeta_s\frac{J}{2})+\hbar\omega_c^{(s)}a^\dagger a\end{pmatrix} + M\left(1+\frac{M_c^{(s)}}{\ell^2}(aa^\dagger+a^\dagger a)\right)\sigma_z, \tag{402}$$

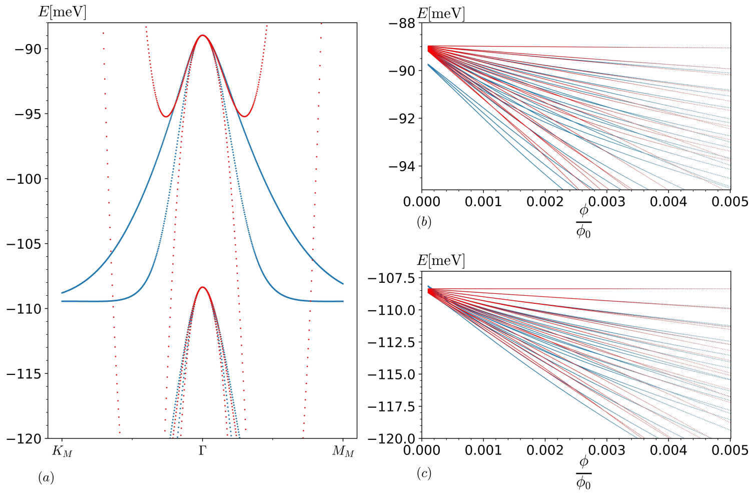

Supplementary Figure 18. The comparison of (b,c) LL spectrum obtained using the effective Hamiltonian (·) with the exact calculation (·) contrasted with the corresponding comparison at (a) $\mathbf{B} = 0$ for spin sector ↑ for the KIVC state at $\nu = -2$. For the comparison to be tractable we set an upper-cutoff of 10 on the LL index.

where the Pauli matrix acts in the orbital space of $a \in \{4, 3\}$ c-fermions. The cyclotron frequency $\omega_c^{(s)}$ and the other coefficients above are

$$\hbar\omega_c^{(s)} = \frac{2v_\star^2}{\ell^2}\left(\frac{X_s^2}{N_{Xs}(E_s - E_{Xs})} + \frac{Y_s^2}{N_{Ys}(E_s - E_{Ys})}\right), \tag{403}$$

$$A^{(s)} = 4\sqrt{2}v_\star^2 v_\star' \left(\frac{X_s Y_s (X_s + Y_s)}{N_{Xs}N_{Ys}(E_s - E_{Xs})(E_s - E_{Ys})} + \frac{X_s^3}{N_{Xs}^2(E_s - E_{Xs})^2} + \frac{Y_s^3}{N_{Ys}^2(E_s - E_{Ys})^2}\right), \tag{404}$$

$$M_c^{(s)} = -v_\star^2\left(\frac{X_s^2}{N_{Xs}(E_s - E_{Xs})^2} + \frac{Y_s^2}{N_{Ys}(E_s - E_{Ys})^2}\right). \tag{405}$$

Substituting Eqs.(395)-(399) in the coefficients, we have $\ell^2\hbar\omega_c^{(\uparrow)} = -831301.40524$meVÅ^2, $\ell^2\hbar\omega_c^{(\downarrow)} = -514097.23133$ meVÅ^2, $A^{(\uparrow)} = -57864769.72509$meVÅ^3, $A^{(\downarrow)} = -59278877.34125$ meVÅ^3, $M_c^{(\uparrow)} = -13211.61944$Å^2 and $M_c^{(\downarrow)} = -15952.11343$Å^2. For the U(4) symmetric THFM (i.e. $M = 0$), in the $\mathbf{B} \to 0$ limit, we can the drop off-diagonal $O(\ell^{-3})$ terms in in the above effective Hamiltonian. Then apart from the mode $(0, |0\rangle)^T$, all other LLs come in degenerate pair of two. This degeneracy at $\mathbf{B} \to 0$ limit gets split as we tune back M. The comparison of the LL spectrum obtained using the above effective Hamiltonian with the one obtained via the exact calculation is shown in Fig.(17). The LL basis used to generate the plot is $(0, |m_1\rangle)^T$, $(|m_2\rangle, |m_2 + 3\rangle)^T$ and $(|m_3\rangle, 0)^T$, where $m_1 \in \{0, 1, 2\}$, $m_2 \in \{0, \ldots, m_{max} - 4\}$ and $m_3 \in \{m_{max} - 3, m_{max} - 2, m_{max} - 1\}$ respectively. We use $(|m_3\rangle, 0)^T$ rather than $(|m_3\rangle, |m_3 + 3\rangle)^T$ in order to avoid three modes emanating out of spurious $\mathbf{B} \to 0$ energy $-(2W_3 + \zeta_s J/2)$, for each s. These three spurious modes are present in the exact calculation, as can be seen in Fig.(17). However, recall that in practice we use $m_{max} = \lceil \frac{q-3}{2} \rceil$, so these three LLs are lost as $\mathbf{B}(q)$ increases(decreases).

2. *Parent Kramers Intervalley Coherent State*

In this section, we discuss the derivation of $\mathbf{B} \neq 0$ effective Hamiltonian for the spin sector $s =\uparrow$ for the case of parent KIVC state at $\nu = -2$. The exact $\mathbf{B} = 0$ THFM for the spin sector $s =\uparrow$ at $\nu = -2$ for the parent KIVC state is presented in Eq.(295). The coupled modes of c and f fermions at $\mathbf{B} \neq 0$ are described by the operator in Eq.(296). In the flat band limit at Γ, the $\mathbf{B} = 0$ THFM has the form $\begin{pmatrix} H^{\tau=1,\nu=-2} & 0 \\ 0 & H^{\tau=-1,\nu=-2} \end{pmatrix}$, where $\begin{pmatrix} -2W_1\sigma_0 & 0 & \gamma\sigma_0 \\ & -(2W_3 + \tau J/2)\sigma_0 & 0 \\ h.c. & & -(12U_2 + \frac{4+\tau}{2}U_1)\sigma_0 \end{pmatrix}$, where the Pauli matrix σ_0 acts in the orbital space. The

corresponding eigenstates, labelled by $|\rho, j, \tau\rangle$ with $\rho \in \{1,2,3\}$ and $j \in \{1,2\}$ are given as

$$|1,1,\tau=+1\rangle = (0,0,0,1,0,0,0_{6\times1})^T\,; \qquad |1,1,\tau=-1\rangle = (0_{6\times1},0,0,0,1,0,0)^T \tag{406}$$
$$|1,2,\tau=+1\rangle = (0,0,1,0,0,0,0_{6\times1})^T\,; \qquad |1,2,\tau=-1\rangle = (0_{6\times1},0,0,1,0,0,0)^T \tag{407}$$
$$|2,1,\tau=+1\rangle = \frac{1}{\sqrt{N_{X1}}}(0,X_1,0,0,0,1,0_{6\times1})^T\,; \quad |2,1,\tau=-1\rangle = \frac{1}{\sqrt{N_{X-1}}}(0_{6\times1},0,X_{-1},0,0,0,1)^T \tag{408}$$
$$|2,2,\tau=+1\rangle = \frac{1}{\sqrt{N_{X1}}}(X_1,0,0,0,1,0,0_{6\times1})^T\,; \quad |2,2,\tau=-1\rangle = \frac{1}{\sqrt{N_{X-1}}}(0_{6\times1},X_{-1},0,0,0,1,0)^T \tag{409}$$
$$|3,1,\tau=+1\rangle = \frac{1}{\sqrt{N_{Y1}}}(0,Y_1,0,0,0,1,0_{6\times1})^T\,; \quad |3,1,\tau=-1\rangle = \frac{1}{\sqrt{N_{Y-1}}}(0_{6\times1},0,Y_{-1},0,0,0,1)^T \tag{410}$$
$$|3,2,\tau=+1\rangle = \frac{1}{\sqrt{N_{Y1}}}(Y_1,0,0,0,1,0,0_{6\times1})^T\,; \quad |3,2,\tau=-1\rangle = \frac{1}{\sqrt{N_{Y-1}}}(0_{6\times1},Y_{-1},0,0,0,1,0)^T \tag{411}$$

where the normalizations are $N_{X\tau} = 1 + X_\tau^2$, $N_{Ys} = 1 + Y_\tau^2$ and

$$X_\tau = -\frac{1}{2\gamma}\left(2W_1 - (12U_2 + \frac{4+\tau}{2}U_1) + \sqrt{4\gamma^2 + (2W_1 - (12U_2 + \frac{4+\tau}{2}U_1))^2}\right), \tag{412}$$
$$Y_\tau = -\frac{1}{2\gamma}\left(2W_1 - (12U_2 + \frac{4+\tau}{2}U_1) - \sqrt{4\gamma^2 + (2W_1 - (12U_2 + \frac{4+\tau}{2}U_1))^2}\right), \tag{413}$$

The energy of state ρ, j, τ with $\rho \in \{1,2,3\}$ and $j \in \{1,2\}$, $E_{\rho,j,\tau}$, are given as

$$E_{1,j,\tau} = -(2W_3 + \tau\frac{J}{2}) \equiv E_\tau \tag{414}$$
$$E_{2,j,\tau} = \frac{1}{2}\left(-2W_1 - (12U_2 + \frac{4+\tau}{2}U_1) - \sqrt{4\gamma^2 + (2W_1 - (12U_2 + \frac{4+\tau}{2}U_1))^2}\right) \equiv E_{X\tau} \tag{415}$$
$$E_{3,j,\tau} = \frac{1}{2}\left(-2W_1 - (12U_2 + \frac{4+\tau}{2}U_1) + \sqrt{4\gamma^2 + (2W_1 - (12U_2 + \frac{4+\tau}{2}U_1))^2}\right) \equiv E_{Y\tau} \tag{416}$$

We break the $\mathbf{B} = 0$ Hilbert space at Γ into 5 subspaces. The subspace of interest is the one spanned by states $|1, j, \tau\rangle$ with $j \in \{1,2\}$ and $\tau = \pm1$, i.e. the one spanned by $a = \{3,4\}$ c fermions at each valley. The remaining four subspaces are spanned by $|2, j, +1\rangle$, $|2, j, -1\rangle$, $|3, j, +1\rangle$ and $|3, j, -1\rangle$, respectively with $j \in \{1,2\}$. These states and their energies can now be relabelled by $|\epsilon, l\rangle$ and $E_{\epsilon l}$, respectively. Here $\epsilon \in \{1,\dots,5\}$ labels the 5 subspaces, $l \in \{1,\dots,4\}$ for $\epsilon = 1$ and $l \in \{1,2\}$ for $\epsilon = \{3,4,5\}$ labels the states spanning the subspace ϵ. We promote these states to $\mathbf{B} \neq 0$ as $|\epsilon, l, m\rangle = |\epsilon, l\rangle|m\rangle$, where $|m\rangle$ is the mth LL, i.e. the finite $\mathbf{B}$ $\mathbf{k}\cdot\mathbf{p}$ basis.

To begin with, we re-write the operator in Eq.(296) with respect to the above $\mathbf{B} = 0$ eigenstates at Γ as

$$H = H_0 + \Delta V, \tag{417}$$

where $H_0 = \begin{pmatrix} H_0{}_{KIVC}^{\tau=+1} & \\ & H_0{}_{KIVC}^{\tau=-1} \end{pmatrix}$ and $V = \begin{pmatrix} \bar{V}_{KIVC}^{\tau=+1} & V_M \\ h.c. & \bar{V}_{KIVC}^{\tau=-1} \end{pmatrix}$. $H_0{}_{KIVC}^{\tau}$ can be obtained by replacing ζ_s by τ in Eq.(401). The intra-valley perturbation $\bar{V}_{KIVC}^{\tau=+1}$ can be obtained by setting $M = 0$ in Eq.(310) and $\bar{V}_{KIVC}^{\tau=-1}$ can be obtained by replacing $X_1, Y_1 \to X_{-1}, Y_{-1}$, $N_{X1}, N_{Y1} \to N_{X-1}, N_{Y-1}$ and $a \leftrightarrow a^\dagger$ in $V^{\tau=+1}$. However note that the values of Y_τ and Y_τ are changed for $\nu = -2$ and given in Eq.(412) and Eq.(413) respectively. The inter-valley perturbation V_M is given in Eq.(333). Following the SWT procedure in Sec.(Supplementary note 10 A 2), i.e. the steps from Eq.(334)-(342), upto $O(\frac{1}{\ell^3})$, the effective Hamiltonian for subspace $\epsilon = 1$ is found to be

$$H^{eff} = \begin{pmatrix} H_{\text{KIVC}}^{\tau=+1,\nu=-2} & H_M^{\nu=-2} \\ h.c. & H_{\text{KIVC}}^{\tau=-1,\nu=-2} \end{pmatrix}, \tag{418}$$

where

$$H_{\text{KIVC}}^{\tau=+1,\nu=-2} = \begin{pmatrix} -(2W_3 + \frac{J}{2}) + \hbar\omega_c^{(1)}aa^\dagger & i\frac{A^{(1)}}{\ell^3}a^3 \\ -i\frac{A^{(1)}}{\ell^3}a^{\dagger 3} & -(2W_3 + \frac{J}{2}) + \hbar\omega_c^{(1)}a^\dagger a \end{pmatrix}. \tag{419}$$

$H_{\rm KIVC}^{\tau=-1,\nu=-2}$ can be obtained by replacing $a \leftrightarrow a^\dagger$ and $\omega_c^{(1)}, A^{(1)}, \frac{J}{2} \to \omega_c^{(-1)}, A^{(-1)}, -\frac{J}{2}$ in Eq.(419). The cyclotron frequency $\omega_c^{(\tau)}$ and coefficient A^τ are

$$\hbar\omega_c^{(\tau)} = \frac{2v_\star^2}{\ell^2}\left(\frac{X_\tau^2}{N_{X\tau}(E_\tau - E_{X\tau})} + \frac{Y_\tau^2}{N_{Y\tau}(E_\tau - E_{Y\tau})}\right), \tag{420}$$

$$A^{(\tau)} = 4\sqrt{2}v_\star^2 v_\star' \left(\frac{X_\tau Y_\tau (X_\tau + Y_\tau)}{N_{X\tau}N_{Y\tau}(E_\tau - E_{X\tau})(E_\tau - E_{Y\tau})} + \frac{X_\tau^3}{N_{X\tau}^2(E_\tau - E_{X\tau})^2} + \frac{Y_\tau^3}{N_{Y\tau}^2(E_\tau - E_{Y\tau})^2}\right),. \tag{421}$$

The matrix H_M couples both the valleys and is given as

$$H_M = M\left(1 + \frac{M_{c1}}{\ell^2}a^\dagger a + \frac{M_{c2}}{\ell^2}aa^\dagger\right)\sigma_z \tag{422}$$

where the Pauli matrix acts in the orbital space of $a \in \{4,3\}$ c-fermions and

$$M_{c1} = v_\star^2\left(\frac{X_{-1}^2}{N_{X-1}(E_1 - E_{X-1})(E_{X-1} - E_{-1})} + \frac{Y_{-1}^2}{N_{Y-1}(E_1 - E_{Y-1})(E_{Y-1} - E_{-1})}\right), \tag{423}$$

$$M_{c2} = v_\star^2\left(\frac{X_1^2}{N_{X1}(E_1 - E_{X1})(E_{X1} - E_{-1})} + \frac{Y_1^2}{N_{Y1}(E_1 - E_{Y1})(E_{Y1} - E_{-1})}\right). \tag{424}$$

Substituting Eqs.(412)-(399) in the coefficients, we have $\ell^2\hbar\omega_c^{(1)} = -831301.40524$meVÅ^2, $\ell^2\hbar\omega_c^{(-1)} = -514097.23133$ meVÅ^2, $A^{(1)} = -57864769.72509$meVÅ^3, $A^{(-1)} = -59278877.34125$ meVÅ^3, $M_{c1} = -12902.68825$Å^2 and $M_{c2} = -25550.93059$Å^2. The comparison of the LL spectrum obtained using the above effective Hamiltonian with the one obtained via the exact calculation is shown in Fig.(18). The LL basis used to generate the plot is $(0, |m_1\rangle, |m_1\rangle, 0)^T$, $(|m_2\rangle, |m_2+3\rangle, |m_2+3\rangle, |m_2\rangle)^T$ and $(|m_3\rangle, 0, 0, |m_3\rangle)^T$, where $m_1 \in \{0,1,2\}$, $m_2 \in \{0,\ldots,m_{max}-4\}$ and $m_3 \in \{m_{max}-3, m_{max}-2, m_{max}-1\}$ respectively. We use $(|m_3\rangle, 0, 0, |m_3\rangle)^T$ rather than $(|m_3\rangle, |m_3+3\rangle, |m_3+3\rangle, |m_3\rangle)^T$ in order to avoid three modes emanating out of spurious $\mathbf{B} \to 0$ energy $-2W_3 \pm J/2$. These six spurious modes are present in the exact calculation, as can be seen in Fig.(18b) and (18c). However, recall that in practice we use $m_{max} = \lceil \frac{q-3}{2} \rceil$, so these three LLs are lost as $\mathbf{B}(q)$ increases(decreases).

Supplementary note 11. REVIEW OF MAGNETIC BLOCH'S THEOREM AT 2π FLUX

In the following two appendices we detail the gauge-invariant technique of calculating the strong-coupling spectrum of TBG in flux. We first review our technique of calculating the band structures and topology of twisted bilayer graphene with 2π flux per unit cell, as derived in [7, 8], with the eventual goal of extending the 2π formalism to rational flux $\phi = 2\pi p/q$ (note the change in notation : $\phi/2\pi$ is the magnetic flux per moiré unit cell). Compared to other techniques, our method explicitly defines gauge-invariant *momentum eigenstates* $|\mathbf{k}, m\rangle$ that are simultaneous eigenkets of magnetic translation operators with the same formal properties as Bloch states. By keeping the full momentum quantum numbers and manifestly preserving the lattice symmetries, we are able to leverage the machinery of band topology, such as Wilson loops and the quantum metric tensor as will be explored in future work, in Hofstadter systems at rational flux.

To add nonzero flux to the BM model, we perform canonical substitution: $-i\partial \to \boldsymbol{\pi}$, with $\boldsymbol{\pi}$ the canonical momentum yielding the operators

$$\pi_\mu = -i\partial_\mu - eA_\mu, \quad Q_\mu = \pi_\mu - eB\epsilon_{\mu\nu}x_\nu. \tag{425}$$

where $\mathbf{A}$ is the vector potential $\boldsymbol{\nabla} \times \mathbf{A} = B\hat{z}$, and Q_μ is the guiding center operator obeying $[\pi_\mu, Q_\nu] = 0$. We define the Landau level operators a, b as

$$a = \frac{\pi_x + i\pi_y}{\sqrt{2B}}, \quad a^\dagger = \frac{\pi_x - i\pi_y}{\sqrt{2B}}, \quad [a, a^\dagger] = 1, \tag{426}$$

$$b = \frac{(\mathbf{L}_1 - i\mathbf{L}_2)\cdot\mathbf{Q}}{\sqrt{2\phi}}, \quad b^\dagger = \frac{(\mathbf{L}_1 + i\mathbf{L}_2)\cdot\mathbf{Q}}{\sqrt{2\phi}}, \quad [b, b^\dagger] = 1\ . \tag{427}$$

In magnetic flux, the translation operators $T_\mathbf{R}$ which commute with the Hamiltonian are

$$\hat{t}_\mathbf{R} = e^{i\mathbf{R}\cdot\mathbf{Q}} \tag{428}$$

where $\mathbf{R}$ is a lattice vector. Because at 2π flux the magnetic translation operators $T_{\mathbf{L}_1}, T_{\mathbf{L}_2}$ commute with each other and the Hamiltonian H_{BM}, one can label the eigenstates of H_{BM} by the momentum carried by the operators $T_{\mathbf{L}_1}, T_{\mathbf{L}_2}$. A gauge-invariant definition of the momentum eigenstates was given in [7] as

$$|\mathbf{k}, m\rangle = \frac{1}{\sqrt{\mathcal{N}(k_1, k_2)}} \sum_{\mathbf{R}} e^{-2\pi i \mathbf{k}\cdot\mathbf{R}} T_{\mathbf{L}_1}^{R_1} T_{\mathbf{L}_2}^{R_2} |m\rangle\,, \qquad \langle \mathbf{k}, m|\mathbf{k}', n\rangle = \delta(\mathbf{k}-\mathbf{k}')\delta_{mn}\;. \tag{429}$$

Here momentum is $\mathbf{k} = k_1\mathbf{g}_1 + k_2\mathbf{g}_2$, $k_i = \mathbf{k}\cdot\mathbf{L}_i \in [0,1)$, and $\mathbf{R} = R_1\mathbf{L}_1 + R_2\mathbf{L}_2$ is summed over all integers R_1, R_2. The ket $|m\rangle$ is the mth Landau level

$$|m\rangle = \frac{1}{\sqrt{m!}} a^{\dagger m} |0\rangle \tag{430}$$

and $\mathcal{N}(k_1, k_2)$ is the normalization factor, worked out to be [7]

$$\mathcal{N}(k_1,k_2) = \vartheta\left(k_1, k_2 \middle| \varPhi\right) = \sqrt{2} e^{-\pi(k_1^2+k_2^2-2ik_1k_2)} \theta\left(ik_1 + k_2 \middle| i\right) \theta\left(k_1 + ik_2 \middle| i\right). \tag{431}$$

Here ϑ denotes the Siegel theta function and $\varPhi$ the Riemann matrix given by

$$\vartheta(\mathbf{k}|M) = \sum_{\mathbf{R}} e^{2\pi^2 \mathbf{k}\cdot M\cdot\mathbf{k} + 2\pi i \mathbf{k}\cdot\mathbf{R}}, \qquad \varPhi = \frac{i}{2}\begin{bmatrix} 1 & i \\ i & 1 \end{bmatrix}. \tag{432}$$

In Eq.(431), we have simplified the Siegel theta into a product of Jacobi theta functions θ. There is a zero in the normalization at $\mathbf{k} = (\pi, \pi)$ which is protected by the Chern number of the Landau level states $|\mathbf{k}, m\rangle$.

Within this basis, one may calculate the matrix elements at momentum $\mathbf{k}$. The matrix elements read [7]

$$\langle \mathbf{k}, m|a^\dagger a|\mathbf{k}', n\rangle = \delta(\mathbf{k}-\mathbf{k}') n \delta_{mn} \tag{433}$$

$$\langle \mathbf{k}, m|e^{-i\mathbf{q}\cdot\mathbf{r}}|\mathbf{k}', n\rangle = \delta(\mathbf{k}-\mathbf{k}'-\mathbf{q}) e^{i\xi_{\mathbf{q}}(\mathbf{k})} \mathcal{H}^{\mathbf{q}}_{mn}, \tag{434}$$

where the form factor matrix and phases are defined as

$$e^{i\xi_{\mathbf{q}}(\mathbf{k})} = \frac{e^{-\frac{q\bar{q}}{4\phi}} \vartheta\left(\frac{(k_1 - q/2, k_2 + iq/2)}{2\pi}\middle|\varPhi\right)}{\sqrt{\vartheta\left(\frac{(k_1,k_2)}{2\pi}\middle|\varPhi\right)\vartheta\left(\frac{(k_1-q_1,k_2-q_2)}{2\pi}\middle|\varPhi\right)}}, \quad q = q_1 + iq_2, \quad q_j = \mathbf{q}\cdot\mathbf{L}_j \tag{435}$$

$$\mathcal{H}^{\mathbf{q}}_{mn} = \langle m| e^{i\frac{\gamma_q a + \bar{\gamma}_q a^\dagger}{\sqrt{2\phi}}} |n\rangle, \quad \gamma_q = \epsilon_{ij} q_i \bar{z}_j, \quad \bar{z}_j = \frac{(\hat{x} - i\hat{y})\cdot\mathbf{L}_j}{\sqrt{\Omega}}, \tag{436}$$

with $\Omega = |\mathbf{a}_1 \times \mathbf{a}_2|$ the area of the unit cell. These matrix elements have been worked out in detail in [7], and the momentum Landau levels form a complete orthonormal basis. To evaluate the band structure of the BM model in flux, one simply evaluates the matrix elements of the Hamiltonian with respect to this basis at any momentum. Being able to block diagonalize the Hamiltonian by momentum allows for efficient calculations as well as access to the Wilson loop and quantum geometric tensor.

Supplementary note 12. RATIONAL FLUX

To solve the Bistritzer MacDonald model (or other continuum models) at rational flux $\phi = 2\pi p/q$, we will create new momentum eigenstates that reflect the projective representation of the translation group in flux. At flux $\phi = 2\pi p/q$, we generalize Eq.(427) to the following raising and lowering operators

$$a = \frac{\pi_x + i\pi_y}{\sqrt{2B}}, \quad a^\dagger = \frac{\pi_x - i\pi_y}{\sqrt{2B}} \tag{437}$$

$$b = \frac{(\mathbf{L}_1/p - iq\mathbf{L}_2)\cdot\mathbf{Q}}{\sqrt{4\pi}}, \quad b^\dagger = \frac{(\mathbf{L}_1/p + iq\mathbf{L}_2)\cdot\mathbf{Q}}{\sqrt{4\pi}} \tag{438}$$

The $a, a^\dagger$ operators are unchanged and obey $[a, a^\dagger] = 1$, and we verify that $[b, b^\dagger] = 1$ holds:

$$[b, b^\dagger] = 2i\frac{1}{4\pi}\frac{q}{p}[\mathbf{L}_1 \cdot \mathbf{Q}, \mathbf{L}_2 \cdot \mathbf{Q}] = eB\Omega\frac{1}{2\pi}\frac{q}{p} = 1 \tag{439}$$

since $eB\Omega = \phi$. The translation operators $T_{\mathbf{R}} = e^{i\mathbf{R}\cdot\mathbf{Q}}$ now obey

$$\hat{t}_{\mathbf{L}_1}\hat{t}_{\mathbf{L}_2} = e^{-[\mathbf{L}_1\cdot\mathbf{Q},\mathbf{L}_2\cdot\mathbf{Q}]}\hat{t}_{\mathbf{L}_2}\hat{t}_{\mathbf{L}_1} = e^{i\phi}\hat{t}_{\mathbf{L}_2}\hat{t}_{\mathbf{L}_1}. \tag{440}$$

These magnetic translation operators commute with the Hamiltonian, but do not commute with each other. However, the operators $\hat{t}_{\mathbf{L}_1}, \hat{t}_{q\mathbf{L}_2}$ form a commuting subgroup, $[\hat{t}_{\mathbf{L}_1}, \hat{t}_{q\mathbf{L}_2}] = 0$ and so label the magnetic Brillouin zone (MBZ):

$$\mathbf{k} \in \text{MBZ}, \quad \text{MBZ} = \{\mathbf{k}|\mathbf{k} = 2\pi k_1\mathbf{g}_1 + 2\pi k_2\mathbf{g}_2, \quad k_1 \in [0,1), \quad k_2 \in [0, 1/q)\}. \tag{441}$$

We will now provide a complete basis of states which carry the momentum quantum number $\mathbf{k} \in$ MBZ. This is accomplished by adapting the basis states in 2π flux in Eq.(429). The completeness and orthogonality of the basis in Eq.(429) is an algebraic property that relies on the fact that $\hat{t}_{\mathbf{L}_1}$ and $\hat{t}_{\mathbf{L}_2}$ enclose 2π flux and therefore commute. So in rational flux $\phi = \frac{2\pi p}{q}$, we consider the operators $\hat{t}_{\mathbf{a}_1/p}, \hat{t}_{q\mathbf{L}_2}$ which also enclose 2π flux and commute. Notably, $\hat{t}_{\mathbf{L}_1/p}$ is not a symmetry of the Hamiltonian because it is a translation by a *partial* lattice vector (although it is a well-defined operator). Thus we are led to consider the orthonormal states

$$\left|\tilde{\mathbf{k}}, m\right\rangle = \frac{1}{\sqrt{\mathcal{N}(\tilde{k}_1,\tilde{k}_2)}}\sum_{R_1,R_2\in\mathbb{Z}} e^{-2\pi i(\tilde{k}_1R_1+\tilde{k}_2R_2)}\hat{t}^{R_1}_{\mathbf{L}_1/p}\hat{t}^{R_2}_{q\mathbf{L}_2}\left|m\right\rangle, \quad \left|m\right\rangle = \frac{1}{\sqrt{m!}}a^{\dagger m}\left|0\right\rangle \tag{442}$$

which are parameterized by $\tilde{\mathbf{k}} \in [0,1) \times [0,1)$. To understand the physical meaning of $\tilde{\mathbf{k}}$ in terms of the momentum quantum number $\mathbf{k} \in$ MBZ, we compute

$$\hat{t}_{\mathbf{a}_1}\left|\tilde{\mathbf{k}}, m\right\rangle = \hat{t}^p_{\mathbf{a}_1/p}\left|\tilde{\mathbf{k}}, m\right\rangle = e^{2\pi i p\tilde{k}_1}\left|\tilde{\mathbf{k}}, m\right\rangle, \qquad \implies p\tilde{k}_1 = k_1 \mod 1 \tag{443}$$

$$\hat{t}^q_{\mathbf{a}_2}\left|\tilde{\mathbf{k}}, m\right\rangle = e^{2\pi i\tilde{k}_2}\left|\tilde{\mathbf{k}}, m\right\rangle, \qquad \implies \tilde{k}_2 = qk_2 \mod 1 \tag{444}$$

$$\tag{445}$$

and thus we identify $\tilde{k}_1 = \frac{k_1+\kappa}{p}$, $\kappa = 0,\ldots,p-1$ and $\tilde{k}_2 = qk_2$. Plugging into Eq.(442), we arrive at the following basis of magnetic translation group eigenstates

$$\left|\mathbf{k}, \kappa, m\right\rangle = \frac{1}{\sqrt{\mathcal{N}((k_1+\kappa)/p, qk_2)}}\sum_{R_1,R_2\in\mathbb{Z}} e^{-2\pi i\left(\frac{k_1+\kappa}{p}R_1+qk_2R_2\right)}\hat{t}^{R_1}_{\mathbf{L}_1/p}\hat{t}^{R_2}_{q\mathbf{L}_2}\left|m\right\rangle,$$
$$\mathbf{k} \in \text{MBZ}, \ \kappa = 0,\ldots,p-1 \mod p\,. \tag{446}$$

We see that the basis of states are labeled by $\mathbf{k}$ in the MBZ and an additional flavor index $\kappa = 0,\ldots,p-1$. This index has a simple physical interpretation: consider the Landau level limit of zero crystalline potential so that each momentum in the MBZ is p-fold degenerate due to κ. This means there are p states per magnetic unit cell $\mathbf{a}_1 \times q\mathbf{a}_2$, leading to an electron density of $p/q = \phi/2\pi$ per unit cell. This is expected because Landau levels have a density of $\nu = p/q = \frac{\phi}{2\pi}$. For later reference, we remark that the eigenstates Eq.(446) obey the embedding property

$$\left|\mathbf{k} + \frac{1}{2\pi}\mathbf{g}_1, \kappa, m\right\rangle = \left|\mathbf{k}, \kappa+1, m\right\rangle, \quad \left|\mathbf{k} + \frac{1}{2\pi q}\mathbf{g}_2, \kappa, m\right\rangle = \left|\mathbf{k}, \kappa, m\right\rangle \tag{447}$$

over the MBZ. These eigenstates are similar to the light fermions used to describe the conduction electrons in flux, Eq.(30). The orthonormality $\langle\tilde{\mathbf{k}}, m|\tilde{\mathbf{k}}', n\rangle = \delta(\tilde{\mathbf{k}} - \tilde{\mathbf{k}}')\delta_{mn}$ of the states Eq.(442) is used to calculate the orthonormality of the new states $|\mathbf{k}, \kappa, m\rangle$ by employing the previously derived identification

$$\tilde{k}_1 = \frac{k_1+\kappa}{p}, \ \kappa = 0,\ldots,p-1, \ \ \tilde{k}_2 = qk_2, \tag{448}$$

$$\delta(\tilde{k}_1 - \tilde{k}_1')\delta(\tilde{k}_2 - \tilde{k}_2') = \delta(\frac{k_1+\kappa}{p} - \frac{k_1'+\kappa'}{p})\delta(qk_2 - qk_2') = \frac{p}{q}\delta(k_1 - k_1')\delta(k_2 - k_2')\delta_{\kappa\kappa'}, \tag{449}$$

giving overlap

$$\langle\mathbf{k}, \kappa, m|\mathbf{k}', \kappa', n\rangle = \delta((k_1 - k_1')/p)\delta_{\kappa\kappa'}\delta(q(k_2 - k_2'))\delta_{mn} = \tfrac{p}{q}\delta(k_1 - k_1')\delta(k_2 - k_2')\delta_{\kappa\kappa'}\delta_{mn} \tag{450}$$

where the factor of $p/q = \phi/2\pi$ shows that the density of the states changes with flux. This dependence of density of states on flux is equivalent to the Streda formula $\nu = C\frac{\phi}{2\pi}$ with $C = 1$ for the Landau level state $|\mathbf{k}, \kappa, m\rangle$.

Given a periodic Hamiltonian in magnetic flux,

$$H = h(\boldsymbol{\pi}) + V(\mathbf{r}), \qquad V(\mathbf{r} + \mathbf{a}_i) = V(\mathbf{r}) \tag{451}$$

where h is the kinetic energy, a function of canonical momentum, and V is the lattice-periodic potential energy, one can compute the matrix elements of this Hamiltonian $H^{\phi}_{m\kappa,n\kappa'}(\mathbf{k})$ in the basis Eq.(446):

$$\langle \mathbf{k}, \kappa, m|H(\mathbf{r})|\mathbf{k}', \kappa', n\rangle = \frac{p}{q}\delta(k_1 - k_1')\delta(k_2 - k_2')H^{\phi}_{m\kappa,n\kappa'}(\mathbf{k}). \tag{452}$$

The kinetic term $h(\boldsymbol{\pi})$ has a simple action on the $|\mathbf{k}, \kappa, n\rangle$ states because it is $\mathbf{k}$-independent:

$$h(\boldsymbol{\pi}) = \hbar v_F \sqrt{2B}\begin{bmatrix} 0 & a^\dagger \\ a & 0 \end{bmatrix} \tag{453}$$

$$\langle \mathbf{k}', \kappa', m|a^\dagger|\mathbf{k}, \kappa, n\rangle = \frac{p}{q}\delta(k_1 - k_1')\delta(k_2 - k_2')\ \sqrt{n+1}\delta_{\kappa\kappa'}\delta_{mn}, \tag{454}$$

calculated by observing that $a^\dagger$ commutes with the magnetic translation operators:

$$a^\dagger\,|\mathbf{k}, \kappa, m\rangle = \frac{1}{\sqrt{\mathcal{N}((k_1 + 2\pi\kappa)/p, qk_2)}} \sum_{R_1, R_2 \in \mathbb{Z}} e^{-i\left(\frac{k_1+2\pi\kappa}{p}R_1 + qk_2R_2\right)} T^{R_1}_{\mathbf{L}_1/p} T^{R_2}_{q\mathbf{L}_2} a^\dagger\,|m\rangle\,, \tag{455}$$

$$= \frac{1}{\sqrt{\mathcal{N}((k_1 + 2\pi\kappa)/p, qk_2)}} \sum_{R_1, R_2 \in \mathbb{Z}} e^{-i\left(\frac{k_1+2\pi\kappa}{p}R_1 + qk_2R_2\right)} T^{R_1}_{\mathbf{L}_1/p} T^{R_2}_{q\mathbf{L}_2} \sqrt{m+1}\,|m+1\rangle \tag{456}$$

$$= \sqrt{m+1}\,|\mathbf{k}, \kappa, m+1\rangle\,. \tag{457}$$

We now move on to the an explicit expression for the potential term $V(\mathbf{r})$ containing $e^{2\pi i\mathbf{b}\cdot\mathbf{r}}$ operators. Let us first focus on the case where $p = 1$, where many of the formulae simplify greatly. We recall the formula at $\phi = 2\pi$ [7]:

$$\langle \mathbf{k}, m|e^{-2\pi i\mathbf{b}\cdot\mathbf{r}}|\mathbf{k}', n\rangle = (2\pi)^2\delta(\mathbf{k} - \mathbf{k}')\exp\{(-i\pi b_1b_2 - i\epsilon_{ij}b_ik_j)\}[\exp\Big(i\epsilon_{ij}2\pi b_i\tilde{Z}_j\Big)]_{mn}, \tag{458}$$

where the matrix $\tilde{Z}$ on the Landau level indices (derived for all ϕ in [7]) is

$$Z_j = \frac{\bar{z}_j a + z_j a^\dagger}{\sqrt{2\phi}}, \quad [\tilde{Z}_j]_{mn} = \langle m|Z_j|n\rangle, z_j = \frac{(\hat{x} + i\hat{y})\cdot\mathbf{a}_j}{|\mathbf{a}_1 \times \mathbf{a}_2|}\,. \tag{459}$$

Note that $\tilde{Z}_j$ is a sparse matrix since $a, a^\dagger$ are sparse (each row has only one nonzero element). At $\phi = \frac{2\pi}{q}$, we take $\mathbf{a}_2 \to q\mathbf{a}_2$ in Eq.(458), again employing our identification $k_1 \to k_1, k_2 \to qk_2, G_1 \to G_1, G_2 \to qG_2$ to obtain

$$\langle \mathbf{k}, m|e^{-i\mathbf{G}\cdot\mathbf{r}}|\mathbf{k}', n\rangle = \tfrac{(2\pi)^2}{q}\delta(k_1 - k_1')\delta(k_2 - k_2')\exp\Big\{\big(-i\tfrac{q}{2\pi}(\tfrac{1}{2}G_1G_2 + \epsilon_{ij}G_ik_j)\big)\Big\}[e^{i\epsilon_{ij}G_i\tilde{Z}_j}]_{mn},$$
$$k \in \text{MBZ},\ \mathbf{G}\cdot\mathbf{a}_i = 0 \mod 2\pi \tag{460}$$

$$= (2\pi)^2\tfrac{\phi}{2\pi}\delta(k_1 - k_1')\delta(k_2 - k_2')\exp\left(-\tfrac{i}{\phi}(\tfrac{1}{2}G_1G_2 + \epsilon_{ij}G_ik_j)\right)[e^{i\epsilon_{ij}G_i\tilde{Z}_j}]_{mn} \tag{461}$$

Note that from Eq.(460), $H^{\phi}(k_1, k_2) = H^{\phi}(k_1 + 1, k_2) = H^{\phi}(k_1, k_2 + 1/q)$ is explicitly periodic across the magnetic BZ, and additionally $H^{\phi}(k_1, k_2) = H^{\phi}(k_1 + 1/q, k_2)$ which proves immediately that $\epsilon_n(\mathbf{k}) = \epsilon_n(\mathbf{k} + \frac{1}{q}\mathbf{b}_i)$ as follows from the properties of the magnetic translation group [9, 10].

The simple form for the potential for $p = 1$ becomes significantly more complex when $p \neq 1$. One replaces the formula Eq.(434) as $\mathbf{L}_1 \to \mathbf{L}_1/p, \mathbf{L}_2 \to q\mathbf{L}_2$, to yield the overlap

$$\langle \mathbf{k}', \kappa', m|e^{-i\mathbf{q}\cdot\mathbf{r}}|\mathbf{k}, \kappa, n\rangle = \frac{p}{q}\delta(k_1 - k_1' - q_1/2\pi + \kappa - \kappa')\delta(k_2 - k_2' - q_2/2\pi)$$
$$\times\, e^{i\xi_{q_1/p + iqq_2}((k_1+\kappa)/p, qk_2)}\exp\Big(i\epsilon_{ij}q_i\tilde{Z}_j\Big)_{mn}. \tag{462}$$

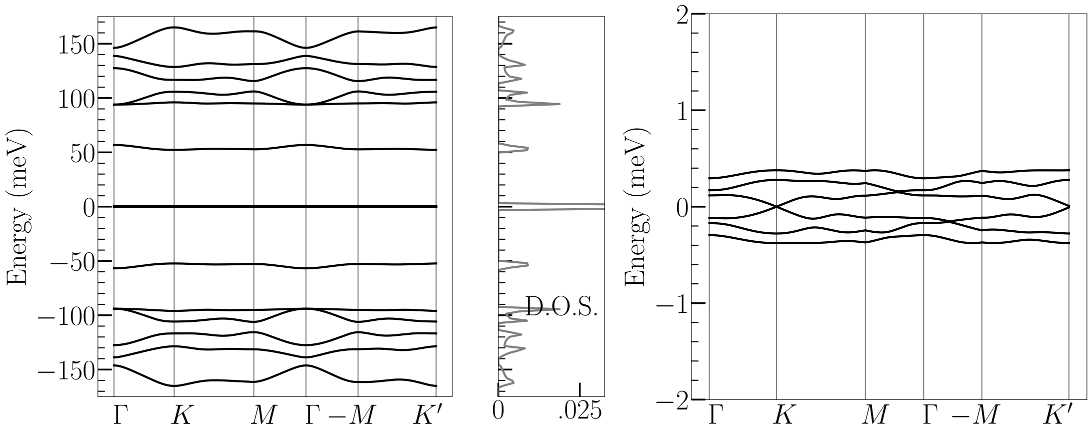

Supplementary Figure 19. Band structures of the BM model at flux $\phi = 2\pi/3$, calculated with our momentum eigenstates. *Left.* The Hofstadter bands show significant flattening over the MBZ, leading to pronounced peaks in the density of states (*Center*). *Right.* Zoom-in of the Hofstadter splitting of the flat bands.

where the phase factor is

$$e^{i\xi_{q_1/p+iqq_2}((k_1+\kappa)/p,qk_2)} = \frac{e^{-\frac{\tilde{q}\bar{\tilde{q}}}{8\pi}}\vartheta\left((k_1+\kappa)/p - \tilde{q}/4\pi, qk_2 + i\tilde{q}/4\pi \middle| \Phi\right)}{\sqrt{\vartheta\left((k_1+\kappa)/p, qk_2 \middle| \Phi\right)\vartheta\left((k_1+\kappa-q_1/2\pi)/p, q(k_2-q_2/2\pi)\middle| \Phi\right)}}, \quad \tilde{q} = q_1/p + iqq_2. \tag{463}$$

In practice, we will not need to full expression for the phase in Eq.(462). To evaluate the Hamiltonian, we only need an expression for the overlap at $\mathbf{q} = \mathbf{G}$, a reciprocal lattice vector. In this case, the expression simplifies to

$$\begin{aligned}\langle \mathbf{k}', \kappa', m|e^{-i\mathbf{G}\cdot\mathbf{r}}|\mathbf{k}, \kappa, n\rangle &= \tfrac{p}{q}\delta(k_1-k_1')\delta_{\kappa,\kappa'+G_1}\delta(k_2-k_2') \\ &\times e^{i\xi_{G_1/p+iqG_2}((k_1+\kappa)/p,qk_2)}\exp\left(i\epsilon_{ij}G_i\tilde{Z}_j\right)_{mn}\end{aligned} \tag{464}$$

To calculate the matrix elements of the BM model, we first perform a gauge transformation to recast the Hamiltonian into an explicitly translation-symmetric form. Because $\mathbf{q}_1, \mathbf{q}_2, \mathbf{q}_3$ are not reciprocal lattice vectors, the BM model is only periodic up to a gauge transformation. Conjugating H_{BM} by $e^{-i\pi\mathbf{q}_3\cdot\mathbf{r}}$ restores translation symmetry, giving Hamiltonian

$$\tilde{H}_{\mathrm{BM}} = e^{\pi i\mathbf{q}_3\cdot\mathbf{r}}H_{\mathrm{BM}}e^{-\pi i\mathbf{q}_3\cdot\mathbf{r}} = \begin{bmatrix} -i\hbar v_F\boldsymbol{\sigma}\cdot\boldsymbol{\nabla} - \pi\hbar v_F\mathbf{q}_3\cdot\boldsymbol{\sigma} & \tilde{T}^\dagger(\mathbf{r}) \\ \tilde{T}(\mathbf{r}) & -i\hbar v_F\boldsymbol{\sigma}\cdot\boldsymbol{\nabla} + \pi\hbar v_F\mathbf{q}_3\cdot\boldsymbol{\sigma}\end{bmatrix}, \tag{465}$$

$$\tilde{T}(\mathbf{r}) = \sum_{j=1}^{3} e^{2\pi i\mathbf{g}_1\cdot\mathbf{r}}T_1 + e^{2\pi i\mathbf{g}_2\cdot\mathbf{r}}T_2 + T_3, \quad T_{j+1} = w_0\sigma_0 + w_1\left(\sigma_1\cos\frac{2\pi j}{3} + \sigma_2\sin\frac{2\pi j}{3}\right). \tag{466}$$

. Upon canonical substitution, the Hamiltonian in flux reads

$$\tilde{H}_{\mathrm{BM}}(\phi) = \begin{bmatrix} \hbar v_F\boldsymbol{\sigma}\cdot\boldsymbol{\pi} - \pi\hbar v_F\mathbf{q}_3\cdot\boldsymbol{\sigma} & \tilde{T}^\dagger(\mathbf{r}) \\ \tilde{T}(\mathbf{r}) & \hbar v_F\boldsymbol{\sigma}\cdot\boldsymbol{\pi} + \pi\hbar v_F\mathbf{q}_3\cdot\boldsymbol{\sigma}\end{bmatrix}, \tag{467}$$

An example band structure at flux $\phi = 2\pi/3$ calculated with our technique is depicted in Supplementary Fig.(19).

A. Groundstate Charge Density

A key feature of the HF model of TBG is the concentration of charge at the moiré unit cell center in the non-interacting model. This is one indication that the Wannierization must contain orbitals at the $1a$ position. To check

the validity of the HF approximation in small flux, we will compute the charge density of the groundstate in flux $\phi = \frac{2\pi}{q}$, for this section restricting to $p = 1$ for convenience. We will compute the quantity

$$\langle GS|n(\mathbf{r})|GS\rangle = \sum_{l,\alpha,\eta,s} \langle GS|c^\dagger_{l,\alpha,\eta,s}(\mathbf{r})c_{l,\alpha,\eta,s}(\mathbf{r})|GS\rangle \tag{468}$$

$$= \frac{1}{q\Omega}\sum_{\mathbf{G}_q}\int_{q_1=0}^{2\pi}\int_{q_2=0}^{2\pi/q}\frac{d^2q}{(2\pi)^2/q}e^{i(\mathbf{q}+\mathbf{G}_q)\cdot\mathbf{r}}\langle GS|\rho_{\mathbf{q}+\mathbf{G}_q}|GS\rangle \tag{469}$$

where $|GS\rangle = \prod_j \prod_{\mathbf{k},n,\eta_j,s_j} \gamma^\dagger_{\mathbf{k},n,\eta_j,s_j}|0\rangle$ is the Slater determinant state filling all the $2q$ Hofstadter flat bands and $n(\mathbf{r}) = \sum_{l,\alpha,\eta,s} c^\dagger_{l,\alpha,\eta,s}(\mathbf{r})c_{l,\alpha,\eta,s}(\mathbf{r})$ is the local charge operator summed over layer l, sublattice α, valley η, and spin s. The vectors $\mathbf{G}_q = 2\pi\mathbb{Z}\mathbf{g}_1 + 2\pi\mathbb{Z}\mathbf{g}_2/q$ are the reciprocal lattice vectors of the MBZ (note that $\mathbf{G}_q \cdot \mathbf{a}_2$ can be fractional). The momentum space density operator ρ is defined as

$$\rho_{\mathbf{q}+\mathbf{G}_q} = \int d^2r e^{-i(\mathbf{q}+\mathbf{G}_q)\cdot\mathbf{r}}\sum_{l,\alpha,\eta,s} c^\dagger_{l,\alpha,\eta,s}(\mathbf{r})c_{l,\alpha,\eta,s}(\mathbf{r}). \tag{470}$$

We first find the matrix elements of this operator in terms of Landau levels. Define the creation operator $\psi^\dagger_{\mathbf{k},m,\alpha,l,\eta,s}$ as the operator that creates the state $|\mathbf{k},m,\alpha,l,\eta,s\rangle$. Following the calculations from Ref. [7] gives the following relations:

$$\langle\mathbf{k}',\alpha,l,m,\eta,s|c^\dagger_{l,\alpha,\eta,s}(\mathbf{r})c_{l,\alpha,\eta,s}(\mathbf{r})|\mathbf{k},\alpha,l,n,\eta,s\rangle = \langle 0|\psi_{\mathbf{k}',\alpha,l,m,\eta,s}c^\dagger_{l,\alpha,\eta,s}(\mathbf{r})c_{l,\alpha,\eta,s}(\mathbf{r})\psi^\dagger_{\mathbf{k},\alpha,l,n,\eta,s}|0\rangle \tag{471}$$

$$= \langle 0|\psi_{\mathbf{k}',\alpha,l,m,\eta,s}c^\dagger_{l,\alpha,\eta,s}(\mathbf{r})|0\rangle\langle 0|c_{l,\alpha,\eta,s}(\mathbf{r})\psi^\dagger_{\mathbf{k},\alpha,l,n,\eta,s}|0\rangle \tag{472}$$

$$= \psi_{\mathbf{k}',\alpha,l,m,\eta,s}(\mathbf{r})\psi^*_{\mathbf{k},\alpha,l,n,\eta,s}(\mathbf{r}), \tag{473}$$

where $\psi_{\mathbf{k}',\alpha,l,m,\eta,s}(\mathbf{r})$ is the real-space wavefunction corresponding to the momentum ket $|\mathbf{k}',\alpha,l,m,\eta,s\rangle$. These wavefunctions are orthonormal, and integrating over $\mathbf{r}$ gives

$$\int d^2r e^{-i\mathbf{q}\cdot\mathbf{r}}\psi_{\mathbf{k}',\alpha,l,m,\eta,s}(\mathbf{r})\psi^*_{\mathbf{k},\alpha,l,n,\eta,s}(\mathbf{r}) = \langle\mathbf{k}',\alpha,l,m,\eta,s|e^{-i\mathbf{q}\cdot\mathbf{r}}|\mathbf{k},\alpha,l,n,\eta,s\rangle \tag{474}$$

The density operator becomes

$$\begin{aligned}\rho_{\mathbf{q}+\mathbf{G}_q} &= \int d^2r\sum_{\alpha,l,\eta,s}\sum_{mn}\int\frac{d^2kd^2k'}{1/q^2}e^{-i(\mathbf{q}+\mathbf{G}_q)\cdot\mathbf{r}}\\ &\times\langle\mathbf{k}',\alpha,l,m,\eta,s|c^\dagger_{l,\alpha,\eta,s}(\mathbf{r})c_{l,\alpha,\eta,s}(\mathbf{r})|\mathbf{k},\alpha,l,n,\eta,s\rangle\psi^\dagger_{\mathbf{k}',\alpha,l,m,\eta,s}\psi_{\mathbf{k},\alpha,l,n,\eta,s}\\ &= \sum_{\alpha,l,\eta,s}\sum_{mn}\int\frac{d^2kd^2k'}{1/q^2}\langle\mathbf{k}',m,\alpha,l,\eta,s|e^{-i(\mathbf{q}+\mathbf{G}_q)\cdot\mathbf{r}}|\mathbf{k},n,\alpha,l,\eta,s\rangle\psi^\dagger_{\mathbf{k}',\alpha,l,m,\eta,s}\psi_{\mathbf{k},\alpha,l,n,\eta,s},\end{aligned} \tag{475}$$

where the form factor $\langle\mathbf{k}',m|e^{-i(\mathbf{q}+\mathbf{G}_q)\cdot\mathbf{r}}|\mathbf{k},n\rangle$ has been defined in Eq.(462). The factor of $1/q^2$ in the integrand arises from the fact that working at $\phi = 2\pi/q$, the Brillouin zone size has reduced by a factor of q.

We project into the flat bands, defining the eigenoperators $\gamma^\dagger_{\mathbf{k},M,\eta,s}$ as the operators that create the Mth energy eigenstate at momentum $\mathbf{k}$, with M ranging from 1 to $2q$. To do this, expand the eigenoperators as

$$\gamma_{\mathbf{k},N,\eta,s} = \sum_{\alpha,l,m}[U^{(\eta,s)}]^N_{\alpha,l,m}(\mathbf{k})\psi_{\mathbf{k},\alpha,l,m,\eta,s}, \tag{476}$$

so upon projection

$$\psi^\dagger_{\mathbf{k}',\alpha,l,m,\eta,s}\psi_{\mathbf{k},\alpha,l,n,\eta,s} \to \sum_{MN}[U^{(\eta,s)*}]^M_{\alpha,l,m}(\mathbf{k}')[U^{(\eta,s)}]^N_{\alpha,l,n}(\mathbf{k})\gamma^\dagger_{\mathbf{k}',M,\eta,s}\gamma_{\mathbf{k},N,\eta,s} \tag{477}$$

The projected form factors read

$$\rho_{\mathbf{q}} \to \rho_{\mathbf{q}+\mathbf{G}_q} = \int_{[0,1)\times[0,1/q)}\frac{d^2k}{1/q}\sum_{MN}M^{(\eta,s)}_{mn}(\mathbf{k},\mathbf{q}+\mathbf{G}_q)\gamma^\dagger_{\mathbf{k}-\mathbf{q}/2\pi,M,\eta_j,s_j}\gamma_{\mathbf{k},N,\eta_j,s_j}, \tag{478}$$

where $M^{(\eta,s)}(\mathbf{k},\mathbf{q})$ are the form factor matrices given by

$$M^{(\eta,s)}_{MN}(\mathbf{k},\mathbf{q}+\mathbf{G}_q) = \sum_{\alpha,l,\eta,s,mn} e^{i\xi_{\mathbf{q}+\mathbf{G_q}}(\mathbf{k})}[U^{(\eta,s)}{}^{\dagger}_{\alpha,l,n}(\mathbf{k}-\mathbf{q})\mathcal{H}^{\mathbf{q}+\mathbf{G}_q}_{mn}U^{(\eta,s)}_{\alpha,l,n}(\mathbf{k})]_{MN}, \tag{479}$$

$$\frac{1}{q}\delta(k_1-k_1')\delta(k_2-k_2')e^{i\xi_{\mathbf{q}+\mathbf{G_q}}(\mathbf{k})}\mathcal{H}^{\mathbf{q}+\mathbf{G}_q}_{mn} = \langle \mathbf{k}',m|e^{-i(\mathbf{q}+\mathbf{G}_q)\cdot\mathbf{r}}|\mathbf{k},n\rangle \tag{480}$$

with $[U^{(\eta,s)}]^N_{\alpha,l,m}(\mathbf{k})$ the wavefunction of the Nth energy band in the mth Landau level, with repeated indices summed. We have suppressed the indices α,l,η,s in the ket $|\mathbf{k},m\rangle$ as they are unchanged by the operator $e^{-i\mathbf{q}\cdot\mathbf{r}}$.

When computing many-body overlaps, it is more convenient to discretize the magnetic BZ and work with Kronecker delta normalized fermion operators instead of Dirac delta normalized operators. To do so, we discretize according to the normalization

$$\{\frac{1}{\sqrt{N}}\gamma_{\mathbf{k},m,\eta_j,s_j},\frac{1}{\sqrt{N}}\gamma^{\dagger}_{\mathbf{k}',n,\eta_{j'},s_{j'}}\} = \tfrac{1}{N}\tfrac{1}{q}\delta(\mathbf{k}-\mathbf{k}')\delta_{mn}\delta_{jj'} \to \delta_{\mathbf{k}\mathbf{k}'}\delta_{mn}\delta_{jj'} \tag{481}$$

where $N \to \delta^2(0)$ is the number of unit cells. Equivalently, N is the number of magnetic unit cells which is equal to the number of k-points in the magnetic BZ. Working in momentum space, we discretize the momentum

$$\begin{aligned}\rho_{\mathbf{q}+\mathbf{G}_q} &= \textstyle\int_{[0,1)\times[0,1/q)}\frac{d^2k}{1/q}\sum_{MN} M^{(\eta,s)}_{MN}(\mathbf{k},\mathbf{q}/2\pi+\mathbf{G}_q)\gamma^{\dagger}_{\mathbf{k}-\mathbf{q}/2\pi,M,\eta_j,s_j}\gamma_{\mathbf{k},N,\eta_j,s_j}\\ &\to \textstyle\sum_{\mathbf{k}MN} M^{(\eta,s)}_{MN}(\mathbf{k},\mathbf{q}/2\pi+\mathbf{G}_q)\gamma^{\dagger}_{\mathbf{k}-\mathbf{q}/2\pi,M,\eta_j,s_j}\gamma_{\mathbf{k},N,\eta_j,s_j}\end{aligned} \tag{482}$$

where M,N sum over all the Hofstadter bands and the arrow indicates a discretization of the the magnetic BZ.

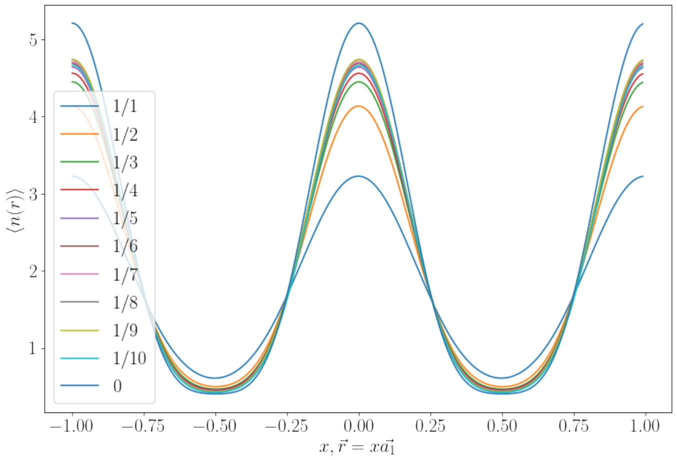

Supplementary Figure 20. Real space cuts along the $\mathbf{L}_1$ lattice vector. All fluxes have many-body charge densities that are peaked at the $1a$ location, but the 0 flux is the most peaked while the 2π is the most spread. As flux is increased, the wavefunction weight is increasingly spread, but remains strongly localized at $1a$. This validates the use of the HF Hamiltonian in flux.

Acting on the groundstate, we have

$$\langle GS|\,\rho_{\mathbf{q}+\mathbf{G}_q}\,|GS\rangle \;= \delta_{\mathbf{q},0}\,\langle GS|\,\rho_{\mathbf{G}_q}\,|GS\rangle = \delta_{\mathbf{q},0}\textstyle\sum_{\mathbf{k}} \mathrm{Tr}\, M(\mathbf{k},\mathbf{G}_q) \tag{483}$$

$$\langle GS|\,\rho_{\mathbf{q}+\mathbf{G}_q}\,|GS\rangle = \tfrac{(2\pi)^2}{q}\delta(q_1)\delta(q_2)\textstyle\int_{[0,1)\times[0,1/q)}\frac{d^2k}{1/q}\,\mathrm{Tr}\, M(\mathbf{k},\mathbf{G}_q) \tag{484}$$

switching back to the continuum normalization in the second line. As $\mathbf{q}$ ranges over the MBZ, the continuum delta function $\delta(q_1)\delta(q_2)$ appears with a prefactor of $(2\pi)^2/q$, so that the following integral over the MBZ is normalized to 1:

$$\int_{[0,2\pi)\times[0,2\pi/q)}\frac{d^2q}{(2\pi)^2/q}\frac{(2\pi)^2}{q}\delta(q_1)\delta(q_2) = 1. \tag{485}$$

We now prove that only $\langle GS|\,\rho_{\mathbf{G}_q}\,|GS\rangle$ is nonzero, for $\mathbf{G}_q$ a reciprocal lattice vector of the zero-flux (non-magnetic) BZ: that is, $\langle GS|\,\rho_{\mathbf{G}_q}\,|GS\rangle$ is nonzero only if $\mathbf{G}_q = 2\pi\mathbb{Z}\mathbf{g}_1 + 2\pi\mathbb{Z}\mathbf{g}_2$. This follows from the periodicity of the form factors:

$$M_{mn}(\mathbf{k}+\mathbf{b}_1/q,\mathbf{G}_q) = e^{i\mathbf{G}_q\cdot\mathbf{a}_2}M_{mn}(\mathbf{k},\mathbf{G}_q) \tag{486}$$

which is derived from the form factor definition:

$$\frac{1}{q}\delta(k_1-k_1')\delta(k_2-k_2')e^{i\xi_{\mathbf{G}_q}(\mathbf{k}+\mathbf{g}_1/2\pi q)}\mathcal{H}^{\mathbf{G}_q}_{mn} = \langle \mathbf{k}+\mathbf{g}_1/2\pi q, m|e^{-2\pi i \mathbf{G}_q\cdot\mathbf{r}}|\mathbf{k}'+\mathbf{g}_1/2\pi q, n\rangle \tag{487}$$

$$= \frac{1}{q}\delta(k_1-k_1')\delta(k_2-k_2')e^{-iq(\pi(G_q)_1(G_q)_2+\epsilon_{ij}(G_q)_i k_j - 2\pi(G_q)_2/q)}[e^{i\epsilon_{ij}2\pi(G_q)_i\tilde{Z}_j}]_{mn} \tag{488}$$

$$= e^{2\pi i\mathbf{G}_q\cdot\mathbf{L}_2}\langle \mathbf{k},m|e^{-2\pi i\mathbf{G}_q\cdot\mathbf{r}}|\mathbf{k}',n\rangle, \tag{489}$$

or

$$\langle \mathbf{k}+\mathbf{g}_1/2\pi q, m|e^{-2\pi i\mathbf{G}_q\cdot\mathbf{r}}|\mathbf{k}'+\mathbf{g}_1/2\pi q, n\rangle = e^{2\pi i\mathbf{G}_q\cdot\mathbf{L}_2}\langle \mathbf{k},m|e^{-2\pi i\mathbf{G}_q\cdot\mathbf{r}}|\mathbf{k}',n\rangle \tag{490}$$

so that

$$\int \frac{d^2k}{1/q}\,\mathrm{Tr}\,M(\mathbf{k},\mathbf{G}_q) = \int_0^1 \frac{dk_1}{2\pi}\int_0^{1/q}\frac{dk_2}{1/q}\,\mathrm{Tr}\,M(\mathbf{k},\mathbf{G}_q) \tag{491}$$

$$= \left(\sum_{j=0}^{q-1} e^{ij\mathbf{G}\cdot\mathbf{a}_2}\right)\int_0^{1/q} dk_1\int_0^{1/q}\frac{dk_2}{1/q}\,\mathrm{Tr}\,M(\mathbf{k},\mathbf{G}) \tag{492}$$

$$= \delta_{\mathbf{G_q},\mathbf{G}}\int_0^{1/q}\frac{dk_1}{1/q}\int_0^{1/q}\frac{dk_2}{1/q}\,\mathrm{Tr}\,M(\mathbf{k},\mathbf{G}_q) \tag{493}$$

because $\sum_{j=0}^{q-1} e^{ij\mathbf{G}_q\cdot\mathbf{a}_2} = q$ if $\mathbf{G}_q\cdot\mathbf{L}_2 \in \mathbb{Z}$, and otherwise vanishes: that is, only if $\mathbf{G}_q$ is a reciprocal lattice vector. This observation allows significant speedup in numerical calculations, as $\mathbf{G}_q$ vectors that are not reciprocal lattice vectors do not need to be calculated in the expression for the many-body charge density. The integrals in Eq.(483) will appear throughout the many-body calculations, so we find it convenient to define

$$n_{\mathbf{G}} = \int_{[0,1)\times[0,1/q)}\frac{d^2k}{1/q}\frac{1}{q}\,\mathrm{Tr}\,M(\mathbf{k},\mathbf{G}), \qquad \mathbf{G}\in 2\pi\mathbf{b}_1\mathbb{Z}+2\pi\mathbf{b}_2\mathbb{Z} \tag{494}$$

which is the average of the form factor over the magnetic BZ and (the $2q$) occupied Hofstadter bands. Physically, $n_{\tilde{\mathbf{G}}}$ are the Fourier modes of the charge distribution, which we see from plugging into Eq.(468):

$$\langle GS|n(\mathbf{r})|GS\rangle = \sum_{\tilde{\mathbf{G}}} e^{i\mathbf{G}\cdot\mathbf{r}}\int\frac{d^2k}{(2\pi)^2\Omega/q}\frac{1}{q}\,\mathrm{Tr}\,M(\mathbf{k},\mathbf{G}) = \sum_{\mathbf{G}} e^{i\mathbf{G}\cdot\mathbf{r}}\int\frac{d^2k}{\Omega/q}\frac{1}{q}\,\mathrm{Tr}\,M(\mathbf{k},\mathbf{G}) = \frac{1}{\Omega}\sum_{\mathbf{G}} e^{i\mathbf{G}\cdot\mathbf{r}}n_{\mathbf{G}}\ . \tag{495}$$

By proving that $n_{\mathbf{G}}$ vanishes if $\mathbf{G}$ is not a reciprocal lattice vector, we have shown that the charge distribution at rational flux is periodic over the physical unit cell, not just over the (extended) magnetic unit cell. A figure illustrating the expectation value over a slice in real space along the $\mathbf{L}_1$ direction is depicted in Supplementary Fig.(20). We see that even in the presence of flux, the charge density remains localized at the $1a$ position, though the spread does increase slightly with flux.

B. Many-body Charge Excitations

It was realized in [11] that the double commutator calculation could be extended to TBG in flux. In this appendix, we detail a variation of this calculation using our gauge invariant formalism [7, 8], which makes the derivation of the charge excitations very simple. First we recall some basic results from the strong coupling theory of TBG: the double-commutator technique of [12, 13] will be used extensively in this section, along with results from [7, 14]. Starting with a filled-band groundstate ansatz [15–17] at filling ν ($\nu=0$ is the charge neutrality point) given by

$$|\Psi_\nu\rangle = \prod_{\mathbf{k},n}\prod_{j=1}^{\nu+4}\gamma^\dagger_{\mathbf{k},n,\eta_j,s_j}|0\rangle \tag{496}$$

in spin-valley flavors $\{s_j\},\{\eta_j\}$, we compute the excitations of the Coulomb Hamiltonian projected into $2q$ flat bands (per spin-valley) defined by

$$H_{int} = \frac{1}{2}\int\frac{d^2q}{(2\pi)^2}V(\mathbf{q})\bar{\rho}_{\mathbf{q}}\bar{\rho}_{-\mathbf{q}} = \frac{1}{2}\sum_{\mathbf{G},j=0}^{q-1}\int_{(0,2\pi)\times(0,2\pi/q)}\frac{d^2q}{(2\pi)^2}\frac{V(\mathbf{q}+\frac{2\pi j}{q}\mathbf{b}_2+\mathbf{G})}{\Omega}\bar{\rho}_{\mathbf{q}+\frac{2\pi j}{q}\mathbf{b}_2+\mathbf{G}}\bar{\rho}_{-(\mathbf{q}+\frac{2\pi j}{q}\mathbf{b}_2+\mathbf{G})} \tag{497}$$

$$\bar{\rho}_{\mathbf{q}} = \sum_{\mathbf{k},MN,\eta s} M^{(\eta,s)}_{MN}(\mathbf{k},\mathbf{q})(\gamma^\dagger_{\mathbf{k}-\mathbf{q},M,\eta,s}\gamma_{\mathbf{k},N,\eta,s} - \frac{1}{2}\delta_{\mathbf{q},0}\delta_{MN}). \tag{498}$$

Working at $p=1$, the equation breaks up the momentum $\mathbf{q}$ by restricting $\mathbf{q}$ into a vector in the MBZ, then adding a multiple of $\mathbf{g}_2/q$, and a reciprocal lattice vector $\mathbf{G}$, so that the total momentum $\mathbf{q}+\frac{2\pi j}{q}\mathbf{g}_2+\mathbf{G}$.

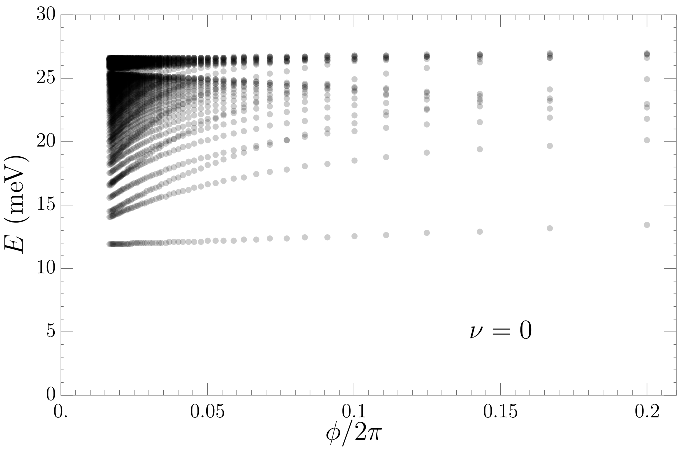

Supplementary Figure 21. Charge +1 excitations above the $\nu = 0$ VP state in flux, calculated via the strong coupling technique.

At charge neutrality, $\nu = 0$, $|\Psi_\nu\rangle$ is a groundstate of H_{int} because H_{int} is positive semi-definite and

$$\bar{\rho}_{\mathbf{q}+\frac{2\pi}{q}j\mathbf{b}_2+\mathbf{G}} |\Psi_\nu\rangle = \frac{\nu}{2}\frac{(2\pi)^2}{q}\delta(q_1)\delta(q_2)\delta_{j,0}qn_{\mathbf{G}} |\Psi_\nu\rangle \tag{499}$$

and thus $H_{int} |\Psi_{\nu=0}\rangle = 0$. This convention justifies the choice of the projected interaction Hamiltonian. More generally, we observe that

$$H_{int} |\Psi_\nu\rangle = \frac{1}{2}\sum_{\mathbf{G},j=0}^{q-1} \int_{(0,2\pi)\times(0,2\pi/q)} \frac{d^2q}{(2\pi)^2} \frac{V(\mathbf{q}+\frac{2\pi j}{q}\mathbf{b}_2+\mathbf{G})}{\Omega} (\tfrac{\nu}{2})^2((2\pi)^2\delta(q_1)\delta(q_2))^2\delta_{j,0}|n_{\mathbf{G}}|^2 |\Psi_\nu\rangle \tag{500}$$

$$= (2\pi)^2\delta^2(0)\left(\frac{\nu^2}{8}\sum_{\mathbf{G}}\frac{V(\mathbf{G})}{\Omega}|n_{\mathbf{G}}|^2\right)|\Psi_\nu\rangle \tag{501}$$

$$\tag{502}$$

where $(2\pi)^2\delta^2(0)$ is the total number of unit cells. Thus the many-body energy density of $|\Psi_\nu\rangle$ is given by the quantity in parentheses in Eq.(500).

A double-commutator calculation [12, 13] gives the charge ±1 excitations in terms of the following $2q \times 2q$ effective Hamiltonians (the $\phi = 2\pi/q$ dependence is suppressed in $M(\mathbf{k}, \mathbf{q})$ and $n_{\mathbf{G}}$):

$$R^+_{MN}(\mathbf{k}) = \frac{1}{N}\sum_{\mathbf{q},\mathbf{G}} \frac{1}{2}\frac{V(\mathbf{q}+\mathbf{G})}{\Omega}[M^\dagger(\mathbf{k},\mathbf{q}+\mathbf{G})M(\mathbf{k},\mathbf{q}+\mathbf{G})]_{MN} + \frac{\nu}{2}\sum_{\mathbf{G}}\frac{V(\mathbf{G})}{\Omega}[M(\mathbf{k},\mathbf{G})]_{MN}n^*_{\mathbf{G}} - \mu\delta_{MN} \tag{503}$$

$$R^-_{MN}(\mathbf{k})^* = \frac{1}{N}\sum_{\mathbf{q},\mathbf{G}} \frac{1}{2}\frac{V(\mathbf{q}+\mathbf{G})}{\Omega}[M^\dagger(\mathbf{k},\mathbf{q}+\mathbf{G})M(\mathbf{k},\mathbf{q}+\mathbf{G})]_{MN} - \frac{\nu}{2}\sum_{\mathbf{G}}\frac{V(\mathbf{G})}{\Omega}[M(\mathbf{k},\mathbf{G})]_{MN}n^*_{\mathbf{G}} + \mu\delta_{MN} \tag{504}$$

$$\tag{505}$$

where $\mathbf{q}$ sums over the N states in the full Brillouin zone, and the chemical potential $\mu = (E_{min,+1} - E_{min,-1})/2$ is chosen so that the minima of the charge ±1 spectra at $\mu = 0$, denoted $E_{min,+\pm}$, are the same. Physically, this choice of μ corresponds to setting zero energy in the middle of the excitation gap. We refer to the first term in $R^\pm(\mathbf{k})$ as the Fock term. Since $V(\mathbf{q}) > 0$ and $M^\dagger M$ is a positive-semi-definite matrix, the Fock term is positive semi-definite. It also does not depend on the filling ν. Note that the phase $e^{i\xi_{\mathbf{q}}(\mathbf{k})}$ in the form factors $M(\mathbf{k}, \mathbf{q})$ cancels in the $M^\dagger M$ product, and so does not need to be evaluated. To further speed up the numerical calculations, $n_{\mathbf{G}}$ can be precomputed since it does not depend on $\mathbf{k}$. Most importantly, the computation of $M(\mathbf{k}, \mathbf{q})$ can be carried out with sparse matrix multiplication of $e^{i\epsilon_{ij}q_i\tilde{Z}_j}$ on $U(\mathbf{k})$. We find that even for very large q, the computations of $R^\pm(\mathbf{k})$ are feasible taking the Landau level cutoff to be $30q$ and truncating the $\mathbf{G}$ sum after three shells. A plot of the many-body charge excitations calculated with strong coupling is shown in Supplementary Fig.(21); this is compared to the spectrum obtained via heavy fermions to excellent agreement in the main text.

Supplementary note 13. NOTATIONAL CHANGES

- The notation $I^0_{[m,r']}$ is used in the supplementary to represent the c-f hybridization matrix (see Eq.(131)). In the main text, we use the notation $\Upsilon_{[m,r']}$ for the same. It is defined in Eq.(13) of main-text.

- The notation m_{max} is used in the supplementary to represent the upper cut-off on c-LL index. In the main text, we use the notation $m_\star$ for the same.

[1] R. Bistritzer and A. H. MacDonald, Moiré bands in twisted double-layer graphene, Proceedings of the National Academy of Sciences **108**, 12233 (2011).
[2] Z.-D. Song and B. A. Bernevig, Magic-angle twisted bilayer graphene as a topological heavy fermion problem, Physical review letters **129**, 047601 (2022).
[3] X. Wang and O. Vafek, Revisiting bloch electrons in magnetic field: Hofstadter physics via hybrid wannier states, arXiv preprint arXiv:2303.16347 (2023).
[4] J. M. Luttinger and W. Kohn, Motion of electrons and holes in perturbed periodic fields, Physical Review **97**, 869 (1955).
[5] D. Călugăru, M. Borovkov, L. L. Lau, P. Coleman, Z.-D. Song, and B. A. Bernevig, Twisted bilayer graphene as topological heavy fermion: Ii. analytical approximations of the model parameters, Low Temperature Physics **49**, 640 (2023).
[6] D. Babusci, G. Dattoli, and M. Quattromini, On integrals involving hermite polynomials, Applied Mathematics Letters **25**, 1157 (2012).
[7] J. Herzog-Arbeitman, A. Chew, and B. A. Bernevig, Magnetic bloch theorem and reentrant flat bands in twisted bilayer graphene at 2π flux, Phys. Rev. B **106**, 085140 (2022).
[8] J. Herzog-Arbeitman, A. Chew, D. K. Efetov, and B. A. Bernevig, Reentrant correlated insulators in twisted bilayer graphene at 25 t (2π flux), Phys. Rev. Lett. **129**, 076401 (2022).
[9] J. Zak, Magnetic translation group, Phys. Rev. **134**, A1602 (1964).
[10] J. Zak, Magnetic Translation Group. II. Irreducible Representations, Phys. Rev. **134**, A1607 (1964).
[11] X. Wang and O. Vafek, Narrow bands in magnetic field and strong-coupling hofstadter spectra, Physical Review B **106**, L121111 (2022).
[12] O. Vafek and J. Kang, Renormalization group study of hidden symmetry in twisted bilayer graphene with coulomb interactions, Phys. Rev. Lett. **125**, 257602 (2020).
[13] B. A. Bernevig, B. Lian, A. Cowsik, F. Xie, N. Regnault, and Z.-D. Song, Twisted bilayer graphene. v. exact analytic many-body excitations in coulomb hamiltonians: Charge gap, goldstone modes, and absence of cooper pairing, Phys. Rev. B **103**, 205415 (2021).
[14] B. A. Bernevig, Z.-D. Song, N. Regnault, and B. Lian, Twisted bilayer graphene. iii. interacting hamiltonian and exact symmetries, Phys. Rev. B **103**, 205413 (2021).
[15] J. Kang and O. Vafek, Strong coupling phases of partially filled twisted bilayer graphene narrow bands, Phys. Rev. Lett. **122**, 246401 (2019).
[16] N. Bultinck, E. Khalaf, S. Liu, S. Chatterjee, A. Vishwanath, and M. P. Zaletel, Ground state and hidden symmetry of magic-angle graphene at even integer filling, Phys. Rev. X **10**, 031034 (2020).
[17] B. Lian, Z.-D. Song, N. Regnault, D. K. Efetov, A. Yazdani, and B. A. Bernevig, Twisted bilayer graphene. iv. exact insulator ground states and phase diagram, Phys. Rev. B **103**, 205414 (2021).
